# Transcriptomic analysis of the prothoracic gland from two lepidopteran insects, domesticated silkmoth *Bombyx mori* and wild silkmoth *Antheraea pernyi*

**DOI:** 10.1038/s41598-019-41864-0

**Published:** 2019-03-29

**Authors:** Hai-Xu Bian, Dong-Bin Chen, Xi-Xi Zheng, Hong-Fang Ma, Yu-Ping Li, Qun Li, Run-Xi Xia, Huan Wang, Yi-Ren Jiang, Yan-Qun Liu, Li Qin

**Affiliations:** 1 College of Plant Protection, Shenyang Agricultural University, 120 Dongling Road, Shenyang, 110866 China; 2 College of Bioscience and Biotechnology, Shenyang Agricultural University, 120 Dongling Road, Shenyang, 110866 China

## Abstract

The prothoracic gland (PG) is an important endocrine organ of synthesis and secretion of ecdysteroids that play critical roles in insects. Here, we used a comparative transcriptomic approach to characterize some common features of PGs from two lepidopteran species *Bombyx mori* and *Antheraea pernyi*. Functional and pathway annotations revealed an overall similarity in gene profile between the two PG transcriptomes. As expected, almost all steroid hormone biosynthesis genes and the prothoracicitropic hormone receptor gene (*Torso*) were well represented in the two PGs. Impressively, two ecdysone receptor genes, eleven juvenile hormone related genes, more than 10 chemosensory protein genes, and a set of genes involved in circadian clock were also presented in the two PGs. Quantitative real time -PCR (qRT-PCR) validated the expression of 8 juvenile hormone and 12 clock related genes in *B. mori* PG, and revealed a different expression pattern during development in whole fifth larval instar. This contribution to insect PG transcriptome data will extend our understanding of the function and regulation of this important organ.

## Introduction

The prothoracic gland (PG) of insect is one of the most important endocrine organs that synthesizes and releases ecdysteroid hormone playing critical roles in regulating growth, moulting and metamorphosis. The insect PG is a CPU-like “decision-making center” that integrates a wide range of systemic cues before permitting the production of an ecdysone pulse^1^. In higher Diptera *Drosophila* the PG, together with the corpus cardiacum (CC) and corpus allatum (CA), is fused into a complex endocrine structure, known as ring gland. However, in other insects, including the domesticated silkworm *Bombyx mori* (Lepidoptera: Bombycidae) and wild silkworm *Antheraea pernyi* (Lepidoptera: Saturniidae), these three endocrine glands form separate structures. In *B. mori* and *A. pernyi*, the PGs are a pair of semi-transparent or transparent saccate cell clusters with conjunct theca, respectively, located in the tracheal clusters of the prothorax.

As an important endocrine organ, the insect PG has been considered as a model for steroid hormone biosynthesis and regulation^2^. A recent study has suggested that the local clock is a key driver of steroid hormone production in *Drosophila* PG^3^. An ultrastructural study in *Drosophila* has suggested that the PG cells may be performing other roles beyond endocrine synthesis^4^. Previous studies on genes expressed in the insect PG were initially focused on characterization of individual genes, particularly those involved in steroid hormone biosynthesis and regulation as well as circadian clock mechanism^5^. Although the ecdysteroid in numerous insects had been studied for decades, yet advances in understanding this important organ at the molecular level remains largely unknown. At the start of this work, the basic genomic information is lacking, although a proteomic approach had been utilized to investigate the feature of *B. mori* PG^6^. Very recently, two research teams just released their results for *B. mori* PG by transcriptomic approach^7,8^. In the two studies, cell membrane receptors and signalling pathways and new players in ecdysteroidogenesis of *B. mori* PG were focused on. However, a better understanding of insect PG requires an expansion of the taxon samplings.

In this paper, we used Illumina sequencing of cDNAs from the larval PGs of two economically important silkmoth species, *B. mori* and *A. pernyi* to characterize their common expressed genes that present the basic factors necessary for the function of the PG. The former is the model insect for the order Lepidoptera, and has economically important values for silk production^9^. The latter is one of the most well-known wild silkmoths used for silk production and as a source of insect food for human consumption. *A. pernyi* is also a model system in study of insect diapause and endocrine regulation due to its pupal-diapause and large size^10^. We generated over 24 million high-quality sequence reads that assembled into about 50,000 transcripts. The transcriptome data will contribute to knowledge of the molecular components in the PG of insects. By searching against the transcriptome data, we have identified almost all the steroid hormone biosynthesis genes and the prothoracicitropic hormone receptor gene (*torso*), several juvenile hormone related genes, two ecdysone receptor genes and a set of clock genes in the PGs of two silkworms. We also identified more than 10 chemosensory protein genes (CSPs) in the two PGs. To our knowledge, this is the first comparative view of the genes transcribed in this unique organ.

## Results

### Illumina sequencing and transcriptome assembly

Transcriptomic sequence data were generated using two PG cDNA libraries from *B. mori* and *A. pernyi*, and Illumina HiSeq 2500 technology. The PGs were collected from a pool of ~30 silkwom larvae of fifth instar. We acquired 28,159,208 and 24,408,498 clean reads from the PG transcriptomes of *A. pernyi* and *B. mori*, respectively, after removing adapters, ambiguous nucleotides and low quality sequences. For *B. mori*, 5.73 Gbp of clean sequence data was generated with a Q30 value of 91.35% and a GC content of 45.75%. The assembly resulted in 49,287 transcripts longer than 200 bp, which were further assembled into 32,302 unigenes, with an N50 of 1,510 and mean length of 798 bp. For *A. pernyi*, we ultimately obtained 6.60 Gbp of clean sequence data with a Q30 value of 91.73% and a GC content of 44.55%. The trinity assembly of the clean sequence data of *A. pernyi* resulted in 64,301 transcripts longer than 200 bp, which were further assembled into 44,067 unigenes, with an N50 of 865 and mean length of 549 bp. For each species, at least 6100 unigenes are >1000 bp and 12,500 >500 bp in length (Additional file: Fig. S1). An overview of the sequencing and assembly process is presented in Additional file: Table S1. The sequence data for *B. mori* and *A. pernyi* PGs have been deposited in the NCBI Sequence Read Archive (SRA) database under accessions SRX2434881 and SRX2434884, respectively, and the assembled sequences have been deposited in Transcriptome Shotgun Assembly (TSA) database under accessions GFCX00000000 and GFCY00000000 associated with Bioproject PRJNA357974 and PRJNA357975 for *A. pernyi* and *B. mori* PGs, respectively.

### Functional annotation revealed an overall similarity in gene profile between the two PGs

For functional annotation, we searched all unigene sequences using Blastx tool against NCBI non-redundant protein database (Nr), with a cut-off E-value of 10^−5^. Using this approach, 15,187 (47.02% of all distinct sequences) and 19,035 (43.19%) unigenes for *B. mori* and *A. pernyi* returned a Blast hit in the Nr database, respectively, 8,791 and 10,283 unigenes had specific matches in the Swiss-Prot database, and 9,188 and 10,974 unigenes had matches in the Pfam database. Totally, 23,157 (71.69%) and 22,402 (50.84%) unigenes were annotated in at least one database for *B. mori* and *A. pernyi* PGs (Additional file: Table S1).

**Figure 1:**
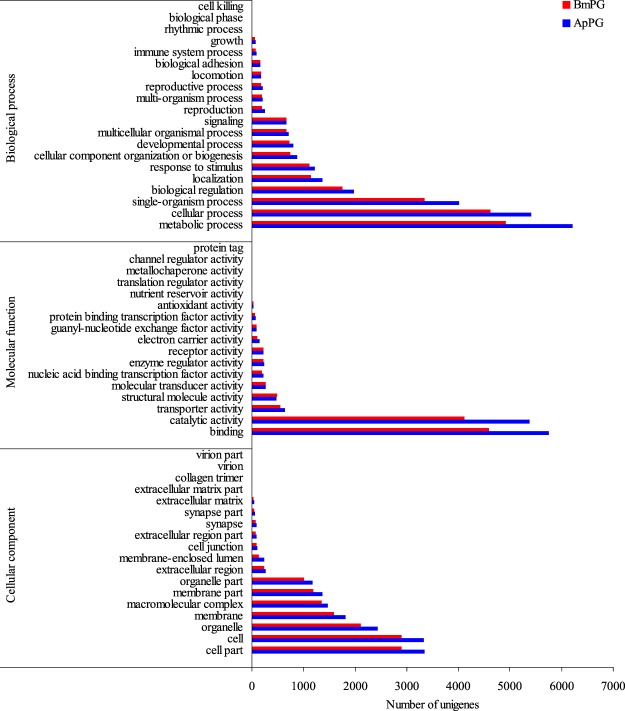
Gene ontology (GO) analysis of the PG transcriptomes of *A. pernyi* and *B. mori* unigenes according to their involvement in biological process, cellular component and molecular function.

Firstly, we used Blast2GO^11^ to perform functional annotation for the PG transcriptome via gene ontology (Fig. 1). For *B. mori*, a total of 8,557 unigenes were assigned GO terms, including 6,208 with hits at the Biological Process level, 4,065 at the Cellular Component level and 7,337 at the Molecular Function level. For *A. pernyi*, 10,621 unigenes were assigned GO terms, including 7,382 at the Biological Process level, 4,662 at the Cellular Component level and 9,170 at the Molecular Function level. Within the Biological Process GO categories, the most abundant transcripts for the two PGs were assigned to “metabolic process” (6,218 in *A. pernyi* and 4,926 in *B. mori*), “cellular process” (5,406 in *A. pernyi* and 4,625 in *B. mori*), and “single-organism process” (4,006 in *A. pernyi* and 3,342 in *B. mori*). “Cell part” (3,337 in *A. pernyi* and 2,905 in *B. mori*), “cell” (3,323 in *A. pernyi* and 2,894 in *B. mori*), and “organelle” (2,439 in *A. pernyi* and 2,103 in *B. mori*) were the most represented GO terms for Cellular Components in both PGs. For Molecular Function, “binding” (5,751 in *A. pernyi* and 4,587 in *B. mori*), “catalytic activity” (5,385 in *A. pernyi* and 4,111 in *B. mori*) were the most prevalent in the two PGs. Overall, the percentage of Blastx hits distributed among GO categories was highly similar in both PGs.

Secondly, unigenes of the two PGs were characterized by KOG classification to enable conceptualization of its transcripts into potential functional groups. In total, 10,404 and 12,837 unigenes for *B. mori* and *A. pernyi* were annotated to 25 KOG categories, respectively (Additional file: Fig. S2). The numbers of each KOG category were similar between *A. pernyi* and *B. mori* PG transcriptome. The KOG classification indicated that except ‘general function prediction’, genes involved in “signal transduction mechanisms” (1,373 unigenes in *B. mori* and 1,454 in *A. pernyi*), “post translational modification, protein turnover, chaperones”(790 unigenes in *B. mori* and 1,052 in *A. pernyi*), and “translation, ribosome structure and biogenesis” (638 unigenes in *B. mori* and 618 in *A. pernyi*) were most abundant.

**Figure 2:**
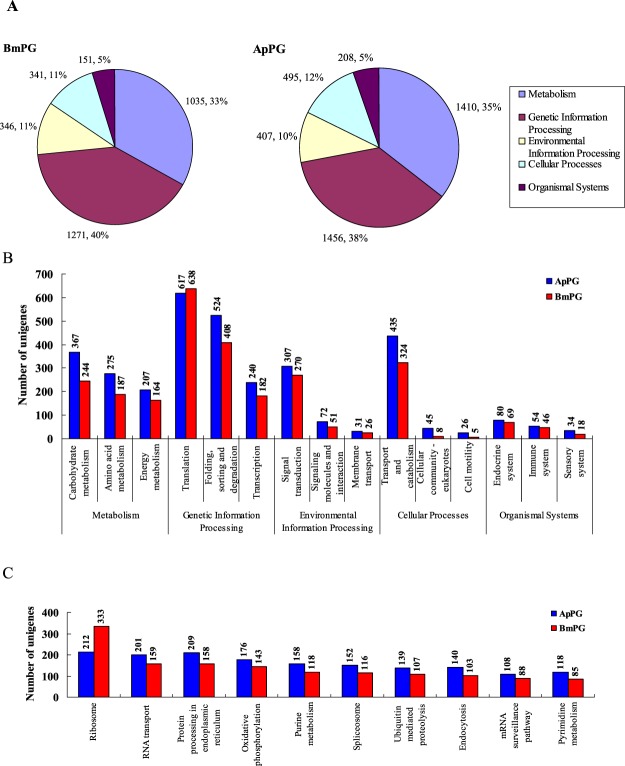
KEGG pathway analysis of *A. pernyi* and *B. mori* PG trancriptomes. The figure A depicts the distributions of total assignments for both *A. pernyi* and *B. mori* Blastx assigned unigenes among global KEGG pathways, and the proportions of the five global KEGG pathways were highly similar between the two species, as depicted by the percentage values. Figure **B** and **C** show comparison of *A. pernyi* and *B. mori* PG unigenes associated with selected second- and third-tier KEGG hierarchical pathways, respectively. The top ten third-tier KEGG pathways with the highest number of unigenes for two species were shown.

Lastly, KEGG orthology (KO) assignments^12^ were comparable between the two PGs. The KO assignment analysis resulted in annotation of 179 and 195 pathways, corresponding to 4,665 and 5,820 unigenes in *B. mori* and *A. pernyi*, respectively, and the global KO assignments showed similar trends in both PGs (Fig. 2A). The second- and third-tier pathways (Fig. 2B,C) also indicated a common expressed-gene profile between the two PGs. In the KEGG second-tier pathway hierarchy, “translation”, “folding, sorting and degration” and “transport and catabolism” pathways ranked first to third in the two PGs, respectively.

### Expression of ecdysteroidgenesis genes in two PGs

Insect PG is one of the most important endocrine organs that synthesizes and releases ecdysteroid hormone. As expected, almost all known steroid hormone biosynthesis genes (*neverland*, *spook*, *phantom*, *disembodied*, *shadow*, *shroud*, *Cyp6u1*) and the prothoracicitropic hormone receptor gene (*torso*) are well represented in two PGs (Table 1). Local blast search against the transcriptome data of two silkmoths indicated that no expression of the *shade* gene is detected, which is consistent with ecdysone being activated to 20-Hydroxyecdysone (20-E) in peripheral tissues and not the PG^13^. Note that *B. mori neverland* was obtained by searching Blastn against a recent released PG transcriptome data (SRX1142589)^8^. The *shroud* gene in *B. mori* that encodes a short-chain dehyrogenase/reductase involving in the ecdysteroid biosynthesis pathway is PG- and ovary-specific^14^; we got the sequences of the *shroud* gene in both PG transcriptome data. A recent work in *D. melanogaster* has provided strong evidence that *Cyp6u1* may have a role in ecdysteroidogenesis, possibly in the Black Box^15^; the homologues were also identified in two silkworm PGs. We also identified two genes encoding ecdysone receptor B and ultraspiracle 2 that are expressed in the PG transcriptomes of two silkworms. These genes were confirmed by comparing them with known genes from *D. melanogaster* using phylogenetic analysis (Additional file: Fig. S3). The RPKM values of these genes were also evaluated, and *spook* was the most abundant. The high expression of *spook* in two silkworm PGs was not consistent with the low expression in *D. melanogaster*^15^.

**Table 1:** Genes of interest present in two PGs.

Gene	*A. pernyi* PG				*B. mori* PG	
	Unigene ID	BLASTx annotation	Identity (%)	RPKM	Unigene ID	FPKM
**Steroid hormone related genes**						
*Neverland*	ApPG.21305	NP_001037626 [*Bombyx mori*]	64	147.04	—	—
*Spook*	ApPG.15723	BAH47267 [*Bombyx mori*]	73	659.32	BmPG.4736	1840.90
*Phantom*	ApPG.17763	BAM73853 [*Bombyx mori*]	80	254.14	BmPG.6242	331.55
*Disembodied*	ApPG.18472	BAM73849 [*Bombyx mori*]	71	162.28	BmPG.6349	149.33
*Shadow*	ApPG.19814	BAM73862 [*Bombyx mori*]	62	187.1	BmPG.6200	525.60
*Shroud*	ApPG.20406	NP_001171333 [*Bombyx mori*]	67	262.63	BmPG.6198	423.82
*Cyp6u1*	ApPG.21208	NP_001296520 [*Bombyx mori*]	79	7	BmPG.8514	3.46
*Torso-like*	ApPG.20311	XP_012546780 [*Bombyx mori*]	74	22.87	BmPG.26303	2.75
*Ecdysone receptor B*	ApPG.17239	ABS87644 [*Bombyx mori*]	83	2.6	BmPG.3587	12.87
*Ultraspiracle*	ApPG.20814	NP_001037470 [*Bombyx mori*]	86	3.89	BmPG.2862	42.55
**Juvenile hormone related genes**						
*Farnesyl diphosphate phosphatase*	ApPG.18455	NP_001040333 [*Bombyx mori*]	67	2.77	BmPG.4947	5.78
*Aldehyde dehydrogenase (NAD+)*	ApPG.20532	XP_004931115 [*Bombyx mori*]	75	21.38	BmPG.3314	50.06
*Juvenile hormone acid methyltransferase*	ApPG.39533	AEV45620 [*Bombyx mori*]	39	0.63	BmPG.25316	0.70
*Juvenile hormone epoxide hydrolase*	ApPG.21667	BAF81491 [*Bombyx mori*]	62	95.86	BmPG.6690	44.54
*Juvenile hormone esterase*	ApPG.8009	AAR37335 [*Bombyx mori*]	53	1.2	BmPG.28447	2.05
*Cytosolic juvenile hormone binding protein*	ApPG.16002	NP_001037668 [*Bombyx mori*]	86	159.49	BmPG.6419	85.15
*Juvenile hormone binding protein*	ApPG.12156	BAH97100 [*Bombyx mori*]	54	22.63	BmPG.7107	29.05
*Juvenile hormone esterase binding protein*	ApPG.17810	ABI23690 [*Bombyx mori*]	79	37.81	BmPG.6929	23.16
*Hexamerin*	ApPG.19575	XP_004931806 [*Bombyx mori*]	67	19.35	BmPG.23350	0.30
*Broad*	ApPG.15073	XP_004931900 [*Bombyx mori*]	77	2.66	BmPG.3380	9.11
*Allatostatin receptor*	ApPG.9900	NP_001127736 [*Bombyx mori*]	84	57.34	BmPG.1024	2.13
**Circadian clock related genes**						
*Cryptochrome 1*	ApPG.13030	NP_001182628 [*Bombyx mori*]	85	4.8	BmPG.5659	8.61
*Cryptochrome 2*	ApPG.15292	NP_001182627 [*Bombyx mori*]	76	1.98	BmPG.3117	11.64
*Period*	ApPG.21754	NP_001036975 [*Bombyx mori*]	55	45.41	BmPG.2896	7.70
*Timeless*	ApPG.19643	NP_001037622 [*Bombyx mori*]	68	19.84	BmPG.4674	80.17
*Clock*	ApPG.17196	AAR14936 [*Antheraea pernyi*]	99	0.49	BmPG.4602	3.25
*Cycle*	ApPG.4088	AR14937 [*Anheraea pernyi*]	100	1.33	BmPG.322	3.41
*Shaggy*	ApPG.17018	C42322 [*Helicoverpa armigera*]	98	5.45	BmPG.32148	34.96
*Double-time*	ApPG.720	NP_001037285 [*Bombyx mori*]	94	1.55	BmPG.4533	19.90
*Vrille*	ApPG.15026	AAS92609 [*Antheraea pernyi*]	100	53.15	BmPG.23823	3.65
*PAR-domain protein 1 ε*	ApPG.15874	AGR44476 [*Ostrinia furnacalis*]	86	28.99	BmPG.2118	82.12
*Slimb*	ApPG.3311	ABV22506 [*Danaus plexippus*]	98	1.81	BmPG.263	13.26
*Casein kinase II alpha*	ApPG.17025	NP_001036956 [*Bombyx mori*]	98	26.27	BmPG.407	64.36
*Casein kinase II beta*	ApPG.17504	NP_001036989 [*Bombyx mori*]	92	73.51	BmPG.1530	88.65

### Genes related to juvenile hormone regulation in PGs

**Figure 3:**
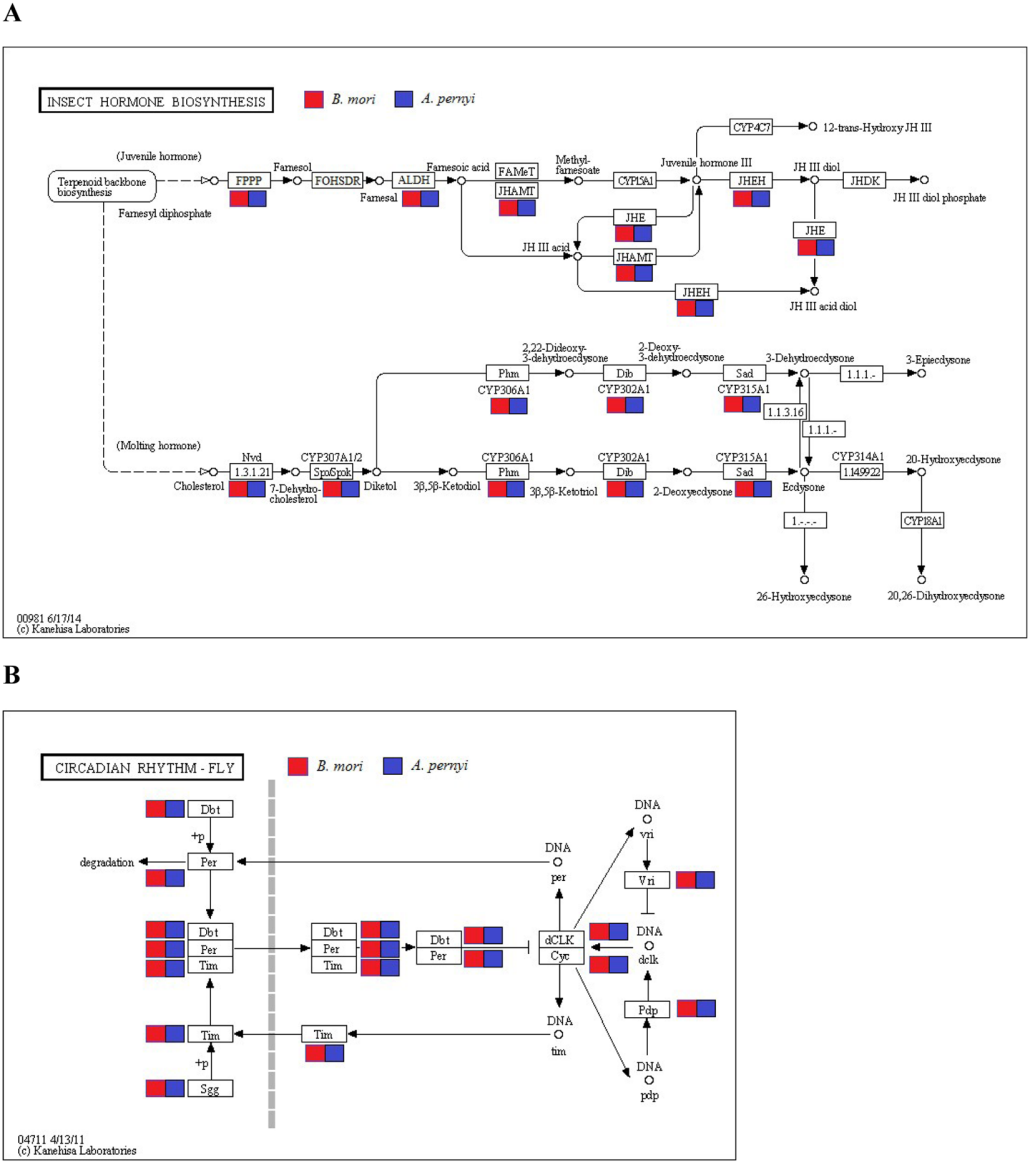
Identification genes assigned to the Insect Hormone Biosynthesis and Circadian Rhythm pathways^12^ in *A. pernyi* and *B. mori* PG transcriptomes. The Insect Hormone Biosynthesis (**A**) and Circadian Rhythm (**B**) pathway components/genes were represented in both species. Copyright permission of KEGG pathway map images has been obtained from Kanehisa Laboratories with ref: 170270.

Like ecdysteroids, juvenile hormone (JH) is also an important endocrine hormone that determines the nature of molt, and the CA has been considered as the only source of JH in insects^16^. In this study, we identified 11 juvenile hormone related genes that are represented in the PG transcriptome data from two silkworms (Table 1 and Fig. 3A), including *Farnesyl diphosphate phosphatase* (*FPPP*), *Aldehyde dehydrogenase* (*ALDH*), *juvenile hormone acid methyltransferase* (*JHAMT*), *juvenile hormone epoxide hydrolase* (*JHEH*), *juvenile hormone esterase* (*JHE*), *cytosolic juvenile hormone-binding protein* (*cJHBP*), *juvenile hormone binding protein* (*JHBP*), *juvenile hormone esterase binding protein* (*JHEBP*), *hexamerin*, *broad* and *allatostatin receptor*. However, we did not get the sequences of the genes such as *NADP*+ -*dependent farnesol dehydrogenase* (*FOHSDR*), *methyl farnesoate epoxidase/farnesoate epoxidase* (*CYP15A1*), *juvenile hormone diol kinase* (*JHDK*) and *sesquiterpenoid omega-hydroxylase* (*CYP4C7*). All these juvenile hormone related genes were confirmed by phylogenetic analysis (Additional file: Fig. S4).

### Genes involved in circadian clock in PGs

Physiological experiments by transplantation have evidenced the presence of a local clock in PG of the saturniid moth *Samia* *cynthia* *ricini*^17^. A local clock has also been suggested to play a key driver of steroid hormone production in *Drosophila* PG, and all of genes related to circadian clock were well represented in the ring gland transcriptome^15^. In the present study, a set of genes important for circadian clock mechanism were identified in the PGs of two silkmoths (Table 1 and Fig. 3B), including *cryptochrome 1*, *cryptochrome 2*, *cycle*, *clock*, *vrille*, *timeless*, *slimb*, *period*, *double time*, *shaggy*, *PAR-domain protein 1ε*, *casein kinase 2 alpha*, *casein kinase 2 beta*, which further confirmed the presence of a local clock in the PG, although the expression level of each gene was low. Among these genes, the expression of *clock* was the least and *casein kinase 2 beta* was the most abundant. We confirmed these clock related genes by phylogenetic analysis (Additional file: Fig. S5).

### Chemosensory protein genes in PGs

**Figure 4:**
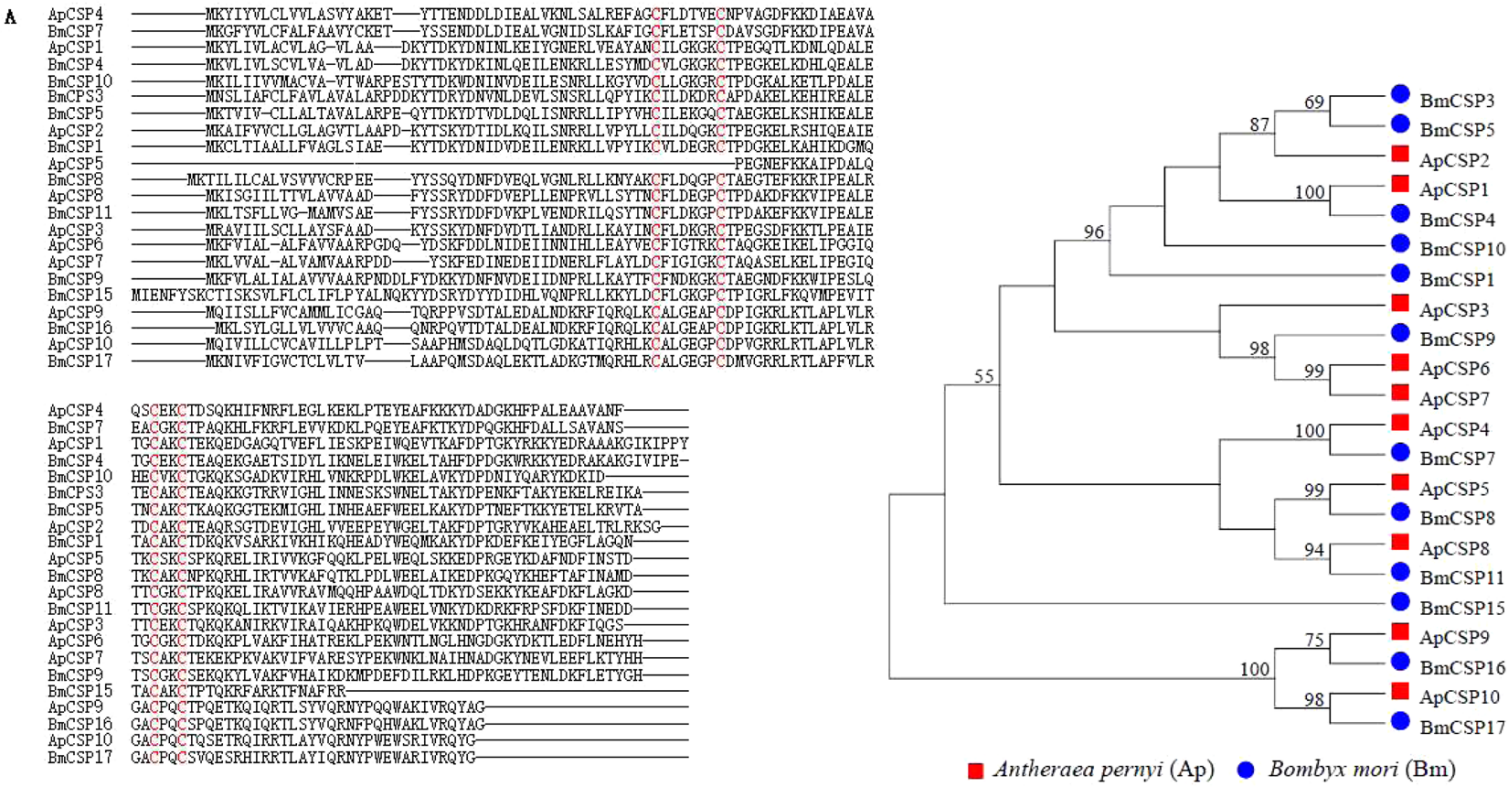
Comparision of chemosensory proteins (CSPs) identified in the PG transcriptomes of two silkworms, *A. pernyi* and *B. mori*. (**A**) Sequence alignment. (**B**) Neighbor-joining tree. Bootstrap values are calculated by 1000 replicates and those larger than 50% are marked on the nodes. The accession numbers of sequences are available in Additional file.

The genome of *B. mori* harbours 16 CSP genes (BmCSP1-16)^18^; we identified 11 of them and one novel BmCSP17 in the PG transcriptome data (Table 2; Fig. 4A). BmCSP17 exhibited a highest sequence identity with BmCSP16, with a value of 61%. In *A. pernyi* PG, we also identified 10 CSPs. These 10 ApCSPs demonstrated 45–77% amino acid sequence identities with corresponding BmCSPs. Sequence comparison between *A. pernyi* and *B. mori* showed that there are six pairs of homologous (Fig. 4B).

**Table 2:** Chemosensory protein genes present in two PGs.

Gene Name	Unigene ID	ORF (aa)	Complete ORF	Blastx annotation	Blastx acc. no.	Blastx species	Identity (%)	FPKM
***A. pernyi*** **PG**								
ApCSP1	ApPG_15810	128	Yes	chemosensory protein 1	AAV34688	*Bombyx mori*	68	278.20
ApCSP2	ApPG_8092	128	Yes	chemosensory protein 5	NP_001037062	*Bombyx mori*	60	34.10
ApCSP3	ApPG_18212	121	Yes	chemosensory protein 6	NP_001037400	*Bombyx mori*	71	17.37
ApCSP4	ApPG_12457	122	Yes	chemosensory protein 7	NP_001037068	*Bombyx mori*	66	500.23
ApCSP5	ApPG_30646	69	Not	chemosensory protein 8	NP_001037067	*Bombyx mori*	68	1.16
ApCSP6	ApPG_5694	124	Yes	chemosensory protein 9	NP_001037066	*Bombyx mori*	49	72.76
ApCSP7	ApPG_10897	122	Yes	chemosensory protein 9	NP_001037066	*Bombyx mori*	45	207.57
ApCSP8	ApPG_15471	122	Yes	chemosensory protein 11	NP_001091779	*Bombyx mori*	64	18.55
ApCSP9	ApPG_12919	107	Yes	chemosensory protein 16	NP_001091782	*Bombyx mori*	77	2.54
ApCSP10	ApPG_18214	106	Yes	chemosensory protein 16	NP_001091782	*Bombyx mori*	65	1.37
***B. mori*** **PG**								
BmCSP1	BmPG_8551	123	Yes	chemosensory protein 1	NP_001037065	*Bombyx mori*	100	3.81
BmCPS3	BmPG_7145	127	Yes	chemosensory protein 3	NP_001037063	*Bombyx mori*	100	29.68
BmCSP4	BmPG_7684	127	Yes	chemosensory protein 4	NP_001037052	*Bombyx mori*	100	7.85
BmCSP5	BmPG_26685	125	Yes	chemosensory protein 5	NP_001037062	*Bombyx mori*	100	52.83
BmCSP7	BmPG_7391	122	Yes	chemosensory protein 7	NP_001037068	*Bombyx mori*	100	14.46
BmCSP8	BmPG_28989	124	Yes	chemosensory protein 8	NP_001037067	*Bombyx mori*	100	2.36
BmCSP9	BmPG_7078	127	Yes	chemosensory protein 9	NP_001037066	*Bombyx mori*	100	39.41
BmCSP10	BmPG_11310	122	Yes	chemosensory protein 10	ABH88203	*Bombyx mori*	100	1.27
BmCSP11	BmPG_11636	121	Yes	chemosensory protein 11	NP_001091779	*Bombyx mori*	100	1.35
BmCSP15	BmPG_11195	103	Not	chemosensory protein 15	NP_001091781	*Bombyx mori*	100	2.10
BmCSP16	BmPG_7447	106	Yes	chemosensory protein 16	NP_001091782	*Bombyx mori*	100	12.32
BmCSP17	BmPG_26561	104	Yes	chemosensory protein 16	NP_001091782	*Bombyx mori*	61	14.88

### Changes in expression level of juvenile hormone regulation and circadian clock related genes in PG during development in whole fifth larval instar

**Figure 5:**
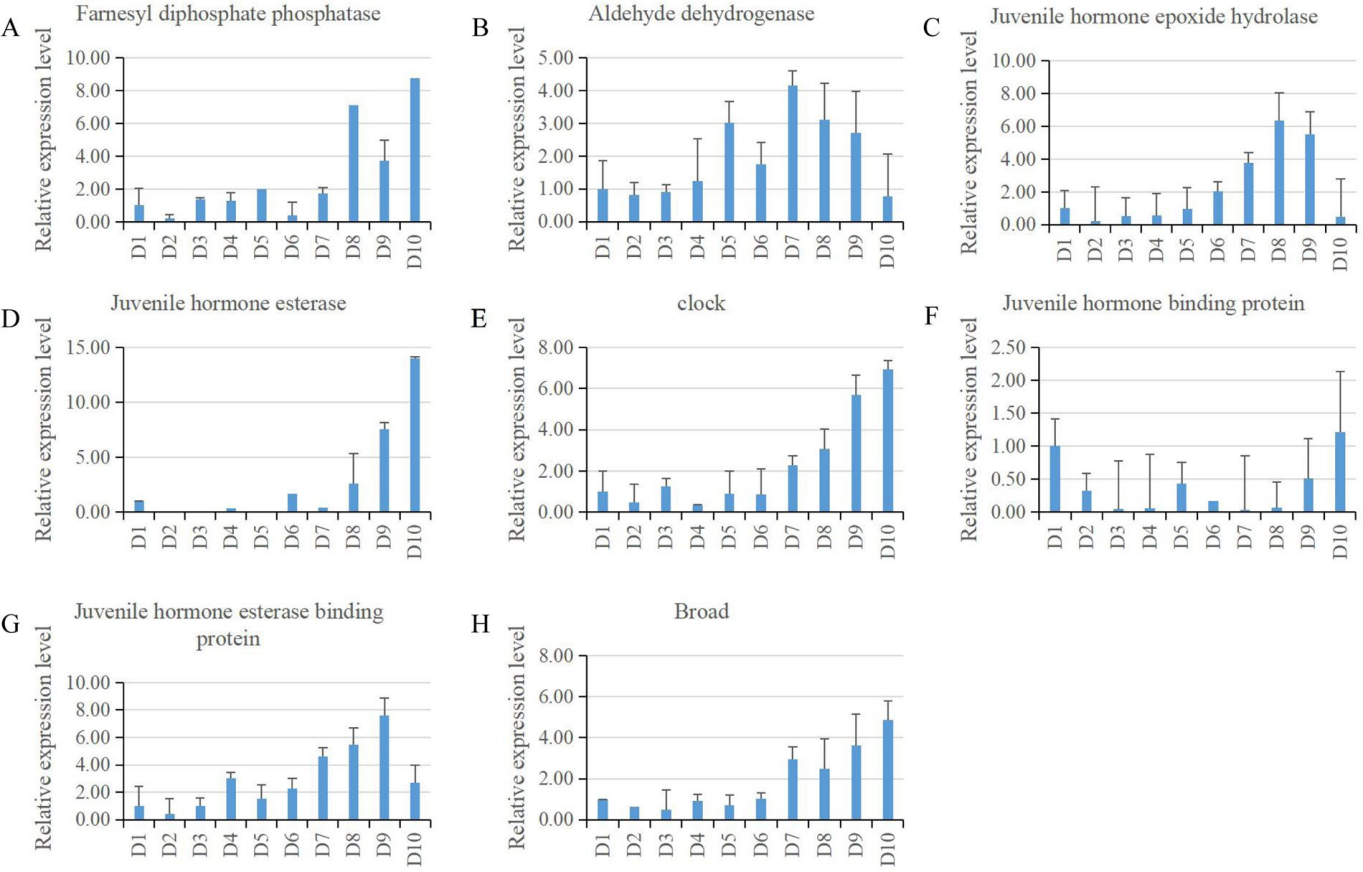
qRT-PCR results of expression of the JH related genes. The expression patterns of 8 genes related to JH in *B. mori* PG during development in whole fifth larval instar. The relative expression levels were normalized to the *Bmrp49* levels. The values are the mean ± SEM (n = 3) of three repeat experiments using qRT-PCR.

We further used quantitative real time -PCR (qRT-PCR) to validate and investigate the changes in expression level of 11 juvenile hormone regulation and 13 circadian clock related genes in *B. mori* PG during development in whole fifth larval instar. The qRT-PCR results confirmed the expression of 8 genes out of these 11 juvenile hormone regulation related genes (Fig. 5). Among them, 4 genes (*ALDH*, *JHEH*, *cJHBP* and *JHEBP*) exhibited a similar expression pattern with a trend of rise first then fall; *JHE* and *Broad* showed a gradual rise tendency with the highest expression at day 10; *FPPP* generated a fluctuation change with a gradual rise utill day 8, then decreased and increased again; *JHBP* remained a very low expression level, but revealed a distinct expression change with a trend of fall first then rise on the final day.

**Figure 6:**
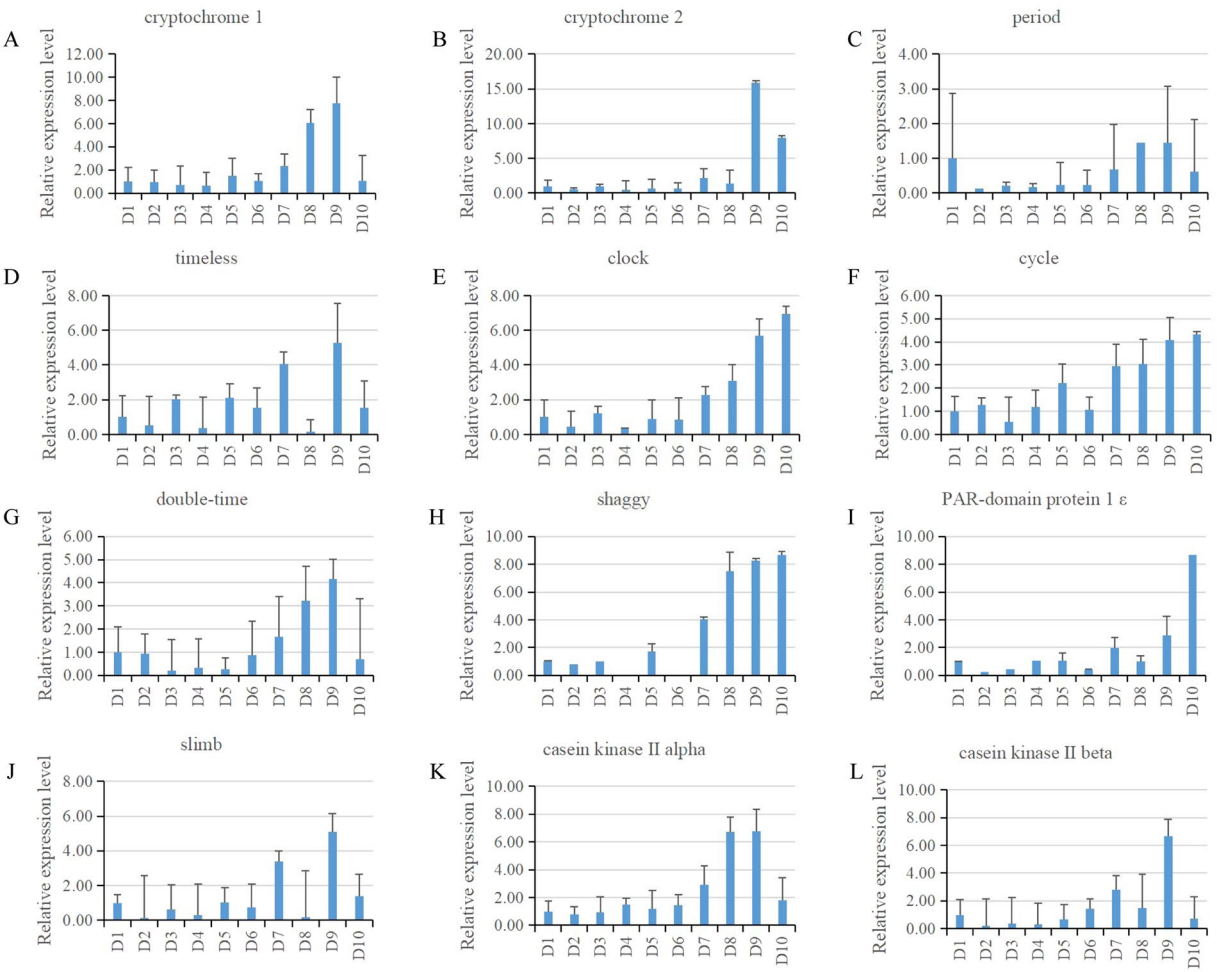
qRT-PCR results of expression of the clock genes. The expression patterns of 12 genes related to clock in *B. mori* PG during development in whole fifth larval instar. The relative expression levels were normalized to the *Bmrp49* levels. The values are the mean ± SEM (n = 3) of three repeat experiments using qRT-PCR.

Out of 13 clock related genes identified in two PGs, 12 were indeed expressed in the larval PG of *B. mori* by qRT-PCR detection method (Fig. 6). Among them, 7 genes (*cryptochrome 1*, *cryptochrome 2*, *timeless*, *double time*, *slimb*, *casein kinase 2 alpha* and *casein kinase 2 beta*,) presented a similar expression pattern with a trend of rise first then fall; 4 genes (*cycle*, *clock*, *shaggy* and *PAR-domain protein 1ε*) showed a gradual rise tendency with the highest expression at day 10; *period* gave a distinct expression change with a trend of fall first then rise.

## Discussion

Despite PG is an important endocrine organ in insects, little information on it at the global level is already known, especially the features beyond endocrine synthesis. The present study, for the first time, use a comparative view to analyze the gene network in the PG of two silkmoths, *B. mori* and *A. pernyi*, by taking a transcriptomic approach. Since the complete genomes can be available, their annotation rates reache 71.7% for *B. mori* PG and 57.8% for *D. melanogaster* ring glands transcriptomes^15^, respectively. The annotation rate of *A. pernyi* (50.8%) is lower than that of *B. mori*, but it is comparable to those of other lepidopteran insects such as *Helicoverpa armigera* (50.8%), *H. assulta* (54.0%), *Spodoptera frugiperda* (51.1%) and *Athetis lepigone* (41.5%)^19–21^. Comparison of *A. pernyi* and *B. mori* PG GO terms and KOG categories revealed that both transcriptomes have similar gene expression profiles. The high similarity of percentage of KO assignments across global KEGG pathways between *A. pernyi* and *B. mori* also indicates that the PG transcriptomes share a common expressed-gene profile. This validates the application of RNA-Seq technology for identification of orthologs in non-model organism. Thus, the PG transcriptomes are useful resources to identify gene networks controlling PG ecdysteroidogenesis and understand the novel features of PG beyond endocrine synthesis.

20-E exerts its effect through binding to its receptor, a heterodimer of ecdysone receptor B (EcR) and ultraspiracle (USP). Two genes encoding EcRB and USP2 are expressed in the PG transcriptomes of two silkworms, providing further evidence that 20-E is involved in feedback loops in the PG^22,23^.

Recent studies have shown that JH plays an important role in regulating PG activity^24,25^; however, the underlying molecular mechanism is severely limited. Decophering the mechanism underlying cross-talk between JH and ecdysone is key to understanding the control of insect growth and development. JH is degraded predominantly by hydrolytic enzymes, JHE and JHEH^25^. JHEBP might function in JHE transportation and degradation when the JH III titer is high^26^. JHBP serves as a carrier supplying JH to the target tissues. Hexamerin modulates JH availability^27^. Broad is a transcription factor that mediates the effects of ecdysone and juvenile hormone. Our qRT-PCR results confirmed the expression of 8 genes (*ALDH*, *JHEH*, *cJHBP*, *JHEBP*, *JHE*, *Broad*, *FPPP* and *JHBP*) in *B. mori* PG. No detectable expression of 3 genes (*JHAMT*, *hexamerin* and *allatostatin receptor*) by qRT-PCR method was consistent with the RNA-seq results by a very low FPKM value. It has already been shown that in *B. mori JHAMT* was specificlly expressed in the CA, and trace amounts in the PG^28^. The presence of 8 JH related genes in the PG suggested that through these JH related genes JH cross talk with ecdysteroid hormone. To understand the function of these JH related genes in the PG, it is necessary to investigate whether they are functionally important by performing qRT-PCR with CA and other tissues (for example, fat body) in the future work.

Circadian clock is an important regulator of behavior and physiology in insects. In addition to the central clock in brain, peripheral clocks reside in various organs and tissues^29^. For examples, the peripheral clocks in the Malpighian tubules, antenna, fat body of *Drosophila* have been well studied. These peripheral clocks are independent of the central clock, and the oscillatory machinery and entrainment mechanism of peripheral clocks vary between different tissues and organs. In *Drosophila*, the eclosion rhythm is set by a local clock residing in the PG that is a key driver of steroid hormone production^3^. Compared with *Drosophila*, very little attention has been paid to the peripheral clocks in lepidopteran insects^17^. In the PG transcriptomes of two silkmoths, we also identified 13 genes important for circadian clock mechanism and qRT-PCR results confirmed the expression of 12 genes in the *B. mori* PG, thus extending our understanding of the local peripheral clock residing in the PG of lepidopteran insects^17^ that may also involve in steroid hormone production. To address this issue, we will investigate the fluctuations in expression levels of circadian clock related genes of night and day PG samples in the future.

Insect CSPs are small soluble acidic proteins that are believed to be involved in chemical communication, including perception, identification, transport and transduction of semiochemicals from environment (olfaction, taste and others) and may be associated with regulation of circadian rhythms and maturation of tissue or appendage^30^. CSPs are expressed not only in insect sensory organs, but also in other tissues that lack gustatory and olfactory neurons^31^. These non-chemosensory tissues included cuticle, legs, labial palp, pheromone gland, tarsi, proboscis, wings, testes, ovaries, compound eyes, hemolymph and ejaculatory^30^. The PG transcriptome data of two silkworms offered us an opportunity to investigate gene expression profiles of CSP genes on a large-scale in PGs. Our study evidenced the presence of 12 and 10 CSPs in *B. mori* and *A. pernyi* PG, suggesting that there have a link between CSPs and ecdysteroids^32^.

Earlier study in *B. mori* demonstrated that KK-42 can reduce the incidence of embryonic diapause when administered to the mother during her final larval instar^33^. By contrast, our recent results showed that KK-42 can delay termination of the pupal diapauses in *A. pernyi* and *Helicoverpa zea*, and boost pupal diapause incidence when administered to larvae of *H. zea*^34^. The mechanism is that KK-42 appears to act by inhibiting ecdysteroid biosynthesis within the PG, without killing the PG cells^35^. Previous studies suggested that a KK-42 binding protein might be a receptor of an endogenous signaling compound^36^; the expression of this gene could be detectable in many organs of *A. pernyi*^37^. However, we did not detect the expression of this gene in the two PGs, indicating that KK-42 inhibits ecdysteroid biosynthesis within the PG not by the KK-42 binding protein. How does KK-42 influence ecdysteroid biosynthesis within the PG? To address this issue, comparison of KK-42-treated and untreated PGs would provide valuable clues in the near future.

In conclusion, this present work provided a comparative analysis of the PG transcriptomes of two silkmoths, whose associated expressed-gene profile were highly similar. Our results uncovered the presence of at least 8 juvenile hormone related genes, 12 circadian clock genes, and 10 chemosensory protein genes in both PGs. This contribution to insect PG transcriptome data will extend our understanding of the function and regulation of this important organ.

## Materials and Methods

### Insect materials and samples collection

Samples were derived from strain *Shenhuang no. 2* of *A. pernyi* and *Dazao* of *B. mori* maintained at the Department of Sericulture, Shenyang Agricultural University in Shenyang. Larvae of *A. pernyi* strain were reared on oak trees in the field until the fifth larval stage, and then reared at room (25 °C, natural humidity) using oak branches with leaves. Larvae of *B. mori* were reared using mulberry leaves during the whole larval stage at 25 °C and 50–70% relative humidity in natural light. To generate samples, ~30 worms of *B. mori* and *A. pernyi* were collected in the third day and the fifth day of the fifth instar, respectively. The PGs were carefully removed from the worms in insect Ringer physiological saline buffer under dissecting microscope, then immediately placed in 500 μl TRIzol reagent (Beijing Sinogene, China) and stored at −80 °C. Frozen tissues in TRIzol were shipped to Biomarker Technologies in Beijing, China for RNA extraction, library preparation and DNA sequencing.

### RNA extraction and sequencing

Total RNAs were extracted from frozen PGs with TRIzol. RNA integrity and concentration were assessed with a NanoDrop ND-1000 spectrophotometer (Thermo Scientific, Wilmington, DE, USA) and an Agilent 2100 Bioanalyzer (Agilent Technologies, Santa Clara, CA, USA). Sequencing libraries were prepared using NEBNext Ultra RNA Library Prep Kit for Illimina (NEB, E7530) and NEBNext Multiplex Oligos for Illumina (NEB, E7500). Two RNA samples were sequenced on the Illumina HiSeq™ 2500 sequencing platform (paired-end, 125 bp reads).

### Transcriptome assembly

High-quality clean reads were obtained by removing the adaptor sequences, duplicated sequences, ambiguous reads (‘N’), and low-quality reads. For transcriptome data of *A. pernyi* that has no complete genomic data available, the clean reads were pooled for assembly using Trinity (http://trinityrnaseq.sourceforge.net/)^38^, and the related contigs were then clustered using the TGICL software^39^ to yield unigenes (without N) that cannot be extended on either end, and redundancies were removed to acquire non-redundant unigenes. For transcriptome data of *B. mori* that has complete genomic data available, the clean reads were mapped to genome using Tophat2 software^40^. Transcript expression levels were estimated with FPKM values (fragments per kilobase of exon per million fragments mapped) by the Cufflinks software^41^. N50 and mean lengths of the transcripts associated with each sample were calculated. The values for N50 length and mean length indicated high quality samples, sequences and assemblies for the PGs of two silkmoths.

### Annotation

The unigenes of the two silkmoths were compared against public databases, including NR (non-redundant), GO (gene ontology), KOG (eukaryotic ortholog groups), KEGG (Kyoto Encyclopedia of Genes and Genomes), Swiss-Prot and TrEMBL databases using BLASTx with an E-value cutoff at 10^−5^ to retrieve protein functional annotations with the highest sequence similarity. High-priority databases (followed by Nr, Swiss-Prot, and KEGG) were selected to determine the direction of the unigene sequences. The best aligning results were used to predict the coding region sequences from unigenes, and the coding sequences were translated into amino sequences using the standard codon table.

### Phylogenetic analysis

The accession numbers of sequences used for phylogenetic analysis are listed in Additional file: Table S2. Amino acid sequences were aligned with ClustalX 1.83^42^ and unrooted trees were constructed with MEGA6.0^43^ using the neighbour-joining method, with Poisson correction of distances and bootstrap replications set at 1000.

### Quantitative real time -PCR (qRT-PCR)

Two PGs per larva were used as one sample to extract the total RNA from 1-day-old fifth instar larva to 10-day-old fifth instar larva (matured silkworm) in the present study. All the extracted RNA (at least 2.6 μg/per fifth instar larva) using TRIpure (Beijing Aidlab biotechnologies Co. Ltd.) was converted into cDNA using the oligo(dT)_15_ primer with the PrimeScript RT reagent Kit with gDNA Eraser (Takara Biotechnology Dalian Co. Ltd.). The total volume of qRT-PCR reactions was 10 µl, containing 3.6 µl of TB Green Premix Ex Taq (TaKaRa), 0.4 µl of specific primers (10 µM), 1 µl of cDNA and 5 µl of ddH_2_O. qRT-PCR was performed with a BIO-RAD CFX Connect Real-Time System, and the conditions were as follows: 95 °C for 30 s followed by 40 cycles in 95 °C for 5 s and 60 °C for 30 s. Gene-specific primers used for qRT-PCR analysis are listed in Additional file: Table S3. The mRNA expression levels of the genes of interest were calculated with the 2^−ΔΔCt^ method and nomalized to the abundance of a house-keeping gene, ribosome protein 49 (*rp49*). The relative mRNA levels of each gene were represented as folds over the expression levels of *rp49*.

## Supplementary information

Transcriptomic Analysis of the Prothoracic Gland from Two Lepidopteran Insects, Domesticated Silkmoth Bombyx mori and Wild Silkmoth Antheraea pernyi

## References

[1] Yamanaka N, Rewitz KF, O’Connor MB (2013). Ecdysone control of developmental transitions: lessons from *Drosophila*. Annu. Rev. Entomol..

[2] Ou Q (2016). The insect prothoracic gland as a model for steroid hormone biosynthesis and regulation. Cell Rep..

[3] Di Cara F, King-Jones K (2016). The circadian clock is a key driver of steroid hormone production in *Drosophila*. Curr. Biol..

[4] Dai JD, Gilbert LI (1991). Metamorphosis of the corpus allatum and degeneration of the prothoracic glands during the larval-pupal-adult transformation of *Drosophila melanogaster*: a cytophysiological analysis of the ring gland. Dev. Biol..

[5] Morioka E, Matsumoto A, Ikeda M (2012). Neuronal influence on peripheral circadian oscillators in pupal *Drosophila* prothoracic glands. Nat. Commun..

[6] Li JY (2009). Proteomic and bioinformatic analysis on endocrine organs of domesticated silkworm, *Bombyx mori* L. for a comprehensive understanding of their roles and relations. J. Proteome Res..

[7] Moulos P, Samiotaki M, Panayotou G, Dedos SG (2016). Combinatory annotation of cell membrane receptors and signalling pathways of *Bombyx mori* prothoracic glands. Sci. Data.

[8] Nakaoka T (2017). Deep sequencing of the prothoracic gland transcriptome reveals new players in insect ecdysteroidogenesis. PLoS One.

[9] Xia Q (2009). Complete resequencing of 40 genomes reveals domestication events and genes in silkworm (Bombyx). Science.

[10] Wei ZJ (2008). Characters and expression of the gene encoding DH, PBAN and other FXPRLamide family neuropeptides in *Antheraea pernyi*. J. Appl. Entomol..

[11] Conesa A (2005). Blast2GO: a universal tool for annotation, visualization and analysis in functional genomics research. Bioinformatics.

[12] Kanehisa FM, Tanabe M, Sato Y, Morishima K (2017). KEGG: new perspectives on genomes, pathways, diseases and drugs. Nucleic Acids Res..

[13] Petryk A (2003). Shade is the *Drosophila* P450 enzyme that mediates the hydroxylation of ecdysone to the steroid insect molting hormone 20-hydroxyecdysone. Proc. Natl. Acad. Sci. USA.

[14] Niwa R (2010). Non-molting glossy/shroud encodes a short-chain dehydrogenase/reductase that functions in the ‘Black Box’ of the ecdysteroid biosynthesis pathway. Development.

[15] Christesen D (2017). Transcriptome analysis of *Drosophila melanogaster* third instar larval ring glands points to novel functions and uncovers a cytochrome p450 required for development. G3-Genes Genom. Genet..

[16] Sullivan JP, Jassim O, Fahrbach SE (2000). Juvenile hormone paces behavioral development in the adult worker honey bee. Horm. Behav..

[17] Mizoguchi A, Ishizaki H (1982). Prothoracic glands of the saturniid moth *Samia cynthia ricini* possess a circadian clock controlling gut purge timing. Proc. Natl. Acad. Sci. USA.

[18] Gong DP, Zhang HJ, Zhao P, Xia QY, Xiang ZH (2009). The odorant binding protein gene family from the genome of silkworm, *Bombyx mori*. BMC Genomics.

[19] Zhang J (2015). Antennal transcriptome analysis and comparison of chemosensory gene families in two closely related noctuidae moths, *Helicoverpa armigera* and *H. assulta*. PLoS One.

[20] do Nascimento AR, Fresia P, Cônsoli FL (2015). Comparative transcriptome analysis of lufenuron-resistant and susceptible strains of *Spodoptera frugiperda* (Lepidoptera: Noctuidae). BMC Genomics.

[21] Li LT, Zhu YB, Ma JF, Li ZY, Dong ZP (2013). An analysis of the *Athetis lepigone* transcriptome from four developmental stages. PLoS One.

[22] Henrich, V. C. The ecdysteroid receptor. In: Gilbert, L. I., Iatrou, K., Gill, S. S., editors. Comprehensive molecular insect science. Oxford: Elservier. Pp. 243–285 (2009).

[23] Marchal E (2010). Control of ecdysteroidogenesis in prothoracic glands of insects: A review. Peptides.

[24] Ogihara MH, Hikiba J, Iga M, Kataoka H (2015). Negative regulation of juvenile hormone analog for ecdysteroidogenic enzymes. J. Insect Physiol..

[25] Bomtorin AD (2014). Juvenile hormone biosynthesis gene expression in the corpora allata of honey bee (*Apis mellifera* L.) female castes. PLoS One.

[26] Hao W, Zhang Y, Xu YS (2013). Identification of a juvenile hormone esterase binding protein gene and its developmental and hormone regulation in the silkworm, *Bombyx mori*. J. Insect. Physiol..

[27] Nouzova M (2012). Functional characterization of an allatotropin receptor expressed in the corpora allata of mosquitoes. Peptides.

[28] Shinoda T, Itoyama K (2003). Juvenile hormone acid methyltransferase: key regulatory enzyme insect metamorphosis. Proc. Natl. Acad. Sci. USA.

[29] Ito C, Tomioka K (2016). Heterogeneity of the peripheral circadian systems in *Drosophila melanogaster*: A review. Front. Physiol..

[30] Liu NY, Zhang T, Ye ZF, Li F, Dong SL (2015). Identification and characterization of candidate chemosensory gene families from *Spodoptera exigua* developmental transcriptomes. Int. J. Biol. Sci..

[31] Forêt S, Wanner KW, Maleszka R (2007). Chemosensory proteins in the honey bee: insights from the annotated genome, comparative analyses and expressional profiling. Insect Biochem. Mol. Biol..

[32] Vogt RG, Rogers ME, Franco MD, Sun M (2002). A comparative study of odorant binding protein genes: Differential expression of the PBP1-GOBP2 gene cluster in *Manduca sexta* (Lepidoptera) and the organization of OBP genes in *Drosophila melanogaster* (Diptera). J. Exp. Biol..

[33] Wu C, Suzuki K, Kuwano E (1996). Induction of non-diapause eggs by imidazole derivative KK-42 in the diapause type of *Bombyx mori* silkworm. Biosci. Biotechnol. Biochem..

[34] Liu YQ, Zhang QR, Denlinger DL (2015). Imidazole derivative KK-42 boosts pupal diapause incidence and delays diapause termination in several insect species. J. Insect Physiol..

[35] Wang F, Sehnal F (2001). Effects of the imidazole derivative KK-42 on the females and embryos of *Schistocerca gregaria*. Entomol. Sci..

[36] Shimizu T (2002). Identification of an imidazole compound-binding protein from diapausing pharate first instar larvae of the wild silkmoth *Antheraea yamamai*. J. Insect Biotechnol. Sericol..

[37] Liu YQ (2012). Characterization of a gene encoding KK-42-binding protein in *Antheraea pernyi* (Lepidoptera: Saturniidae). Ann. Entomol. Soc. Am..

[38] Grabherr MG (2011). Full-length transcriptome assembly from RNA-seq data without a reference genome. Nat. Biotechnol..

[39] Pertea G (2003). TIGR Gene Indices clustering tools (TGICL): a software system for fast clustering of large EST datasets. Bioinformatics.

[40] Kim D (2013). TopHat2: accurate alignment of transcriptomes in the presence of insertions, deletions and gene fusions. Genome Biol..

[41] Trapnell C (2010). Transcript assembly and quantification by RNA-Seq reveals unannotated transcripts and isoform switching during cell differentiation. Nat. Biotechnol..

[42] Thompson JD, Gibson TJ, Plewniak F, Jeanmougin F, Higgins DG (1997). The CLUSTAL_X windows interface: flexible strategies for multiple sequence alignment aided by quality analysis tools. Nucleic. Acids Res..

[43] Tamura K, Stecher G, Peterson D, Filipski A, Kumar S (2013). MEGA6: Molecular Evolutionary Genetics Analysis version 6.0. Mol. Biol. Evol..

