# Supplementary material for: Transcriptomic analysis of the prothoracic gland from two lepidopteran insects, domesticated silkmoth Bombyx mori and wild silkmoth Antheraea pernyi
Source: Sci Rep. 2019 Mar 29;9:5313. doi: 10.1038/s41598-019-41864-0 (PMC6440963; doi:10.1038/s41598-019-41864-0)
Supplement: Supplementary file 1 — Transcriptomic Analysis of the Prothoracic Gland from Two Lepidopteran Insects, Domesticated Silkmoth Bombyx mori and Wild Silkmoth Antheraea pernyi [file 41598_2019_41864_MOESM1_ESM.doc]

**Supplementary Information**

**Transcriptomic Analysis of the Prothoracic Gland from Two Lepidopteran Insects, Domesticated Silkmoth *Bombyx mori* and Wild Silkmoth *Antheraea pernyi***

Hai-Xu Bian^1,2^, Dong-Bin Chen^2^, Xi-Xi Zheng^2^, Hong-Fang Ma^2^, Yu-Ping Li^2^, Qun Li^2^, Run-Xi Xia^2^, Huan Wang^2^, Yi-Ren Jiang^2^, Yan-Qun Liu^2^ and Li Qin^2^

^1^ College of Plant Protection, Shenyang Agricultural University, 120 Dongling Road, Shenyang 110866, China

^2^ College of Bioscience and Biotechnology, Shenyang Agricultural University, 120 Dongling Road, Shenyang 110866, China

Hai-Xu Bian, Dong-Bin Chen, Xi-Xi Zheng, Hong-Fang Ma and Yu-Ping Li contributed equally.

Correspondence and request for materials should be addressed to Y.-R.J. (email: [jiangyiren56@126.com](mailto:jiangyuren56@126.com)) or Y.-Q.L. (email: [liuyanqun@syau.edu.cn](mailto:liuyanqun@syau.edu.cn)).

**Supplementary Figures**

**Supplementary Figure 1.** Length distributions of the unigenes of two PGs.

**Supplementary Figure 2.** Histogram presentation of KOG classification for *A. pernyi* and *B. mori* PG unigenes.

**Supplementary Figure 3.** Neighbor-joining tree based on their amino acid sequences of steroid hormone biosynthesis and receptor genes identified in the PG transcriptomes of two silkworms, *A. pernyi* and *B. mori*.

**Supplementary Figure 4.** Neighbor-joining tree based on their amino acid sequences of juvenile hormone related genes identified in the PG transcriptomes of two silkworms, *A. pernyi* and *B. mori*.

**Supplementary Figure 5.** Neighbor-joining tree based on their amino acid sequences of clock genes identified in the PG transcriptomes of two silkworms, *A. pernyi* and *B. mori*.

**Supplementary Tables**

**Supplementary Table 1.** Transcriptome assembly and annotation statistics for two PGs.

**Supplementary Table 2.** The accession numbers of protein sequences used for phylogenetic analysis.

**Supplementary Table 3.** Primers used for qRT-PCR.

**Supplementary Table 4.** Nucleotide sequences of genes of interest in *A. pernyi* PG transcriptome.

**Supplementary Table 5** Nucleotide sequences of the genes of interest in *B. mori* PG transcriptome.

**Supplementary Figure 1.**

**Supplementary Figure 2.**


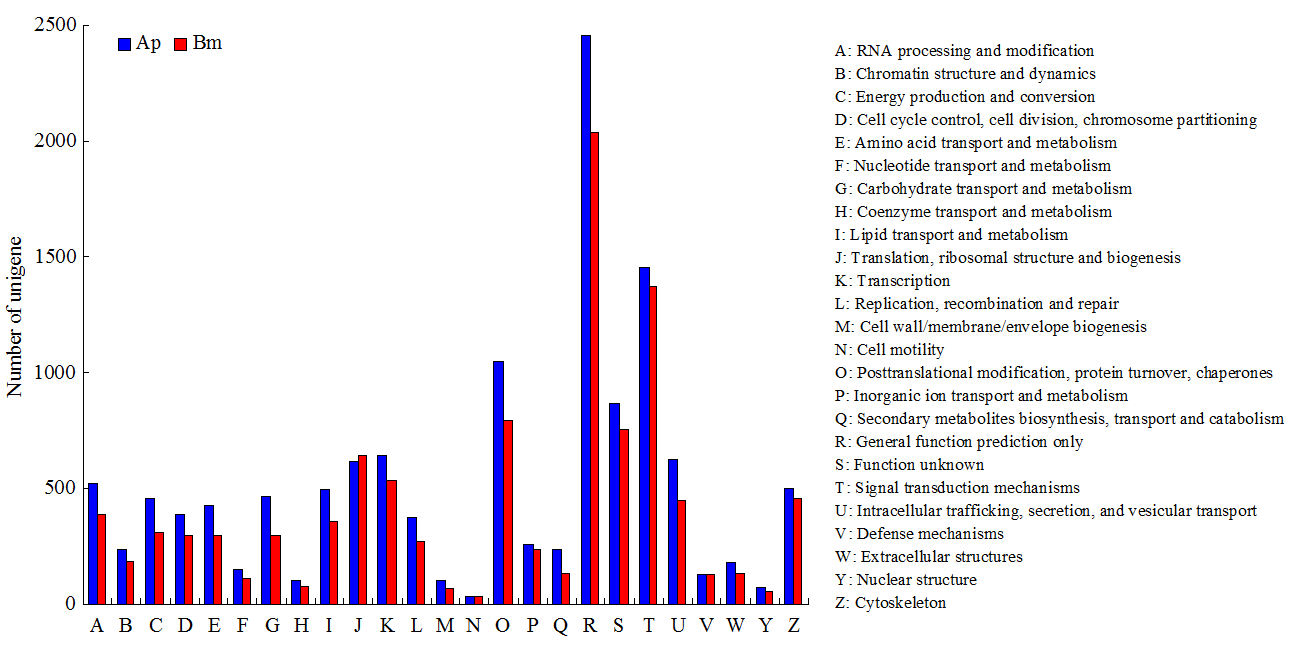


**Supplementary Figure 3.**

ApDIB

BmDIB

DmDIB

DmSAD

ApSAD

BmSAD

DmCYP6u1

ApCYP6u1

BmCYP6u1

ApSPO

BmSPO

DmSPO

DmPHM

ApPHM

BmPHM

DmSRO

ApSRO

BmSRO

ApNVD

BmNVD

DmNVD

ApTORSO

BmTORSO

DmTORSO

100

100

100

100

100

100

100

100

100

100

100

100

100

100

99

100

99

97

91

99

Disembodied

Torso

Shadow

Spook

Phantom

Neverland

Shroud

CYP6u1

A

ApEcRB1

BmEcRB1

DmEcRB

DmUSPA

ApUSP2

BmUSP2

100

100

100

Ecdysone receptor B

Ultraspiracle

B

*Bombyx mori* (Bm)

*Drosophila melanogaster* (Dm)

*Antheraea pernyi* (Ap)

**Supplementary Figure 4.**

BmJHE

ApJHE

TcJHE

DmJHEBP

ApJHEBP

BmJHEBP

BmHEX

ApHEX

TcHEX

BmJHBP

PmJHBP

ApJHBP

DmASTR

BmASTR

ApASTR

BmJHEH

ApJHEH

TcJHEH

DmJHAMT

BmJHAMT

ApJHAMT

BmBROAD

ApBROAD

DmBROAD

ApFPPP

BmFPPP

DmFPPP

ApALDH

BmALDH

DmALDH

DmcJHBP

BmcJHBP

ApcJHBP

100

100

100

100

100

100

100

100

100

100

100

100

100

100

100

100

97

97

100

82

79

74

100

64

79

Hexamerin

*Papilio machaon* (Pm)

*Drosophila melanogaster* (Dm)

*Antheraea pernyi* (Ap)

*Tribolium castaneum* (Tc)

*Bombyx mori* (Bm)

Juvenile hormone acid

methyltransferase

Juvenile hormone esterase

Juvenile hormone esterase

binding protein

Cytosolic juvenile hormone

binding protein

Aldehyde dehydrogenase

(NAD+)

Farnesyl diphosphate

phosphatase

Broad

Juvenile hormone

epoxide hydrolase

Allatostatin C receptor

Juvenile hormone

binding protein

**Supplementary Figure 5.**

BmCRY1

ApCRY1

DmCRY1

DmPHR6-4

BmCRY2

ApCRY2

DmCK2b

BmCK2b

ApCK2b

BmDBT

ApDBT

DmDBT

BmSGG

ApSGG

DmSGG

DmCK2a

BmCK2a

ApCK2a

BmPER

ApPER

DmPER

BmVRI

ApVRI

DmVRI

DmPDP

BmPDP

ApPDP

BmTIM

ApTIM

DmTIM

DmSLMB

BmSLMB

ApSLMB

BmMET

DmMET

BmCLK

ApCLK

DmCLK

DmCYC

BmCYC

ApCYC

100

100

100

100

100

100

100

100

100

100

100

100

100

100

100

100

100

100

100

100

100

100

100

100

100

100

100

100

100

100

98

100

94

100

96

81

Cryptochrome 1 (dCRY)

Cryptochrome 2 (mCRY)

Casein kinase 2 beta

Double time

Shaggy

Casein kinase 2 alpha

Period

Vrille

PAR-domain protein 1 *ε*

Timeless

Slimb

Cycle

Clock

Methoprene-tolerant

Photolyase 6-4

*Bombyx mori* (Bm)

*Drosophila melanogaster* (Dm)

*Antheraea pernyi* (Ap)

**Supplementary Table 1.**

|  | *A. pernyi* | *B. mori* |
| --- | --- | --- |
| Assembly |  |  |
| Total assembled reads | 28,159,208 | 24,408,498 |
| Total clean base number | 7,093,122,543 | 6,149,312,641 |
| Q30(%) | 91.73% | 91.35% |
| GC(%) | 44.55% | 45.75% |
| Transcripts |  |  |
| Total number of transcripts | 64,301 | 49,287 |
| Sum transcript length (Mb) | 42.23 | 52.44 |
| N50 transcript length (bp) | 1,075 | 1,971 |
| Average transcript length (bp) | 657 | 1064 |
| Unigenes |  |  |
| Total number of unigenes | 44,067 | 32,302 |
| Sum unigene length (Mb) | 24.19 | 25.77 |
| N50 unigene length (bp) | 865 | 1,510 |
| Average unigene length (bp) | 549 | 798 |
| Annotation |  |  |
| GO | 10,621 | 8,557 |
| KEGG | 5,820 | 4,665 |
| KOG | 11,506 | 9,321 |
| Pfam domains | 10,974 | 9,188 |
| Swissprot | 10,283 | 8,791 |
| TrEMBL | 19,369 | 15,394 |
| NR | 19,305 | 15,187 |
| All | 22,402 | 23,157 |

**Supplementary Table 2.**

| Gene | Accession number | Description |
| --- | --- | --- |
| **Steroid hormone related genes** | | |
| DmNVD | NP_001097670 | neverland [Drosophila melanogaster] |
| DmSPO | NP_647975 | spook [Drosophila melanogaster] |
| DmPHM | NP_573319 | phantom [Drosophila melanogaster] |
| DmDIB | NP_524810 | disembodied [Drosophila melanogaster] |
| DmSAD | NP_650123 | shadow [Drosophila melanogaster] |
| DmCYP6u1 | NP_610252 | CYP6u1 [Drosophila melanogaster] |
| DmSRO | NP_651725 | shroud [Drosophila melanogaster] |
| DmTORSO | CAA82998 | torso [Drosophila melanogaster] |
| DmEcRB | NP_724460 | ecdysone receptor B [Drosophila melanogaster] |
| DmUSPA | NP_476781 | ultraspiracle [Drosophila melanogaster] |
| **Juvenile hormone related genes** | | |
| DmFPPP | CG10352 | farnesyl diphosphate phosphatase[Drosophila melanogaster] |
| DmALDH | CG31075 | aldehyde dehydrogenase (NAD+) [Drosophila melanogaster] |
| TcJHE | NP_001180223 | juvenile hormone esterase [Tribolium castaneum] |
| DmcJHBP | AAL39751 | cytosolic juvenile hormone-binding protein [Drosophila melanogaster] |
| PmJHBP | KPJ16053 | juvenile hormone binding protein [Papilio machaon] |
| TcJHEH | NP_001161902 | juvenile hormone epoxide hydrolase [Tribolium castaneum] |
| TcHEX | NP_001164358 | Hexamerin [Tribolium castaneum] |
| Dmbroad | NP_726750 | broad [Drosophila melanogaster] |
| DmJHAMT | NP_001285980 | juvenile hormone acid methyltransferase [Drosophila melanogaster] |
| DmJHEBP | NP_611989 | juvenile hormone esterase binding protein [Drosophila melanogaster] |
| DmASTR | AAL02125 | allatostatin receptor [Drosophila melanogaster] |
| **Clock genes** | | |
| BmMET | [NP_001108458](https://www.ncbi.nlm.nih.gov/protein/169234673?report=genbank&log$=protalign&blast_rank=1&RID=JZR6KJ6P014) | methoprene-tolerant protein 1 [Bombyx mori] |
| DmCRY1 | BAA35000 | blue light photoreceptor [Drosophila melanogaster] |
| DmPHR6-4 | NP_477188 | (6-4)-photolyase [Drosophila melanogaster] |
| DmPER | CAA27285 | per protein [Drosophila melanogaster] |
| DmCLK | AAD10630 | CLOCK [Drosophila melanogaster] |
| DmCYC | NP_524168 | cycle [Drosophila melanogaster] |
| DmVRI | NP_723075 | vrille [Drosophila melanogaster] |
| DmTIM | AAC46920 | TIM [Drosophila melanogaster] |
| DmSGG | NP_476714 | shaggy [Drosophila melanogaster] |
| DmPDP1 | NP_001261546 | PAR-domain protein 1 [Drosophila melanogaster] |
| DmCK2a | NP_524918 | casein kinase II alpha [Drosophila melanogaster] |
| DmCK2b | NP_542940 | casein kinase II beta subunit [Drosophila melanogaster] |
| DmMET | NP_511126 | Methoprene-tolerant [Drosophila melanogaster] |
| DmSLMB | AAC38852 | Slimb [Drosophila melanogaster |
| DmDBT | AAC39134 | casein kinase I homolog [Drosophila melanogaster] |

**Supplementary Table 3**.

| Gene | Primer sequence | Product size /bp |
| --- | --- | --- |
| *cryptochrome 1* | *cry1*-F: CCACGACAATCCGTCTCTT  *cry1*-R: GGTTGTACCCGACCACTTTCG | 110 |
| *cryptochrome 2* | *cry2*-F: GGAGCCACTACACCGATTAC  *cry2*-R: CTTCACTTTCTCCGCCTATCC | 121 |
| *period* | *period*-F: GAAACGGAAACTGTATCGC  *period*-R: GAGGCAACAGAAGTAGTCA | 115 |
| *timeless* | *tim*-F: CTCTGCTCGGTCTTGTCATT  *tim*-R: TGCACGGCTTGAGACCATTA | 119 |
| *clock* | *clk*-F: TTTACCTTTGCCGCCTCTAC  *clk*-R: CTGTCGCAACTCCTCTTGTT | 118 |
| *cycle* | *cyc*-F: AAACGGAAACCATCGTCCTA  *cyc*-R: TTTGTTTCTTGTCGGGAGTG | 118 |
| *shaggy* | *sgg*-F: CGTCGTCTATCAAGCCAAACTA  *sgg*-R: CGTCGCATGATTTGAAGTTCTC | 105 |
| *double-time* | *dbt*-F: AAACGAGACATCCACAGTTACA  *dbt*-R: CGGACCTAGCAGTTCCATTAC | 134 |
| *vrille* | *vri*-F: GCTATCCCTCCAGCACATAAA  *vri*-R: CGGTTGCGGTCTGTATTCT | 124 |
| *PAR-domain protein* 1 | *pdp*-F: GAAGGATGACAACGACCTCTG  *pdp*-R: TTCTCCGACAGGAACTCATCTA | 123 |
| *slimb* | *slmb*-F: CACAAGATGGAACGAGACAGAT  *slmb*-R: TTGTAGCATCGGTTTGAGGTAG | 109 |
| *casein kinase II alpha* | *ck2a*-F: AGAGGCACTGGACTTTCTTG  *ck2a*-R: CCTGCTCCTTTACGATTGGATA | 110 |
| *casein kinase II beta* | *ck2b*-F: TGATACATGCCCGGTACATATTG  *ck2b*-R: GACAAACCAATTGGCAGCATAG | 130 |
| *Farnesyl diphosphate phosphatase* | *fdp-*F: CTGGTTACGGCAGAAGTGAAA  *fdp*-R: GGCGATCATGTCACCGATAAA | 132 |
| *Aldehyde dehydrogenase (NAD+)* | *nadf*-F: TCGCGCCAACAATACAAATTAC  *nadf*-R: GAGTCCTTGAATCCTCCGAAAG | 159 |
| *Juvenile hormone acid methyltransferase* | *jhamt-*F: CAACGAGAGAATGGTCAAACAC  *jhamt-*R: GTCCTTTCATCTCATCGGGAATA | 104 |
| *Juvenile hormone epoxide hydrolase* | *jheh-*F: CCTGGTTATGGCTTTTCCG  *jheh-*R: GCTCCCCAATCACCTCCTT | 131 |
| *Juvenile hormone esterase* ^[1]^ | *jhe*-F: GTGGGCTCAGTAAGGGCATA  *jhe*-R: ATGGCTCTGCTGGTTGTAGT | 173 |
| *Cytosolic juvenile hormone binding protein*^[1]^ | *cjhbp-*F: TTGGAACGGTCTACTGACTTTG  *cjhbp-*R: GCGTCTTGGATCTTCTTGTCTAT | 195 |
| *Juvenile hormone binding protein* | *jhbp-*F: CCTTGGACCCGTTACACATATC  *jhbp-*R: TAACACCTTCCACGTGACAAC | 111 |
| *Juvenile hormone esterase binding protein* | *jhebp-*F: CGGTGACATGCCTCCAATAA  *jhebp-*R: ACCCACCAACTGTCTTCAAC | 117 |
| *Hexamerin* | *hex*-F: GCTGCCGAGCATCAGTATTA  *hex*-R: AAGTGAAATGTCCTCCCGAAA | 126 |
| *Broad* | *broad-*F: TGATGTGGAGTATGAACCAGAAG  *broad-*R: GGACATGCCAGCAAGTAGTAA | 112 |
| *Allatostatin receptor* | *all-*F: CTTCAGCCAAGGCCAGATTA  *all*-R: CGAATGACGAGGCAGTAGAAA | 99 |
| *Ribosome protein* 49 | *rp49*-F: GGGTCAATACTTGATGCCCAA  *rp49*-R: TCGTCACTCTGATGCTGAGC | 221 |

[1] Zhang, Z. *et al*. Depletion of juvenile hormone esterase extends larval growth in *Bombyx mori*. *Insect Biochemistry and Molecular Biology* **81**, 72–79 (2017).

**Supplementary Table 4.**

| Gene | Nucleotide sequences | |
| --- | --- | --- |
| **Steroid hormone related genes** | | |
| *Neverland* | | GCCCGGATCAGTGATGGACTAAAGTGGCTGTACAGTTCACGTGTGCGCCTTTGCCTATTGATTTTTTTTTTTATTCCATTTTTATTTCGCGGTATTTTTTTTGCGCGGTTAAAATATAACCTAGAATATGAAATAAGAAACTACGAAAACAGCCACAGAACCACACACGGAATGGCTGAATGCATAAATAAAATGGCGAATCAAAATTCATATCACCGTTGCCACGACGCCATTTTGACTTCTGAATTCAAATACGACTTCTTAATATTCTATTCAAACTTATTATTCGCTGTATCGAGATGGATCTTCGAGTTCGTTTACGATCATTTATTGATGATATTCATGGCTGTGATAGTTGTCTTCGTATTTTATATTATATATAAATCTTTTTGGAACCCTATACTTTACACTAAGGAGTTAACAGAAGTAGGCTATGAGCATATACCACTGGGACCAGACAGAGATCGAAGGATAACTAGAGCCCAAGTTGCTAGACGTATGGGCTCCAAGATTCCACCACCGTACCCTAACGGTTGGTTTGCTGTCACCGAGTCTAGAGACTTGAAGATAGGAGATGTGTTATCCGTCGATGCCCTCGGTCAAAATTTATGCATATATCGTGGTGAAGATGGTATAGCTCGCTGCGTAGACGCTTACTGCCCACATTTGGGAGCGAACCTCAGTATAGGCGGTACCGTGTGTGGTAACTGCATAGAATGCCCATTCCACAAATGGAAATTCGGTGCAGACGGCACTTGTATCAGCGTACCGGGTGTTGAAACTGCTCCAAAGGGTGTGTCCATAAAGACCTGGCATACAGTGGAAGCGGACGGCGCTGCCTGGATATGGTACGACGCTGAAAATCGCTTACCACAATGGACTCTGAAAGACTCGCCGGAATTTAAAAAATTCGCTTACCGAGGAAGGAATGAATTCATCGTCAACGCCCACGTACAGGAAATCCCAGAAAACGGTGCAGATGTTGCCCATCTAAATGCAGTACACTCACACTCTATGGTGTCTGATTTGGGTGTTAAATATCCCAAATTATATGAACTCATAGGAAGGCACGTGTGGTCCGCTGAATGGACTAAGGGCGAAGATCACACCTCTCTCATGCAGATATCTCATGACTACAAGATTCTTAAATTCGATATGGCACACACTGAGGTCAAAGTGAAACAGGTCGGACCAGGTCAAGTTAGCCTTCTAGTGGAGTTTCCATTAGGGCAAATATATGTGGTACAATCAGTTACCCCTCTTGGACCTTTAACACAGAAGGTCGTCCATCGGATGTACTCTCCGTACTACAACGCTCCTTTGGCAGCACTATTTGTTAAAATCGAGGGGGCGATGTTCGAACGTGACGTAAGAATATGGAACAGCAAGAGATTCGTCAGCGCCCCAGCTTATGTACGGACAGACAAAGCGATAAGAGCTTTTAGAGCTTGGTTCTCACAGTTCTACAGCGAAAATAGCGTCACATTTCGGGACGCCTTACAGAGTCCTCTCGATTGGTGATTGTTAGTATTAACCCTGCTCAATAGATGGCGCTAAAATTCATAAGCACAAAGTAGACCATTTCAGAGTAAACTTCATATGAAGTTTAAGAAATTATTATAGTATTAACACTTTTAAATAGACGACGCTTAAGTGTACAAACATTGAATATATGACGTCAGAGTATATAACGATTAGAAAACTCTTTTAATATTCATTTTGTTAAACAGATGGCACTAAAGTGTAAAAACGAGAAGTCGATGATGTCGTTAAAGTTCTTGTGATGTTTAGTATTAACTCTTTTAAATAGATGGCGCTAAAATGTATAGGTTTGATAAGTAGACGTCGTCATAATAAATTGGTTAGTGAAATGGTGAATCACATTTGTAACATAGCGTAATTTAAATTGAGGGATTTATTAATTAGTAATACTTTAAATATGCATAATTCAGAAGTAGAAGTTATTTGAAAAAAAAAATCTCAAAAAAATTCGACTTTCGACAATCTCATCTAATGTAAATGTGATGAAGGTGTGTATGTAATAAAGGCGAAAATTAATCCATTTGTTCATGCCTTATTTACTCAACCGATCATTATAAAATTTTAAAAACTAAAACTAGTTATTTTTGTACATCTATCTACTGGTGCTGTAAATAATGAGTATTCGATAGTGTTTTAAGTAACTCTGCTTTTTATTCCTTGTAAAGGTTAAATATATATTTGTGTTTTATTTCTAAGGGTACAAAATTAGTTTAGTTTTAATTTTAAAATAAAAGCCTT |
| *Spook* | | AGTGGCCACACCGGGAGTTTGGCAGTGCTGGCTGGTGCTTAGTTCCCGCCAAACTCCCGGTGTGGCCACTCAAAAAAATTCTTTTTAGAATGAGTGCACTTCTTCTGATAGCCGTCATCGCGTTCACGGTGTACAAGTTCTTTAAAAAAGACACCGTGACGTGGAAACGCGTGAACAAGTACGGCGGAAACCAATCAGCCGTACTGAAAGAAGCACCGGGGCCAATCCCTTTGCCAATAATCGGGAGTTTACATCTGTTGGGAAAACGGGAATCTCCGTTCCAAGCGTTTACCGATCTGGCCAAGGAGTATGGGGATATATTTAGCATCAAACTGGGAACTGCGAAATGCTTGGTCGTCAATAATCTATCCTTAATAAAGGAAGTATTGAACCAAAATGGGAACATCGTCGCCGGACGTCCCGATTTCCTTCGGTTTCATCAACTCTTTGCCGGTGATAGAAACAATTCTTTGGCTCTCTGCGACTGGTCGAACCTCCAACTGCGCCGTCGGAATCTCGCCAGAAGACACTGTGGCCCCAAACAGCACACCGACTCGTACGCCAGAATTGGATCCGTCGCCACCTTCGAATCCTACGAACTCATCCAAACATTAAAGAACATCACGCAGTCCACAGAATCCAGCATTAACATAAAGCCTATCCTAATGACAACAGCTATGAACATGTTCTCCCATTACATGTGCAGCGTCAGGTTCCAAGAGGACGATTCAGAATTCCGCAAAGTAGTCGACCACTTCGACGAGATCTTCTGGGAGATAAATCAGGGTTACGCCGTAGATTTCATACCCTGGTTGGCGCCATTCTACAAGAAACACATGGAGAAGCTTTCAAACTGGTCCCAGGATATACGTTCCTTCATTTTGTCAAGGATAATCGAGCAACGCGAGATGAATCTCGATACCGAAGCGCCGGAAAAAGATTTCCTTGATGGTTTATTACGAGTGCTGCATGAAGATCCAACTGTTGACAGGAATACGATTATCTTCATGCTGGAGGACTTCCTTGGAGGACATTCGTCCGTGGGTAATCTGGTAATGTTGGCTCTCACAGCTGTCGCACGTGATCCAGAGGTGGGGATGCGTATTCGAGCGGAAATCGATGGCCTGACTAAAGGGAAACGAATCGTGAGCCTAACCGACAGACCTAGCTTGCCGTACACTGAAGCTACAATTCTTGAATGTCTTAGATATGCGTCCTCTCCTATAGTACCTCATGTGGCGACTGAAAATGCGAATATTTCCGGCTACGGTGTCGAGAAAGGTACGGTGATTTTCATTAACAATTATGTTTTAAACAATTCCGAACAGTATTGGAAGGAGCCAGAGAAGTTTGACCCGAGCCGTTTCTTGGAGAAGACCAAAGCAAGGGTGCGGCGGAACTCGCAGTGTGATTCGGGTATGGAGTCCGATAGTGAAAGGCATGGGCCGGTGAAACGTGAGACAGAGAAAGAGGTCTACAGCGTTAAGAAGAATATACCGCATTTTATGCCATTCAGCATTGGCAAAAGAACGTGTATAGGTCAGACTTTAGTCACGACGATGAGTTTTACGATGTTCGCGAATATTATGCAGGAATTCGACGTGGCGGCGGAAAATATAGAAGATTTAATACAGAAGCCGGCTTGCGTTGCCCTACCTAAAGATAGTTATAATTTATATCTTATACCGAGAAAGCACTGAAACATATTGGACTGTAAATTAAAACTGAAGTTGCAATGTGTTCTTTTTCTGCAATGGGCGTTGGGGTAACATTTGCCAGTGTCAAACGTTATATGTAACTATATTTCGAATTGACGTTATCCGGGGCGTATGTTCTAATTACGTGATAAAGTGGCAATTTTCTAGTGTTATTTTGATACACTTTAAAAAAAAGTGTGTGTGTGTACTTTATGTACACGCGTTAGAAGTTATACTTCTTTGGCGTAACAAGATAAAAATCTTTTCAAATTTTTTTTCTTTCGCCTTATTCTACGTTTGTAGAAAAAACGACACTAACAGTAGAAAAAGTTGATTATACCCAACTTCAGGGTCGGTTTTACGTGACGGTGTGCGCGCGCATCGTAATAATTTACTCTCATCATTTTTCCCTAACGCGCCCAAAGAAGTATAACTTCAAAAAACAAAATTAACAAAAAAAAAAAAAAAATTATTTAC |
| *Phantom* | | GGGGGGGCAGTCGGCAACCGTCGGGCCACGTGATTAGTGGTCTCTGTTGCAGCGAATTGACATCGCGATACGATATTCAATATCTTTGTTTAAAAAGCCTATGAGCATCTTTTATACAACTAAACGAACTTAGGTGTTTGTGAAGTTCAAATGGATCTATTTTTTATATGGTTAATAACTTTCGTGGGGGGATTTTGGATTTTCAAGAAGATTAAGGAATGGCAAATATTACCTCCTGGTCCTTGGAATTTACCAATTGTCGGTTATCTTCCTTTCATCGAGCGTTACCATCCACATTTGAGTTTGACTAAGTTATCTAAAGTCTACGGTCCAATATATGGAATAAAAATGGGTAGTATATACAGCGTTGTTTTGTCAGACCACAGATTAGTTAGAGAAGCTTTCGCTAAGGAAAGTTTTTCTGGAAGGGCTCCTTTGTTTCTCACACATGGAATAATGCATGGAAATGGAATTATCTGCGCTGAAGGTGCATTATGGAAAGATCAAAGAAAACTGATAACGTCATGGCTTAAAAGCTTTGGAATGAGCAAAAACAGCGTGTCTCGCGAAAAGTTGGAGAAACGTATCGCATCTGGAGTTTGTGAACTGCTTGAAAATATAGACAAATCGTCGGAAACTCCATTGGACTTACCTTGCATGTTGTCAAATTCACTCGGAAATGTAATCAATGAGATAATATTCGGATTTAAGTTTCCCCCTGAAGATAAAACATGGCACTGGTTTCGTCAAATACAAGAAGAAGGTTGCCATGAGATGGGAGTCGCTGGTGTTGTAAATTTTCTACCTTTCGTTCGATTTATTTCATCGTCTACTCGAAAAACTATTGAACTATTAATACGTGGCCAAGCGCAAACACACATGTTATATGCGAGTATTATAGATAGGCGCCGTAAAATGTTGGGGATAGATAAACCAAAAGGTGCAGAATACTTACCACATAATAATTTATTGAATGAATATCCTAACGGACATATAAAATGCATAAAATACAGCAAGTACGCACCAAATACGGAACATTTTTTTGATCCCCAAACATTAATACCGACAGAAGGTGAATGTATTTTGGATAACTTCTTAATGGAACAGAAGAGTAGATTTGATAACGGCGAAGAAAATTCACAGTTTGTGACAGATGAACAGATGCACTATCTGTTGGCCGATATGTTTGGAGCAGGCTTAGATACAACTTCAGTCACGCTCGCCTGGTTCTTTCTTTACATGGCCCTGTACCCTGACGAACAAGAGATAATTCGCAATGAGATTATCAAAGTGTATCCCGAAGAGACTGACGTGGACGGATCAAGATTGCCACATTTAATGGCAGCTATTTGTGAAACTCAACGTATTCGATCCATTGTGCCGGTAGGCATTCCTCACGGTTGTACTAAGGACACTTATTTGGCAAACTATTTCATACCAAAAGGAACTATGATAATACCTCTTCAGTGGGCTTTACATATGGATCCTGGTGTTTGGGAAAATCCTGAGGAATTTAAGCCTAGTCGATTTTTAGCTGAAGACGGTAGTTTGTTGAAGCCACAAGAGTTTATACCTTTTCAAACAGGAAAGCGCATGTGCCCCGGTGATGAACTGTCCAGGATGATATCGTGTGGTCTTGTGGCCCGACTGTTCCGTCGTAAACGTTTACGTTTGGCATCTGAACCACCGGCTCCTGACGCAATGAGAGGAACCGTCGGCTTAACCCTTGCTCCTCCACGTGTGAAGTATTTTTGTGATCCTATATAGATAATACAATTGATCTCACACGTAGATAGTATAGTTTATACGACAATTAGTTATTCTGTTAGCATCGGAAAAATCATTAAAATTTTGGGTAAACCTCAATTTTGTTTCATTTTTTTTGTTTTGTTTTGTTGCTAGATTTCTTATAAATGTATCTGGTACACATCAGGAATCCCATAGGATTAATTGTTGCTCCTCCAACATGTGGAGTGTTTTGTGGTCCTAGGTATATAAATAATATTATGAATCTTATTTATCTTATTATAGATTTATACGACAAGTAGTTATACTGTTGTCATTGGTAAAATCCATTAAAATTTGGTTAAGCCTCGAATTTTGTTTCATTTTAAATATTTTGGTTTTAAATAATATCACCCTGATATCAATTTTTAAAGGTTCTAAATGCGGGAAGAGACGTTGAATTAATTATTAGAGACCTCGATGTAAAGATTATTCACCAGATTTATTTTAATGTATCTGGTATATATCAGGAATCTCATCGAATTAATACTTGCTCCTCCACATGTGGAGTACTTTTGTCATCCTATACAAATAATATTATAAATCTTACACGTAGATAGTACGTAGGTTTATATGACAAGTAGTTATACTGTTGTCATTGGTAAAATCCATTAAAATTTGGTTAAGCCTCGAATTTTGTTTCATTTTAAATATTTTGGTTTTAAATAATATCACCCTGATATCAATTTTTAAAGGTTCTAAATGCGGGAAGAGACGTTGAATTAATTATTAGAGACCTCGATGTAAAGATTATTCACCAGATTTATTTTAATGTATCTGGTATATATCAGGAATCTCATCGAATTAATACTTGCTCCTCCACATGTGGAGTACTTTTGTCATCCTATACAAATAATATTATAAATCTTACACGTAGATAGTACGTAGGTTTATAT |
| *Disembodied* | | CAATGGACAGTACAAAGCGATCTTTTAAGTCAGTGATGCGATACAATACACATAAACTGCTTGTCGTTTTTGTTGCTAGTTGTTTATAAAAATGAATAAAATAATTTCGTTTGGAAAGTATTCACCACTTTTCTTTTTAAAATGCGCGAGAATAAGTTCTGCCGTGTATACCGTGGAGAATAACCAAGAAAAAGTAAATATGAAAAAAGAGACTATTATGCATTTCGACCACATACCCGGTCCCAAGTGTTATCCCGTCGTAGGCACTTTGCATAAATATGTACCATTCATTGGTGATTATGACGCGGAGGCTCTTGACCAAATCGCGTGGTTAAATTGGCGGCGCTACGGCTGTCTTGTTCGTGAAAAACCTGGGGTAAACTTAGTACACGTGTACGATCCTGACGACATTGAGGCGGTATTTCGACAAGATGATCGTTATCCGGCGCGGAGGAGTCACATCGCTATGTTCGATTATCGTATGAAGAAACCGCAAGTTTATAATACCGGGGGATTACTTTCAACGAACGGTGCAGAATGGTGGCGCCTGCGGAGTACCTTTCAGAAAAATTTCACTAGTCCACATAGCGTAAAGGCACACGTTGGAAATACAGATGCCGTGATAAAAGAATTTGTACAATGGATTAAGAATAACAAGACGTCGCACCACGAAGATTTTCTATTATATTTGAACAAGTTGAATTTAGAAATTATAGGAGTTGTAGCTTTCAACGAGAGATTCAACAGTTTTGCTCCAGAAGAACAAGAACGCCAATCACGAAGTATTAAAACAATAGAAGCAGCGTTTGGTTCAAATTGTGGTATTATGAAGCTCGATAAGGGATTTCTTTGGAAATTTTTTCACACACCGTTATATAGAAAGCTTGCCAATTCTCAAGAATATTTAGAGAATGTCTCAACCAAAATTTTGATAAAGAAATTAAATTTCTTTGAATCTGATGATAAAATCTCGGATAATTCGCTCTTAGGATCATTTTTGAAACAGCCAAATGTGGATCTCAAGGATATTGTTGGCATGATGGTTGATATTTTGATGGCAGCTGTTGACACGACTTCATATACGACAAGTTTTGCCTTATATCATATAGCTCGAAATTGGGAGTGTCAAGAAAAAATCTTCTCTGAAATTGCTACACTCCTACCTGATAATAATTCAGAAATAAGTGCTGATACAATTACAAAAGCTGTATATTTAAGAGCTAGCATTAAAGAGAGCTTAAGATTGAATCCAGTGTCTGTTGGAATTGGTCGATTGTTACAAAAAGACATAATTTTAAGAGGATATCAGATCCCTAAAGGAACAGTAATTGTGACTCAGAATATGGTTGCATCGCGTCTACCACAATATGTTCAAGACCCATTACAGTTTAGACCAGAGCGCTGGTTACGTGGATCTTCTCAGTATGAAAATATTCACCCTTTCTTAAGTTTGCCTTTTGGTTTTGGTCCGCGATCTTGTATTGCAAGAAGGCTAGCAGAGCAGAATATGTGTATAATCATTGCTCGGTTGGTACGTGAATTTCAAATCAAATGGATGGGTAAAGAACTTGGTGTGAAAACATTGCTTATCAATAAGCCTGACCAAGCTGTATCGTTATCACTTGTATCAAGGAATATATAATTCACGTAACAATCTAAGAAGAGTTTGTAAAAAAGTAAAATAATATTCATAAATGTTGGTATTTATTTATTTCACGTTCATAAATTGAAGTTTACACAATAATGTTAGGAAAAACAGACGCCGTAAGGACTGGGTCTTCGGCTGTTGGAATGGCAATCAGTTCCATACTTTTTGTGAACGTTTTCTTGTTTATCACAATATTAGGTAGTACACGTTCTACAATAATGTCTCGAGCATATTCAGTTGGAATTGATAAATCAAATGGTTTTTGAAGAAACTTTCGATCAACTGAATAAGCTGGGTTTATTAAGGCAGGAATTTCTGGAACTGGAGGCATTAATACTACTGTTTCTTCTTCGTTTATAATCCTTGAAATATCAGGCATTGCATAAGTAGGAATTTGTAGAGTACCGGGAATTTGAATAATTTTTATCATTAATTGTTTTGGCACAAGGCGAAGAAAATCATTTTCTAAGATTCTTATTTGAGGTTCGATAGGATTTATAGGTATCACTTTAGGTACCGTCGTTTCCGTTTCATTGTATGGTGGAAATATCTTAACGGGTCCTTCTATAATGTTGGTTATTTCTAAAAGATCTAATGGGGGCAAGATTGGTGCTGGAGGGATTCTTTTCAAAATTATATCGTCAGGTATTAACGGAAATTCAATAGGAATGAGTGGTGGTGGAAAAGGAGGAGGTACAATTATCGATTCATTAATTAATATTGGTGGAAAATTCGTGGGTGTTATTGGTTCAGGCTGAGTATTATTACCAGGTAATTCTAGATCTTCAGTTATTACAGGTGTTTCTATAATGTTTTCATTTACAGTGGCTACAAACCTTCCAATTTGTGGTGGAACAAAAGTTCCTAACAAAAGTTGTGGGTTGAAGAAATAGTTAGTTCCTTGATGAACGAAATTAGAGACAAAAGGTGACGGTTCAGATGATAACACAGGGATGGGTTCCCTAAAATTTTCGTTCACAGTGATTTGAGAAGGTACATTTTGGTCGGTCGTTGGTAATCCTCTTGAGTGAGCAGCCAGCACAATAAAAACATACAGCCTCAATTCTAAGTAAATCTGAAAAAAAAGTGTTAGAATTAGGTACAATTCTAATTAATAAATGGATACATAAAAAAATATCAATAATTTTTTTTAACTATTACTTACCATGATAAAGATTGCCGTTGATCTATTTTAGATTAGTGCGCGACATACAATTTATAGTAGGAATTCTTTATTTTTAAATACTTGTTAATTTGATATATAATGTTAACCCTATTAAAAATTCATTAAAACTAGAAATGAAATTGCTAATTAAACAAGTAATTCTAGTGTTAATTACAAGTAGTATAATTGTTATATAATATAAATACGGCGC |
| *Shadow* | | ACGGTCAGCAAAATACATTATTCACTGGATTATTGTAAATGCAAATACGATCACCGGGCGAACAACGGTAAAAACGCTATTTAGAATTGAGAGGATTTTATTTCAGTTTATACCAATCTCTTGGACTGTGTTCACTTCTTCAATACATTTGTAGCGATGCATCGTTGTGCGATTATTTCAAAATGTAAAAGAATGTCAATATATGAAAAAAGACATATAACTACATCAATAGAGAATATGAAATCGATTCTTACTATAGATGATATACCGCATCAAAAGTACTTGCCTATTATAGGAACAAGATTAGATTTTTTTATTGCTGGAGGTAGTACGAGATTGCACGAGTATATAGATTTCCGCCACAAACAATTAGGATCTATTTTCCGAGAAAAACTTGCTTCAAGCTCAGATCTTGTATTTATTAGTGACTCAGAGTTGATGAAGTCGCTTTTTATACAACTAGAAGGAAAATATCCAGCGCATATTTTACCTGAAGCTTGGGTATTATACGAAAAATTATATGGTTCTAAAAGAGGTCTTTTCTTTATGGACGGTGAAGAATGGTTACATAACCGTCGTATAACCAATAAACATTTACTTCGAGAAGAATCGGAGAAATGGCTACAGAAGCCAATCGAAGAAACTATACAACGTTTTATCCAAAATTGGAAAATAAAAGCAAAGCAAAATCCCTTTGTACCTGATTTAGAATCGGAATTTTATAAACTTTCGACTGATGTAATTATAGCCATTTTGTTGGGTAATAATTCTTCCATAAAGCCGGATAAATATTATGAGCAGTTGTTAAATGTGTTTTCGGAATCAGTGAAAAAGATATTTCAAACAACAACAAGGTTGTACGCTTTACCGATTAATATATGCCAAAAATTCAATTTCAAAGTTTGGAGGGATTTTAAAGAATCAGTTGATGTGTCACTTTCTTTAGCTCAAAAAATTGCGTCTGAAATTTTATCTAAAGCACATGAAAGTGACGGTTTAATTAAAAGATTATGCACTGAAAATATGCCTGACAAAGATATAACAAAAATTGTCGCAGACTTTGTGATTGCAGCAGGAGATACGACAGCTTATACGTCTTTGTGGATTCTATACCTACTATCCAAACATGCTGAAATCGTTAATGAAATTCGCTCGAAGGATTCTACCTATATTAAATATGTTGTAAAAGAATCAATGAGATTATACCCCGTAGCTCCATTTTTAACAAGGATTATACCGAAAGAAAGTATTTTGGGTCCTTATAAACTGCACGCTGGTACACCAGTTATTGCATCGATTTACACATCAGGCAGAGATGACAATAACTTTAGTAGACCACATGACTTTTTGCCTTACCGATGGGACAGGAGTGATCCTAGAAAGAAAGAATTGATAAACCACATTCCATCGGCCTCACTGCCTTTTGCGCTTGGCTCTAGATCATGTATTGGGAAAAAAATTGCAATGCTTCAGATTTCAGAGCTAATAAAACAGATTACTGATAATTTTGATTTGGAATTTATAGGCGAAAATGAGGTGAAAACTGTTACTTCACAGATCCTGGTACCTAATAAAAAGGTGGAAATTAAACTAATACCTCAAAAATAAATTAATGTATCAATAAAAATAATATATTTGATAGTTACAGTGAGTTTATTTATAATACATAAATTTCAAAGGAAATACATGACACATAAATAATATATTGTTAAATCCCATATATTGTACATACATTGTAAATTACACACACCAAGAGTAAAAGATAATTTCATTGATTTAAAGGAATGTTCTTATAATAATAACATTAATATGCTTTATCATAGGATTAATGTATATTTTGGTATTTCATTTAAAAGATAAATATTTACAGCAACTTGCGTTTATTTTACTAGTTGTCTAAGTATCATGGATTTGATATCCTCTAACCACTGCTCTTTGTATTTCATATCAAGAATTGTTGTGAATGATTGAGTACGTTCTATTTCACTCATCTTCCCAACCAATAAGTAGGCCACAGATTCAACACATAAGAAACCCATACCATCACTAAGAGACCACACATAAACTCTATCAGCTGTTTCTGGTGCCAATTTAAATTGTAAAATAGCACTGCTGAGTAAACCTTTAAGTTTCTCAAATAAAAACCTATCAGACAATAAAAGGACCTCCAGATTTGTTTCTAATTTATCAGCCATCGGGAACACTTCGACATCATATTTAGGGTTATTCAATCCACATTGTAGCAACATTAAAAGGTATTCCAAAGCAGGTTTTGTAACATTTCTCAGTCTCACGCATTTTTCTATGGATTCCCTAAAACACCCTTGTAACATGGCACTAAATACATCAGAGTTTTGACATAAAAATACTCTATTAGCTTTCACTGTTGACGAATCATCTAATTCGAATGTTACCACGTCATCATTAGATAAATTATCCAAAATAGGATCATAGGTAACGTTTATAGTTTCTTTAAATCTATTTTCAAGCGTTTTTGGATCTTTAATGTGTACATTATTCGCTAATTTCATAAGACCAGTTACACAATCTTTTATATCTTCTTTGGATTCAGCGATTATCTTAAATAGTAGATCTAAAGCATTGCAGTCTATAAAGTAGATTTTTAAAGGTTTCTCGGTCCTTACTATATATGGCAATGTCAGTGACAAAGTTTGTTTCATACAATCAGTACCCTTCAAAAGTTGATAACTGATTTCTCCAATGCCATAACTTGATTCTGCTAGGATTGTTAACTGCGTTAATAAATTCATACTGAGCCTTACAACTTGTTTACACTGTTTACATTCTGCTGGTGGATGTTTTGAATTAACAGACATTTGATGTAACCTCAGAGCCAGTCTGTGCTTTAGTATATTCTTTAAACAGTTAGGATTACTTAATACTCTTGCCAAAATACGTGCGACTCTTCCCCAGGGCCTTTTACACCTTTCTACATAATCAAGCAATGCGTTCAAGCATTCTTTTCCAGTAAGCAATTCCAGTGCGTGATTTGGTACAGGTTCATCGCTCAGAGTGCGCAATGTGTTACACGCTCCGTGTGAAACTCTGAATAACAACACTAGAATGCATGCTATACGGGAGTATTTTATGTTTGTCTGGACTTTATCATTGTTTCCTTCAGCAGGAATTTCTTCAACACTGTCATCATCTTCCATAATAAGGTCATCCAATTCATGCACAACGTGCGCTGTATCTAGCTCTGTTACGTTTTTATTTCTCATTTCCATATCTGTTGATTCGCCTTCAGTTTCACTGCAGATGGGTGAATATCGTCCAGAGATTTCTGAATCAGAACTATCTGTAAAAACAATTGTTATTTTTTTTCTTTTCTTCTTATCTGCAGACTCTTGGTCCGCACCTTTTTTTTGCTATCGTTTTATTTATTGTGTAAGCTAAAAATCGTATCTGTGATCAACTATTGGGTGTTTTTTTTTTAAATTATTATTTATGTATTATTATTTTATATATTTATTTTTATGATTTCATTTAACTATTTTATGACGGATTTTTTTTAAAG |
| *Shroud* | | AAAATGTATTTTATTACTTATTCCTAGACACCTGCAACAATTGTTTTCACACAACTCTAAAAAGTACAGTACATTCTTATTATATGTAAAGATAGAACATAGATATTTTCTTTACAAGACACGAAAATGTTTGTATTAATATTTAATAAACAAATAAATAAATAAAAAAACAGTAGTTATAAAGTATTTTAAGTAGATCACTTTATGATTATATTTTATTTACTTAAAGCTTATATAATGCTAAAAAATCATGTTTCATACTAATGTGATTTGTTAAAACAATCATTTAAGGAATACTAAGATTATATATATTTTTAAATAATTGTATTGTTATGCCATATTAACATTAGTTTCAAATACTTGACAATTTTCTTGGTTTCTATTTAAGCTTCTGGAAAACGTAGGAACTTCTTTATTAACCAAATGCGAATAAACTCAGGAAAGGGTAACTTAAATAAGTTGTAATAAAACATATATCTCCAAGATTCAACTTTATACAGTTCTTTGGGATCTTTGTCCAATAAAGCTTTCATGAATGTTTCAATTATGTTCGCATCTGTCATAGAATCAAATTTACATTCATGAGTGGAGGCCAACTGTAAGTAGTTATTAAGTGTTGTTATTTTTTTTCTCGTAGAATGATTTTTGTTCTTCACTTAAATGCCCTATCATAGCTTCTCCATGTAGTCCTTGGCTTGAAATAATGTTACTGGAACTAACAAAACCACCTGGCACAAAAGCAACTACGCTTAAACCATGTTCTCTATGTTCAAGTCGTAAACACCGCGTCCAGGCTAGCAGACCAGCTTTGCTCGCACTGTAAGCGCCAAAACCGGGTAAAGGCTGTAAGCCGCAGTGGCTGCTCACATTAATTATTCGAACTTTTGTATCATTTTTGTTATTAAAAGCACTCTGTCGAATGTCTGGTAAGAAGGCAGACGCTACTCTCATAGCGCCAATCAAATTAACGTTTATTGTGTTTTCTATCATCGCTGTTGTTTGCCATTCATAATTGCCAATGGTCATCACGCCCGCATTATTTACGACAGCGTGAAGTTTGTAATCTGGGTTATCTTTTAAGAGACGGTTCACATAATCTCGGAATCCGGCAACGCTTTTCGGATCGTTGACATTTAAAGGGCAAGGATGAGCGCAAAGTTTCCTTAAGCCATCAGCCGCTTTTGTGTCAGTTCCTTTGTACATCCCAGCCACTGTGACGAGACCCTCCCGGGCACAGCGTGCTGCGATTGCCCAACCAAGGCCACTATCACATCCCGTTATTGCTACTACACGAGTCAGTGACATTCTATTGCGTTCTTTGTTTTCTGCTGGCACCGTCACAGTCCAACTTACCTTATTCAAGCAAGAATTTTTATTTTATCTTAAAATAAACCACAGTCTAAATTATTGCGAAGTCAGAAAAGAACTGACGGTCGAGGGCGATATTCTCCGTCCTGTTCCATATGAAACGAGTTTCGGGTTTCTTTG |
| *Cyp6u1* | | AGCGCCGCGCACTGCGCTAGCCCGCGGCTATGATGTAGCGACCACAGAGTTATATTATATAAGTAGCGAGTAGCGATATGTTTCATTAGAGAACTGACCGGGGCCACGATCACCGGTGTCGCGGTGCTTTCGGCAGTTCGCCGCACATCGATGCCCATGCTTGCGCTCCGGCTTGTTTACACTTATTCAATGTGCCAGTATGCTAAGCGACTCGCGACGGTTTGTTATAAATATCGATTGTTTTTAAACCACCCATTCTATTAAAACTTAAAAAACACTCGTGTGAAAATGTGGATTAGTGTTATTTTATTCGTGTTTTTACTATTTTTAATACTTCTATTAGCGATTTTCCATTACTTTACTGATGGAAGAAAGTATTGGCTATTTAGAAAAGTGCCGTATAGAGAACCGTGTCCATTATTCGGAAATTTTGGAGCAACATTCACAATGAGAAGGAGTTATACAAAAATGTTGGAGTTTTTTTACAATAATTATAGACATGAGAAGTATGTTGGAATTTTTCAAGCTCGCAGACCGACCTTGATGCTGATAGATCTGGATATAGTAAAAACGGTTTTTTCAAAAGATTTCCAATACTTCAGTGACCGTTTGTCTGTATCGACGGATACGCGCAGAGAACCATTGCTGCGAAATTTAGCGAATATGAGCGGATCAGAGTGGAAGGCGATGAGACAGATTATAACACCAACCTTCTCGTCAGCTAAAATGAAAGCAATGTTTCCTTTAATATCTGATTGTGCACAAACGTTAAGAAAAACACTGCTCGAGGAATCTTTGGATGAAGTTGATGTACCAAATATTATGAGTCGATTTACCACTGATGTTATAGGTAGCTGCGCCTTTGGCGTTGATCCAGGTTCATTGAAAAATGCAGAATCACCATTCTTAAAGATGTCAAAAAAAATGTTTAAGATTGATCGTTCGACTGTGCTAAAACGCTACTGTCGTACATTCTTCCCTAGATTATTCAAATTCTTGAATCTCAAATCCTACTCTCAGGACGTGGAGTCTTTCTTTACCAATATAATTAATCAAGTACTTAATAATAGACGAACCACCGGCTACCAGAGACATGATTTCCTTCAACTCATGTTGAACGTACAAAAAACAGAATCAAGTTTTACGATGACGGATGCGTTAATAACATCTAATTCATTTATATTCATGTTAGCTGGATTAGAATCAACGGCCACTACGTTATCCTTTTGTTTATACGAATTGGCAAAAGACAAAGAAATTCAAGATACGTTGAGAAAGGAGATAGCAGAATCTATAAAAAATTATGGCGGATTAAACTATGAGTCTGTGTGTGCGATGCGTTTAGTGACTCAAGCTGTACTTGAAACTTTAAGATTACATCCGCCAACACCTTTAACTACTCGACAGTGTACTGCACCGTGTGTGCTTAATGGTACAGATTTGACGATGAAAACCAAAGATCCTGTCCTCATACCACTGTATTGTATTCAAAGGGATCCACGTAATTTTTCTAATCCAGATAAGTTTGATCCAGAACGGTTCAAAGGTGATCTCAATCCACCTGGATTTGTTGCCTTCGGAGATGGACCTCGAAGTTGCCCAGGAGTCCGATTTGCTCAGCTCACTATAGTAACTGGTTTAGCAGCCATATTGAGTACATTTGAAGTGGAACCGTGCAGTCGAACAACGTCAACCATTGAGTATGATCCTCGTAGTGTGATGCTTAAAAATAGAGGTGGAATTTGGCTTAAATTACTTTCACTGACGAACTGAAAATGAAATGTTTGTTAAGTATCTTAAAAATATAATAAAATAACAGATGT |
| *Torso-like* | | ACGACGCGGCCATGCCGAATACTTTGGGAACCATGTTCTTTAACGTGTGAAACGAAATGTTCAGCTTAATTTTACTTATCTTCTGCTGTGGATTGTGCGTCAGTGAAGAAAGTGACCTCGGTTATGGACTGAACATTGGAAATGCCATCGACGTATTCGCTAACTATGGTGACTTATCACAAGTAACGCAAGTCGTATCGGCTGACTATGAAGGCGAAGGTACAAAGGATCCTATAGTGCCGTTCAGTGAAAAGAACATAAAGGTATTCATGAACGTCTCCAGCAAGGTCTCCTTGGGCGAAGGTATCACGAACATGGAGGTGCTTCTATGTGAAACTTTCGAAGATCTACTTAATATATATTTCCAAAATTTTAACATTGAAGGCACAGATAAACCATGGAAGGCCTTCATGGGTGATTGGATACAGGACGAAATCATGCGGACGTTTGGCATCGAGTATGACACGAAGCCGGACAACTGCTGCTACGTTCTTGTTAAGCTGACAAAGAAATATCGATCAGTGGAGCTTGATGATTTGCGAGACGTTACCGTAAAAGGCTACGTCACCAGAGCCATTGAATCACTCAACGTTACAGATCCTAGCGAAGTGAGACGGTTTATGAAAAGTTACGGAACTCACTATATTAATTCATTTGTCACTGGCAATTTTATTTACCAGGTATTTAAGTACAAGAGAGCAGGGTATAACATGCTTCGTACTTACATAAGGATGCGAAATAGTCGTCCTTCGATACCAGACAACCTGAGGTTCTATTTTTCATCGTACTTCCTAAAGCAAGTTGGTGATATAAGAATCGCAAGTGGAAACAAAACCGTAGAGACATGGGCACGGCGGAATCTTCGAGACAGCCAATACTTATATTCACGACCAAGCCTTCTAAGACTTCATTACAATCCTATTCTCGTTTACAAACTGAACGATCTTCTAGACAACGGTGCGCTACTCGGTCTTGGATTGAAAACCTTACGACCAATGTTCCGCGATAAAGATAAAGCAGAGAAATATGCTGAAATAATAGAAAACGATTTACAGTTGTGGGAAGTGAACGCCTAGTGAAATATTTCTTACAGAAGTACATAGTTTATGTAATTTTTTAAAGATTTTAATTTACCAACTTAGCATTAATGAAGAAAATGAGACCAAATCTTTTCTTTATAGTACCACCATTAAAATTGAATTGAAATGATAACTGTACGTCGATCTGCAAATCTTTTCTTTACAGTACTACTACCATTTAAATTG |
| **juvenile hormone related genes** | | |
| *Farnesyl diphosphate phosphatase* | | ATTGGCGTGTTCTAAAAGAACAAAAGTTAAGGAAATAACAAATTAAATATAGAAAATTCTTTTTGCTTTAATTTCAAATACTTTATTTTTTCTAGCTTAAAAGACACGTTATTTATTTCAAAAAAAGTTGACAACCCTAAGTATAGCGTATATCGGAAGTCGAGAGAGGAATAAATTAAAGGACAATGAAACCACCGGCTAATATTTTTCACTAATTCTGGACAAACATTTCAGTTATTTCTTACGAATCTATATTATTTCCTCATACGTGTATATTTCCTTGAGATATTTAACAATGTTTATAATAATATTGAAGTAAATATTTATTTTCCCTATGTCTTCGTTTACACGATAGCAGTTTAAATACGGCTTCTAATTAAATAAATAAAAATTGTACTACAACAATTACATTTACATACAAATTTCAGTTTAAAGCAATGGTACTAAACTTCCTAAAGATGGAGCATAATAGTCTGGTTTTATGGTTTTGTGCGACAACATCTCTTGTTCCGTGAAGCTTGTCAGTACTAATAATGTTTTGAATCCAGTCGCCTTGCCCAGGCCGATGTCCTGTTCGATCATATCACCGATAAAAAGCACGCGGCTCGGATCTGTAACCTTAGCTCGTTTCATAGCTAACTCTCCAAACATTTTACCAGGTTTACCCAATTGTATCGGAGTGCGATTCACATGTTCCCTGACTACATCTGTAAACGCTCCTGTGGCTAAAGCTAAATTGCCGGCATAAGGCACCAGCCTATCAGTGGCACCATTTATGAATAGCACCTCAGGTCGCTTCAAGTACGTTGACGCTCTGTGTAATTTCGCCAGGTTTATACGAAAATCGCTGTCAAAAACGACCGCTCCAATCTCTTCATCATCTATGAGATATTGAGCGTAGTCGGCGTAATATTCAGGGCCTAAAGTTGGTCCATGTTCGCATTGGAAGCCACTTGCTTCCAAAATTCTTATAGTTTCGGGACACGCCACACAATATACTTTCTTGTCGAAATTTACAGATTTCAAGTACTCCGCTATAGCGTTAGAAGGTATTGTCAAATTTTCAAATCCATTTTCAATTCCGGAAGCTTGAAAGAATCCTTCGTAGTTCTCTTTAGTTCTCAGACTGTTGTTCGATACAAAGCTTACAGTTTTACCGTGATCTCTCATTAATTGCACAAAGTCACCTGACCCTGGTAGTGGAGTTTTGTTCCAGATTACCCCGTCACAATCCGAGAACACATGGTCGAAAGTATCGAGAAACTTTTTAAATTCATTCGAATTTAACCCTAAAAAGTTCACAGGTTCGGAGCTCATATCGATTTAATCACAAAACACTTATATCGATATATTTAAACGATGAACACGTGTACACCTAACCTAGTTG |
| *Aldehyde dehydrogenase (NAD+)* | | TACCTTAAAATAGATTTATTATAAACAACAACAAATTTTACGATTATCCCATTACAATATTTAAAATCTTCTTATGATAACTTAATAATTAAAATTTCTTCGGTAAGGCAATCGTTACAGTTTTCACTTCTAAATACGAATTGACACTATCGAAACCATATTCACGACCAATACCGGAATCCTTAAAACCGCCGAAGGGAGCCTGTACACCCATTTGTAAATACGTATTCACCCAAACAGTTCCAGCTTCGACATGTTTACTAAACTGTAAGGCGTTATTCACATTATTAGTAAAAATGCCGGCAGCGAGACCATAATTCGTAGCGTTGGCACGTTCAATCACTTCCTCTAATGTGTCAAACTTCAAGATACTCTGAACTGGGCCAAAAATCTCTTCTTTAGCTATGGTCATATCGTCTGAGACATCTTTGAATACAGTAGGCTCTATGTAGTAACCAGTTTTGCCAACACGTTTCCCGCCCGCCATTAATTTTGCTCCCTCCTTGACGCCTTTCTCAATATATCCTAATACCTTCGTCATCATTTCCTCATCCACCTGAGGTCCGTGTTGGGTGTTGGGGTCAGATGGGTTTCCAATTTTGATACCTTTCGCATATTTCGCGGCCGCTTCAACGAATTTATCGTAGATACCAGATTGTACGTACAAACGTGACGCAGCGACACAAATTTGCCCTTGTAGTGTAAATACAGCGCTTGCCGCGAATGGTACAGCCATATTTAAATCAGCATCGTCCATAATGATTAGAGGACTCTTTCCACCGAGTTCAAGGGTCACTCGTTTGAGATTATTAACTCCGGCTGTTTGTTGTATTATCTTGCCGACCTCAAGAGAACCAGTAAATGATATTTTGGCAACATCTGAGTGGTGTGTTAAAGCTGCACCAGCTGTAGGCCCATAACCATTAATAACGTTCACAACTCCTTTAGGGAATCCAGCTTCTTTTATAAGTGCTGCTAAAACTAACGCCGTGAGTGGTGTTTGTTCAGCTGGTTTTACCACTACTGTACAACCTGCGGCTAGAGCTGTGGCGACTTTGCCAAGAAAAATATAAACAGGACCGTTCCACGGTAGTATAAGACCACAAACTCCAACGGGTTGTTTTAAGGTGTATGTAAATACATCACCATCTGCTGGTATAGTATCTCCTTGAATTTTATCAGCGATTCCAGCGAGATATCGGACAACTACGGCTGATTGTTTTATAAATCCCACAACAGTAGTGAGTAGAGCTCCATTATTATAAGATTCAAGCTCGCCAATATACTGGGCATCACGTTCTATGAGATCGGCGAATTTGTTAAGCAATTGGCCGCGTTGTGAGGCATCTAACTGACGCCATTCTGAATTACGATGGAACGCTCGTTTTGCTGCACTCACTGCTAAATCAACGTCAGCCTTATCCCCTTCGGCGACCTTGGATATGACGGAACCATCGTGAGGACACCGAGCTTCGAATGTCTTTCCACTTTGTGCGTCGACCCATTCGTTGTCTATGAACAACTTAGTGTATTTGATTATCGGAGCCATTGTGATCAGAATGCGACCG |
| *Juvenile hormone acid methyltransferase* | | CAAGTGCGTACAGAAAGTACATGTTTTTGACAGTGAAGATATTCTAAAAATGACTATGAAATCGATAAATCCGTTCAAAATTCCGGAAGAAAGGATGGATGAATTTCTGGACGATCTCATCGATGTAGTTCGTGATATGAGATTGGTGAACAATACCAATAAAACAGTGAAATTCAATTATAATTTAATTGTTATACATTGTACAAAAC |
| *Juvenile hormone epoxide hydrolase* | | AGTACATGGAAGTAACATACGTTGTAGATTGTAGTCTACACATTTAGATAATATATTCAGTTGCTCAGTGTGTAACAGAACGCGCGTAAATGTAGACGCTTGCTATATAAGCTGGAACACAATATATTCGTATGGCAAAATTAATAGCAGTGTTGGCCGTCGTGGTCATTGGTGTCGGGATCTGGTATATGCTGTCATGTGGTGAATCCAGACCTCCCATCCTCAATCCGAACGAATGGTGGGGACCGAATGAGCTGAAGACTAAAAATGATCAGAGTATACGGCCTTTTAAGTTGAAGTTTGACGAGGCAATGATCAACGATCTGAAACGTCGATTGAAGAATCATCGTCCCTTTACGCCTCCGTTAGAGGGCGTTGCTTTCGAATATGGCTTCAACACCGTAAATATGGACAGCTGGCTCAAGTACTGGGCTGAGGAGTATCCGTTTCAGGAACGTGAGAAATTCCTGAACAAGTTCCCTCAATTCAAGACTAGTATCAAAGGCTTGGACATTCACTTCATCAGGGTTCGGCCGCAGGTACCTTCAGGAGTTCAGGTAGTGCCACTTCTACTAATGCATGGCTGGCCTGGCTCAGTCAGGGAGTTTTATGAAGCGATACCGCTCCTCACTCAGCAAACGCCAGGATATGACTTCGTATTCGAAGTTGTTGTACCTAGTTTACCTGGATATGGATTTTCCGATGCGGCAGTTCGTCCGGGACTCTCCATTCCTCATGTGGCTGATATCTTCAGGACCCTAATGCAAAGACTTGGACATAAGAAGTTTTACATCCAAGGTGGTGACTGGGGTGCCGCTATTGCATCTGGAATGGCCACTCTATTCCCCGATGATATTTTAGGTCTTCATAGTAATGCAGCTGTTACTCAGCATCCGTGCGCGATGATGAAAACTACGGTGGGTGCGTTCATACCATCTCTGGTAGTGGAAAGTCATCTTGCAGATAGAATGTATCCGCTGTCATCATTTTTCTCGTATCTTTTGGAGGAGTTTGGCTACTTCCACCTACAAGCTACTAAACCTGATACGATTGGCGTATCACTAACAGATTCCCCATCAGGACTCCTCGCGTACATTCTGGAGAAGTTTGCTACGTGGACACGAAAGGAACATAAATTTAAAGCAGATGGAGGCTTGAGTTTCAGATTCTCAAAGGAGAAACTGATAGATAACCTTATGATCTATTGGAGTACCAACTCAATTACAACCTCAATGAGGTTCTACGCTGAAAATGCGAGTAAAAAGACTAGAGATTTGCAATTGGATACGTACAAAACCCCAGTGCCGACGTGGGCTTTCCAAGCAAAACATGAGCTGTTTTACCAGCCGCCATCAATTCTTAAAACGAAATTCACAAATCTTTTAAACACGACAGTCATCGAAGACGGTGGCCATTTCCTCGCGTTGGAACTACCAGAAATATTTGCCGAAGATGTCTTTAGAGCCGTGAAGGCTTTTAGAGAATGGCACAAACGGAGTACTAAGACTGAATTGTGATTTATAAGACGAGCAATTTAAATAGGGTGTAATAAAGGGTTTTTTCACATTAAAACAACAA |
| *Juvenile hormone esterase* | | AGACGTCGTCGATGATATCTCAAAGAGAATCATCCAAAGATACTTCAACGGCACTCTGAATATAGAAAAGTATTTAAATCTATGTTCTGACTCGTTTTTCATGTACCCTGCATTACCATTAGCAGAGAAGAGCGCTGAAGCTGGTATAGCTCCCGTATTTCTTTATAAGTTCTCGTACGACGGTGATAATAGTGTATTTAAAAAAGTCGACGATTTGAAATTTGAAGGTGCTGGACATATCGAAGATTTAACTTACGTGTTTAGAGTAAATTCAATACTTGAAAAGCACATTTCTTTTCCACCGAGAAACCGGGATGACCATATGAAATACTGGATGACTATGTTTGTCACTAATTTTATGAAATGCAGTGACCCGGTCTGCTCAGACAACTCCTCATGGCCAGCGGTGAATCCGAATAAGTTAGGCTATCAACACATACAGACACCGAATGTGTACCAATACACGGAACTAACGAAAGACGAAATGTCCATGAAACACTTCTTCGACAGCTTCCAGAAGGATAACTACGTGAATGGTGTCAAATAGAATTACGAAGAGAGCCTCGTGTGGTCTATAATATAATAGTGCTGCTGTGGTTAATTT |
| *Cytosolic juvenile hormone binding protein* | | TGACCTTTCAAGTTCCAGATGACATTTACCACTGTAACTTATGTAGTGCTTTGGTGTAGGTAGTGTATGCGTTTATCCACAATTTTTAAATAAGTGAAAGCTTTCTTAATTTTATTATTTACTTTGTAACATTCTGTAATCTCAACATACATATAACAAGATGAAGATTAACGGACGCGCCCTTCATTTCGTATTCAAAGTTGCTGATCGAACTCTGACGGCAAAATTTTACCGGGATGTACTAGGCATGAAGGTTCTTCGTCATGAAGAATTTACTGAAGGTTGCGAGGCAACTTGTAATGGGCCTTATGCAAACAGATGGAGTAAAACCATGGTTGGTTACGGTCCAGAAGATTCACATTTTGTTGTTGAGCTGACATATAACTATGGAGTAACACACTACGATCAAGGAAATGATTTTCTTGGCCTCACACTCGAGTCTAGTGAGAGTTTGAAAAGAGCTGCTGCAAACAACTGGCCAATTCAGGAACAAAATGGTTTGAAATATGTAGAAGCACCTGGCGGTTACAAGTTCTACATAATTGATAAACCACAGCCTGTTGACAGAGATCCTGTCGTAAAGGTGTCGTTAGCTAGTTCAAATTTGTCGAGATCAATCGCATACTGGAACGGTCTGCTCGCTCTGAAGATATATCAACAAACAGACAAGACCGTCCTACTTGGTTTCGCTGACAATCAAGCCAAACTCGAATTAGTAGATATTGGTGGCCCCGTTGATCATGCAAAAGCGTACGGTCGGATCGCTTTCTCTTGTCCGTTCGATGAGCAGCCAGCTATAGATAAGAATATCCAGGAAGCTAAACAGACTATCCTAACCCCGCTGATCTCTCTCGATACTCCCGGGAAAGCTACAGTCAGGGTTATAATCCTAGCTGACCCCGATGGTCACGAGATCTGCTTTGTCGATGATGAATCATTCCAGCAACTGTCTCAAGTGGATCCCGCAAGCGATGCCGAGTTGGACAAGTACATTAAGGCAGACAAATCTCGAGCCTAAAATTGCTTATTGTTGCAGAAATTAAAATAAGTATAAAAAAATGTGATGTCATAAAACATTCCCGGTGTACGTTTTTTTTAAATTGTTATATAAATTTAAAGTCTATTATTGGTTCTATATATACTTAAGTGATTTGTTCACTAACTATTGTGTACTTATATTTGCAAATAAATTTTAAATTGAATAGTTA |
| *Juvenile hormone binding protein* | | ATTCCGCGTGTATGATTGCCGCTAGTCCAGTGTGAACATTCAGAACAATACTTAATCATTTATAAACTCTACAAAAATGGATATGACTTTGAAGACAATTTCGTGTTTTATATTGCTTTTAATCGGTTGTGTTAGGTCATTCGAGTTGCCAGTGGGAAAATGTAATCTCGAAAATTCCGCGTGTATGATTGCCGCTTTCCAAAAATTCTTGCCAGATTATTTTGGTGGAATACCAGAGCTAGGTATTGAAGTTCAGGATACTTTGGTAATGGACGATATTAAATTTGATTTGGCTGGCCTTCAGTTCTTTTTGTCTGGTGGTCTGCTTAGCGGACTGAAGAATAGTGTAATAGACGACGTAAAGTGGAATGTAGCAAAGAAATTCTTTAAGGTTATTTTTCATTTCGACGGTACTGTAACAGGTCATTATACTGCCGCAGGTAAGGTAATCATCTTACCCATAACAGGAGACGGGGAGATGACGTTAAAAGCAAAAAAATTTGCAATTGACATTGGTAATACCTTACGAAATAAAAAAAGGAAAAGATAATAAGGATTATATTGCACCCAAAAAGATTGATTTTGAGTATAAAGTGAAAGACAATACACATTTCGATTTGACAAATCTGTTTAATGGGGACAAAGAGCTCAGTGATGCAATGCTGAGATTTTTAAATGAAAATTGGCAAGAAGTTTCAACAGAATTTGGTAGACCAATAATCGAAGGCGCCTTTAAGAAGGTCTTGAAGAATTTAGTTACATTCTTCCGAAAACAAGCGATTACAGATATAGCCGATATCTGAACTTCTATAGAGTATGCATAGCTGGTGTAGGTATTTTGCATTCTTCTGCAAACACATACGTTATCATAGAAAACTTGGTCATTGATTTTTATTGGGTTTAATATATATTAATTGTTAGTCATAAGATTTCATTCTATTTCACGAATATGAATAAAATTGTTGGTACTGTGATATAGTCT |
| *Juvenile hormone esterase binding protein* | | ATGGTATAGAACAAACTGTTTTTACCATAAGAAATTGTTTTTCATGTTTTGTTTAATATTTTCTTTTAAAAAATCTCAATATTTTTACATAAACAAATAGTGAAATATGAATTTAGTTTTAAGGCAAGCCCTAACAAGGCATACGTTCCGATTATGTGACAGAATCGCGCACAAAAATGTATCAAAACAAATCGTCCTAACCTCATCCTGCACTGTTATACAATATCGTAAATATTCTGAAAATGAAGTGGAATCACGTAAATTGCCGCAACTAATGGATTTCCCGCCAATAGTATGGCCTTCGTTTATAAAATTTCTCAAAAATTGGATGTTTTCTAATTTTATAATAAGACCTTACTTTGATCAAGACTTCAGGCTCAATGAATTTATCGAGGCATCAAAACATGCTGTTCAGGTTGTTTCAGATGCATTACAGAGAAGTGATTTCAAAGCCCTCGAAGATCTGGTGGATAAGGATGCTATTGCATCACTAAAGACTGCCGTGTCGCAGTTATCAGTGTCACAAAGGCAACTTCTTTCCATTGACAAGGAGGACATATTCTATGCTTTCCCTTATCAGGTTGGAGTAATGTTTGATGAATCAGAAAAGCGATGGGTTGAAATCACAATGTGCTACCATGTTCTAAGGGGATTAAAACGTTTGAAGGAGACTGGTGATCTACCTCCTGTCTCATTAGGAGTACAACCTGAATATCAAGATAATATATTCATATTGAACTACCGATTTATAAGAGAGTACACTAAAGGAGTGGAAGACAGTTGGTGGGTGAACATAGTCAATCACTTCCAACCGCAAACGGTTGTAAAGAAATTTTAAAAGTATATAATGTAGTCTATGATGATATGACTGATTAAAAGAAATGGCTAAGTTCAGTGTTTAATAAAATACAATTGTTTTA |
| *Hexamerin* | | ACTCTATCCGGTCGGCTGCAAGATGAGGGCTGCTGTGTTCTTGGTTTTAGCTCTGGCCGTCATGGCCTCAGCACGTCCTGATATCGATGATAACTTCTTCGTATCAATGGACATGAAACAAAAGCAACTGTTTATTCTAAAACTGTTGAACCACGTCACCCAGCCCGTTATGTATAAGGAAATCGAAGAAATCGGCCAGAATTTTAAATTTGAAGATAACATGGAGCTATTCACTAAAACAGACGTTGTCAAAAGCTTTGTAAACACGCTAAAAGTTGGCGTACTGCCCAAAGGGGAAGTGTTTACTCTCAACGTCGACCGTCAAATGAAGGAGGTAGTGAATATGTTCCATCTGCTCTACTACGCAAAGGACTTCTCTACATTCCTGAAAACCGCCTGTTGGATGCGTCTATACCTTAACGAAGGCATGTTTGTGTACTCCCTGTCGGTCGCTGTCAGATACCGCGAAGACTGCAAAGGTATCGTTCTTCCTCCTCCGTACGAAATTTATCCTTACTACTTCGTTCGCGCCGATGTCATCCAAAAGGCTTACTTAATCAAGATGAGAAAAGGCCTTCTCGACGTAAAATTGTGCGATTTCTATGGCATTAAGAAGACTGAGAAAGATGTATATATTATCGATGAAAACGTATTTGACCAGCGTGTTGCTATTAATGATGAAGAGAGGATAAAATATTTCACTGACGATATCGATTTGAACACTTATTACTATTACTTCCACGTCGACTATCCGTTCTGGATGAAGGACCAACTTTTCAGTAAATACAAGGTCAGACGTTACGAGCTCACCTTGTACATGTATCAGCAGATACTCGCCAGATACTACTTGGAGCGTTTGTCTAATGGTTTAAATAGTATCGGCGAGTTTTCATTTTATAAACCTATCCAGAAGGGATATTGGCCATGGTTGATGCTTCACAATGGTATCGAGCTACCTGCCAGGTTTAACAATTACGTTATATCCAGAAATGACAATAATGAGCTCATTAGTCTTGTAGAAGAGTATGAATCTATTATCAAGGAAGCCATAATGAAAGGATATATTGACATGAACGGTTTAAGACTTGAGCTAACTAAGCCAGAGGATATGGAAACCCTCGGTAAACTGATTTACGGAAAAGTTGTAGCGAATGAGCAGGAAAATAAATGCATCGAGGCTTACCGCTACTTGCTTATTGTCATGAAATCTGCTATGGGTCTTAACACTTTCGTATCTGACAAGTACTTCGTGGCTCCTAACGTTTTGGATTCCTACCAGACAGCGCTACGTGATCCAGTTTTCTACCAGTTGCAAAAACGTCTTATCAATATCGTTCAGTTGTTTAAAAAACGCCTTCCCTGCTACACTAAGGAGGAACTCTACTTCCCTGGAGTTAATATTGACAGCGTCCTCGTCGACAAACTCGTCACTTACTTCGAAGATTATTTAATGGATATGACTAACGCTGTTACACTTAATGAAGAAGAACTGAAGAAAGCCTCATCTAGTATGACTTTCTTAGTGAGAAAGCGTCGCCTGAATCACGAACCATTCAAAGTGTCTCTTGTAGTCCTATCCGATAAAACTGTCGACTGCGTTGTGAGATTGTTCTTGGGACCAAAAGAAGACGAATTTGGACGTCTTATTGACATCAATGTGAATCGTCTAAACTTCGTTGAGATTGACTCCTTCGTCTACAAGCTAACCACCGGCAAGAATACGATTGTCAGGAAATCAAGTGAAATGCATAATTTAATTCCTGATCGAATGATGACTGCTGATTTCATGAAGAAGGTTGAGTCTAGCAGCGATGTGAGTGAACTGTTCGTTAAAAATATGAAGAATTACTTCACAGGGTTCCCCTCGAGACTGATACTTCCCAAAGGTAGAACCTCTGGCTTACCGATGATGATATACGTGGTAGTGTCACCTCTGAAGCCTGTTGATAATATCGACGTGACTGCAATGTTGGAAAAGAAGAGTTTCTCTTTTGAATTCAGATCCACTGTATTAGTCGACAAGATGCCTCTGGGTTACCCTCTAGATCGTTATATTGACACCTCAAACTTCTTCACAGCGAACATGAAATTTGTGGACGTAATGATCTTCCATAAGAAACAAACGTGTGACATGAAGATGCGTTGGAATAAATGGGTACTGAGGGACTACAACATGGCGAACAGATCTCCAATGGAATCTGATACATACTTCGTCGAGACAGATTTGAACTTAACTACGAGGGTCGAAAGCAGTGCTAATATCATCGATATATAAGCTAACAAGATAATGTGTAATTATGTAAGTGACCGGATAAAATGAAAAATATTTATGACCAATGGCGTTATTAAAAGCATCGTAAAAATGTAAAAAATAAAAGACATTTTGTTTATTTTAAAAAAAAAAAA |
| *Broad* | | GGAATGGAGGTCATGTCATGCATTTTAGTGCATTTTAGTTAAATTGATTAGGTACCTATAACATTTTATTGCAAGTGTTAAGTGATGTTTTATATTTTTGTAATGCATGTTTCAGTTAAGATTTTTCACAAAATTTTGTAGGGATGGCCGACCAATTTTGTTTACGTTGGAATAATTTTCAGTCTAATATTGTCAGTGCTTTAGATTCTTTGAAATGTTCGGAAGATTTAGTCGATGTGACACTCACTTGTGAAGGCAGAAATATCAAGGCCCATAAAGTCATACTGTCAGCGTGCAGTCCGTACTTCAGGAATGTGTTCAAGGAAAATCCATGTCAGCATCCTGTTATAATCCTGAAAGATGTTTCGGCTGATGATATAGTGAGTCTGTTGTCATATATGTACCAAGGAGAGGTGTTTATTGAAGAGAACAAATTATCATCTTTTTTGCACACTGCTGCCTTATTGCAAGTGAAAGGCCTCACCGGTGTTACCCAACAGAAAGACAATTTCACATCGCCAAACACGTCAAATAAATTGTACACCCAGCTCACAATATCATCAAAGTCACTATTCAATGCCTCTCACAAAGAAACAAAGCCACCAGCGTTAAAGAAACGACGAAGTAGTACTTCTGATAAACCAAGTGATGTTACTATTGGTGACGGTCATAAGAAGAGTAAAATCCTAGAAACATGTGATATTTGTAATAAAAATAATTTTTGTACTTCTTCAAAAGGTGATAATTTAGCAACAGAAAATAATGTAGACAAGAAATGTGATTTAAAAAGTGATCAATCCATTGTCGCAACCAATGGGAAAAATTTATCTTCTTCTCAGATTAATACTCAAGGAGAAAATATTCCCATAAACATCAAAACTGAAAGGATTGAAGACAACAACTCCCCTACATTATCTGCAACAGTAAATTCGGATAATGATGATAGTCTACCAGATTATGAAAACAGCATGCTTGCCAGATCTTTATTATCAGGTATCAATCCATCAAAAAGTGATATTAGTGCCAACAATTCATGTACAAAAAAAGTTTCAAATGATAATCTCACTACAATACGATCAAAAGACATTAATATTGATAGTTTTTCCACTGCGGCATCTAAAAGTCCAATGAAGCCTAGTAACACTGATGACTTACAGGTGAAACCTGAAAAACAATCTCCCAAATCGGACTTGGACTATGAACCTGAAGTATTGTTATCGGAACACCAGGATGGTACAGACCCAGATAATTCATACACACAGGAACAGTCCCAAACGTTATTATTACTTGCCGGAATGTCGTCTGTTCCAGCTTTAGGGACAGTTGCTTCGACATCGCAAGGACTGTCACACCAGCAATCAAATCATGCTGCGATATGTGGTGATTGTCCACACTGTGGTATGAAATATTCAAACCAGTCCGCTCTTAAGTATCATGTGAGGTTAATGCACTCGGACTTAACTAATAGACTGTGCTGCTATCTATGTCCAAGGTCGTTCACAATGCGCGAAACCTTTAAGGAGCATATGTGGACCAGTCATGGCCAGAGAAATTAATATTAAACCTATGCGTGTACGATATACAAGTATATGATCAATCAGATCAATGTGCCTTAGTTTGGTTCCATATTGTCAATTGAGACAGAAAATTAAGTAGTGATACATCTAATGTATGTGCCTTGTTTGGAAAGTTTTTCTTTTGTATACTAAGAAACTTTCAAAAATTACAACAGATATTAGTTGTAAGAGACTGACCTTAAACTTAAAGAAGATATTAGATAAATAAAAGGTTACCTAAAAATTGCCTTAATGTATTAAAAAATACTTATTTGGTATTTTTTGGTAGATATTCAACGATTACGTAAGCAATTTTAAATTCTCATCTCCTGTTTAGGATACAATAAGAAAATCTTTATATTATTTTTAATATTCATTGTTAGCCTTTAAGAAACCAATTACAGGGCTATAGGTCTTTTCCCGAAGGAAGGTATGACCATAATCACCATATGACATATTGGTGATTTCAAACTCTGAGA |
| *Allatostatin receptor* | | GTAAGCGCGCGGCGCTCGACGTGGTAACGCATGTTTCGAGTTCTCTGCACTTCTTTAAAACTTTATTCGATATACTCCCGTTAGTTACGTGTGTACATAAATTCTAAGTGAATGTTATAAAGTTACAAGTATTAATAAGTTTTGTGTCGTTTAAGAAGATTCACACCATTCTCAAATGGATTGACGATATCGTACATGATATTCAAATTTCAAGCTAATTTGATTCAAGTTGCAGTTTCAACTGAACCCCTGCAAAGCGATACACACGACTGGAACGGCATAAACGACGGGCTTTAAGAAAATAAATGTTTACTAAAATCAAAATGTAAATTCTGTTTCTGAAGCGTCGTTTCTATTCTTGGGATCAAAATGGAACTAGAAGATGTTGAAATGTACGGACATGGGAACTACAGCTATGATGAAAACGGCACATACAACGGAACGTTAGAGAACTGCCCCAACATCAACCTCCCCATCGTGTACGTCGTCACACAAGTGTTATACGCGTTGGTTTGCGTGGTCGGTCTTCTCGGAAATACCCTTGTCATATACGTTGTTCTGCGCTACTCCAAAATGCAAACCGTTACTAATATGTATATCGTCAATTTGGCAATCGCAGATGAGTGCTTTTTAATTGGAATACCTTTCCTGATAGTAACAATGTCACTCCGCGCTTGGCCTTTTGGACGTTTCATGTGCAAGGCTTACATGATATCAACAGGAATAAATCAATTCACCAGCAGTATATTCCTATGCATCATGAGTGCTGATAGATACATCGCAGTTTGCCATCCTATAGCCGCTCCGCGATTGCGAACACCATTTGTGTCTAGGATTGTATCAGCCGCCGCGTGGACAGCATCAGCGCTGGTCATGACACCAATATTCATGTATACGACGCTTATACCTACAGAGAATGGTTTATCCTGCAATTTAGTTTGGCCCAAGCGAGATTTCAACAAAGGACAGACGTCGTTTACTCTTTACTCCTTCGCTTTAGGATTTGCCGCCCCTCTCACTCTTATTTTCATATTTTATTTCTTGGTTATTCGTAAGCTGAAAACGGTTGGACCCAAGAATAAATCCAAGGAGAAGAAACGATCACACAGAAAGGTAACCAAGCTAGTTTTGACCGTGATCGCCGTCTATGTTCTTTGCTGGTTACCATATTGGGCATTTCAAGTGGCATTGATATATTCGCCCCCAAATGAGTGTGCTAGTCGCATAACTATCACTGTTTTCTTGGTCGCTGCTTGCTTCAGTTACAGCAATTCAGCTATGAACCCAATTCTCTACGCTTTCCTCTCTGATAACTTCAAGAAAAGCTTTTTGAAAGCTTGCACATGTGCCGCTGGGAAAGACGTTAATGCTACGTTGCATGTGGAGAACAGTGTTATACCGAGGAAACGAGGCGGAGGAGCGGCCCGAGCACAAGCAAGGTCCATAGAAGCGAGAGGAGGCATGTCAGTGGCCGTTGCAGGATCTCGGTCTGAGGCTTCTACAGCGTTGACATCTCGGTCGGTAGCGGCAAGCGAGTTGTTGCCCTTGGAAGCGCGACCCGCTACGCTTACGCCCCTAATAGCGCCCAACGGCCTGTCTCACACGCGGCTCTGAGTGAGGGCCTCGCGGCGTCGCGGCGACCCATCCCACGTGCTGCTCGAGGAAGACACTCTAATAGTCTCCATAGATTAAACTAAAACTGGACAGTTCCTGACACCGACAGCTGGGAATGATACGAGCCCTATCTTTCCAGCTGTCGCTTGCCGAGTCCACTTTTATACCGTTAATGAATATTTAGAGGTTAGGGGTGGACCAATATGTTCCCGATTCTAGACTAGTGTGTAAGGTACTTTACAAATGAATGCAAACGCTCGGCCTCGAGTTGTAACGATGAATTTATTAAGTGTTAAATATTTTTATCGCATAGCGAACGTAATGCACGCCGTATTATGTATACCAATACTAATATTATTATTTAGGTTTGTATAAATGTACATATCGTGTATATAATCGTAGCATTGTCAATATCTCAAAGTTGTAATTAAATTACTTTAGTCCTTAAAAA |
| **circadian clock related genes** | | |
| *Cryptochrome 1* | | CTGGCATGTAAATAGAAACAAGAAACCTATAAAATGTGAACGAAAATTATTATAATTGGATTTTGTTATTTACTTTTATTTATTTTGTAAAGTAGTTTTAATGTTTTAATTGGAGTTTTGGTATTATATGTGACTTACAATGGTATATTGTTTTGTACATAATGTTTGTTAATTTTTAAAGTGGTTTGTGCGCGAATATTTTTATTCAAGCTAATCTCTGAAACAGTTGAACCATTTTGATAGGACTTTCACTGGCAAATAGCTGATGATATAAGGAATAACATAAGCTTTAATATAACAAACTAAAGAAGCATTGTTCAGAAAAGTGTATACTCTACTTGCATGTGTACACTGAATAATTTTGTTTTGCTAAATTTCACATGGTCTTTGTTGCAGTTTGAAACATCATCTAAGATGCTCGGAGGAAGTGTGCTTTGGTTTCGTCATGGTCTGAGACTACACGATAACCCTTCGCTGCACGCTGCATTGGAAGATAGGAGTGTCCCCTTCTTTCCAATTTTCGTCTTCGATGGAGAGACTGCAGGTACTAAGTTGGTGGGGTACAATCGTATGCGATATCTCCTCGAAGCTTTGGAAGATTTGGACAACCAGTTTAAGAAGTATGGCGGCAAATTGATTATGTTGAAAGGGAAACCGAGTGACGTGTTTCGGAGACTATGGGAGGAGTTTGGTATACGGAAGCTTTGCTTCGAGCAAGATTGCGAGCCGCTGTGGAGGGCTCGTGATGATGGCGTCAAGAGCGCCTGTCGCGAGATTGGGGTCGCTTGTCGGGAATACGTGTCCCACACTTTATGGGAACCTGATACCGTCATTAGAGCCAATGGCGGTATTCCGCCCCTCACCTACCAAATGTTTCTGCATACAGTAACGATCGTAGGAGATCCTCCACGACCCGTCCCCGATGTCGACATGAGTGGCATCACGTTCGGCACCTTGCCTGATTGTTTCTACCAAGAGTTCACGGTTTTCGATAAGACTCCGAAACCGGAGGATTTGGGGGTGTTCCTAGAGAATGAGGATATTCGTATGATCCGTTGGGTGGGCGGAGAGACTGCCGCTCTTAAGCAAATGCAGCAACGTCTCGCTGTGGAACATGAGACCTTTCGCAAAGGTTCATATTTACCGACACACGGCAGCCCGGACCTGCTCGGCCCCCCGATATCGCTGAGCCCAGCATTACGTTTCGGATGCCTCTCTGTTCGAAGCTTCTATTGGTCTGTACAAGATCTCTTCCGTCAAGTGCACC |
| *Cryptochrome 2* | | AGGCGTTAATCGCCAGTATGCCCCCGCCTCAGCCGGCCGAAGCGCCTATATCAATCGAGACATTAAATGGAGCCAAAACTCCAGTTTCCGTCGACCACGACGATCGTTTCGGCGTCCCCACCCTTGAAGAACTTGGCTTTGAAACGGAAGATCTAAAACCACCTATGTGGATGGGTGGGGAAAGTGAAGCATTAGCCAGGCTGGACAGACATTTAGAAAGAAAAGCATGGGTAGCATCATTCGGAAGGCCCAAAATGACTCCACAGTCTTTACTGGCAAGTCAGACGGGATTATCGCCTTATTTGAGGTTTGGGTGTTTATCGACGAGATTGTTCTATTATCAATTAACAGAGTTATATAAAAAGATAAAACGAGTCCGGCCACCTCTTTCTTTGCATGGGCAGATATTGTGGAGGGAATTTTTTTATTGTGCAGCCACACGCAACCCTAATTTTGACCGTATGGAAGGGAACCCTATATGTGTCCAGATACCTTGGGAGAAAAACCAGGAGGCTCTATCGAAATGGGCAAATGGTCAAACTGGATACCCGTGGATTGATGCAATCATGATCCAATTACGAGAAGAAGGTTGGATCCATCACTTGGCTCGGCATGCAGTGGCTTGCTTTCTCACTCGGGGTGATCTCTGGATATCATGGGAAGAAGGCATGAAGGTATTCGATGAACTGATGCTGGATGCTGACTGGTCCGTTAACGCTGGGATGTGGATGTGGCTCTCCTGCTCATCTTTCTTCCAGCAATTCTTCCATTGCTACTGCCCTGTACGATTTGGCAGAAAGACTGACCCCAATGGTGATTTTATCAGGAAGTACATTCCGGCGTTGAAAAATATGCCAACGCGGTACATACATGAGCCGTGGGTCGCACCGGAGTCAGTGCAGCAGGCGGCGCAGTGCGTCGTGGGATGCGACTACCCGCTGCCGATGCTCGACCATACGAAAGCATCACAGATCAACTTGGAACGTATCAAACAAGTCTATGCACAACTCGCTAAATATAAACCACAAGTAACGTTACAAACGCATACTGTACAGCGGCCGAATGTGATGCAATCGTCGCCGAGTCCTACTTCAATAATAGCCAGCATAAATCAATCAAATTTACTATGCAGTACGACACCAGAATCGCAATCCGCAGCCACACAAATGATCTACAAAGACCCTTCTGGTATATTTCAACGGCCAACTAAAAATGCAGTCCACGCGGATACTAAAAACACTAAATTCAAGAAAGTCCTTATAGTGCATCAAGTACAACATTCCGAAATTACAGAAGAAAGGGTATCACAGACAAATAGCCATCACCAGACACCCAAAGAAAACTATATAATTAACAGTCGGCAAAGCATAGAGTATAAAACTTGTTCAGACAATAATCTACAGCCACCGAATAAACATAATAATTATGATTTTAAAAATTTAGTCATAAATAACCATATACAAGGGTACAATAATCACATTATTTATCAATGTCAGAAAGGAAATCAGAATGAAATATACAGCCAACAAGGGGTCAAAACCAATGCTTACGGTTATGAAAATACAAAATTCTTTTTATCAAATTTTGCGGATAATAATATTTTTGTATCGGGCTCACCACAGCTATATATCCCTGGACAGGTCAATGAAGAGTCTCCGCTTCAATCAAACAGCGAAACCAATAATGAAAGTACTAAAGAGAAAACAGTCGATTTACATTGTATTAATATCCAAAGTGAGGAATCTTTCGCAATGGAGAATATTCAGAATTCAACCACCGCTGAAAGCGCAGTAAATCCTACTAATGAAAATGAAAATTAGAACATTTTCTGTGATTGTCACTCACAAATATATAGTACACATACCCGATGATCTGTTTGTCATGTATCAAGTTAATTTGATCAAGTTAAGATATATTGGATCAACATCGGT |
| *Period* | | CCACATATCTTTGGGGAAGCCTAAAGTGGACGTGAGCGATGCTGTAGGCTTCCCCAAAGATATGTGGGTTGGCAGGTCGTTTATTGATTTCGTTCATCCAAGGGATCGTAATACTTTCGCATCGCAAATCACGAACGAATTGGCTATTCCGAAAATCGTATCCCTCACAGAAGAAACAGATCAGACAATGGAAAACCCGGGCTCGACGATGGTCTGTCGAATCAGACGGTACAGAGGGCTGAGTTGCGGTTTCAGTGTTAAGAACACGACAACCGCATACCTGCCTTTCTTATTAAAGTTCAAATTTAAAAACGTCAACGAAGATAAAGGAAATGTTATTTATTTGGTTATACAGGCAGTGCCATTCTTTTCTGCGTTCAAAACATCTAACGAAGTTCTTGCCAAAACGGTGTCGTTTGTAATACGTCATAGTGCTGACGGAAACTTAGAGTATATCGATGCTGAGTCAGTGCCGTATCTGGGATATCTACCTCAGGATATAACTAACAGGGACGCACTGTTGTTATATCATCCAGGTGACTTAGGTTACCTACAGGAGATCTACGGGTCACTCGTTAAGGAGGGAAACGTGACTCGTTCAAAGACATATAGAATGATGACACAAAACGGCCATTATATGAAGGTCGAGACGGAATGGTCAGCATTTATAAACCCATGGTCGAAGAAGCTCGAGTTCGTAACTGGGAAACACTACATTATTGAGGGGCCAGCAAATCCAGACGTTTTCCAAAACCCAGAGAATGTGTTAAAACTTACAGAAGAACAGAAAAATCAGGCAAAGATGTACAGGGACAGCATTATTAGAATTATGAAAGATGTATTGACAAAACCCGCTGAAATTGCTAAACAGCAAATGAGTAAACGCTGCCAAGATCTAGCTCACTTTATGGAGATGCTTATAGAGGAACAGCCTAAACCGGTAGATGACCTTCGACTTGAAATACAAGATGCTGATCACAGCTATTATGAGCGAGATTCGGTAATACTCGGCGGTATCTCACCTCACCACGAGTACGACAGCAAGTCCAGCACCGAAACACCCTTAAGCTATAATCAGCTTAATTACAACGATAATCTGCAAAGATATTTCAACAGTCACCAGTCGAACGCGTTTGTGGATAACAATTTATTACCATCAAGAAATCCATTATACTTATCTGCACCACATTTTTCTGAAAGTATTAAAAACGTTCCCTCGGCGATGGAATATTCTGGAGATGTGATTGATTTGACCGGCCCTGGTGAGACGAGCGGGGTCATTGTTTTCAACAAGAGCCCTACAATGGGATTAAAAACCTTTAAGCATATACGTCTCACTGAATCCTTGCTTACCAAACATAACGCTGAAATGGAGAAGGAATTAATGAAAATTCATCGCGAACATAGGTGTTATAGTAAGGGGGACAGGGTAAAAGTATCCAACGAAGCTAGGCAGAAGAAAAAGGAACATTTGGCCAGATGTAATGCGGGTTTCCAAACAATATCCGCCGCAAATAACACACCATCGGTATACGAAAAGCCCCATAATTTGAAACGATCATCGAAGCAGATGGAATCGGAGCCGATAGCTAACAAGCATCATTGTCCGTCGTCTCGGCAATTCCGTCGTAAACAAACTACATCGAGCGGTGGCTTCGCTCAGCCTCCATCGGCGACCAATCCCGTTTCCACTTCCTCCCAGTGGTCAAGCAGTCCGGTGAACAACGTCAACCCGTTTATCCTCGGCGTCAGGATGCAGCCACCGATGCCGATATTGAGCCCGCTTCCCGTTGTCTCAGGAATGTTCCCGATGTACTATACGCCAGTGACTGCTACCGTCACCACTAGTGAGGGCAGGCCATCAGAACCGAACTACCACCGAAATAACATGAATAACAATCAATTCCAACAACCTCTAGCCCCGTGTATGATGTACGGACAGCCGATATACGGATCGCCTATAATGTACTCGCCAATAAGCCCGCAAAGCCCTTGCTTTCAACAAACGGTCATTGCTCAAAATATGCACTATAATAATGCGCTAAATCCTCTTTGTCTGACGAACAGTAACTATGAGGAGGCGTGTAGGGCCAGTATCCCGGCTAGATCTGTTTTGAGCGCGCATCGTGGCAAGAAACGAATTGAGAGGCTTGAGAAACGCGTCAACCAAAACAACCAGGGGAGTGTTGACGCAGAATCCGGTACTTCACATAATGCGACATCTGCACCCGAAAGCATACCTTCAGGGGACCGCAATCCCCAGGGAAGATTGCGTAAAAGTGAAGAAACATTAGATAGAACAGACGGAGAGTCCAGCTATTCATCATTTTATTCATCCTTTTTCAAAACCGAATCCGGCAGCGCAGAGGATAGCATTGATATCAAGAAGACAGGCAATGTCAACGATAAGAAAAAAGTTAAAACTCCCAGGAGAAAAATGGACCCGCCATGGACTGAACAGGTGTGCGTCACGTCGGAGTTGGTGTACAAGTATCAGGTTCTGTCGAAGCCTTTGGAAGACGTCCTTTTAGCTGATAAACAAAAAATGAAATTAATTGAACAGCCTTCATTGGTCAACGATCAACTAGGACAGCTATATCTAGACCTTCAGTTAGAAGGAGTGGCCGCACGGCTCACACTAGAAGAAGGGATGACTAGTTCCAGCAGTTCGGGTGAAGAAGCATGTACCAGCTCTAAGATTGTGAGGAAAAAACGTGAATATAGCAAGTTAGTTATGATGTACGAAGAGAACGCCCCACTCCCGTCGCCACCTAACCACATAACACCCGATATTGAAAGTGGTATACCTTAAAATATCGTATCGGTTTCAATAAAATTACTTATATTTATTAAGGAAGAAATGGGGATTCATCAACGGTTTATCAGGGAAAAGAATGCTGACATGAACCGTGACAATAACGAAACATGGTAATAACGCTCAAAGAGAGCGATAAAATCAATTTACTAAGGAGTACATGAAAATAGCATAATACAATGAACAATATATCCTATATAATTTAACATTTGAAAATGCATCGAAGACAGAAAACATCTCATTATATAAACTGGATCTGGTTTGGATGGGAGAATATATCTTTCAGATAATTGTTCTTTCATCTACGTAAAAAATATACTTCAGCTTAAAACAACCTACTTGAAGAATTATAAGAGTATTAAAAATGTAGCGACAACTGAAATTACATGTAATGACAGCCGACTTTGTACTTCGGTATCTGTTGGGAGTAATTACATCATAATTATAAACGTCTAATATGTTGACTATTCAATAGTGTTATATACATTAGTACATAGTACTATGATGTATATTCCAAGCCTGAAGAGATAACGATTAAATCACGGCTAAGTCCCTAAATAAAACGTGTTTTAATATCTTTGTCTCCAGTTCGATATGCATTTGAAATGCTGTCAATTGATGTTGCTTTTAAATGCCCTTTCCATACATTTTTGTAAGCGTCTTCAAATTTCTACTTACTAGGGCTTTTGTATAACGGATATTGAGTCAATCAAACATACATCCATTCTATACGATTTCCTATAAACTATGTATGTGCAGTAAATTCTTTATCTCCTTGAAAATTATATTCAATTGGCGTCTAATATTTTGTGACATTTGTGTTACATTTTTTATTTCCTTTATGATTAGTCCCTCTTAGTATAATCTAATTTAAGCTCTATGATTGTGGTCTGGTTCATCAGTTTCATTAGATTTTAGTCAACTTTCTGATCTCTCCGCATATTGTTTATGATGTCGCTTGAGAACATATACAATATGTTTATCAGTTATTAGATACATTTAAGATGTAATTCGTCACCTATAGGGATAAGTTCCAGCAGCTGAGCTACATAGTCAATTCTCCTTAGATATAGTATATTTACCAATTTATGCCAAATGAATTCCTTTTCAGTTAATTAATTCCGTGGATTAATATACAGTAATCAGTTCACGGTATATTGCATTTATGTTTCTATACGTAAATAATTTCTTTAATTGCATTCAAAAAGTACTTCGTCTTATAATTTATAGTTATTCCTTTGTAAACATT |
| *Timeless* | | CACACAGCACGTGCAGTGTACACGCGACCTGCACCAGCGTGACCTACAGTTTGCGTTCAGATCGATGTTTCTGAGATCACGTTGTATGTATTATAGACCTTTTCCTACGTCATTCGCAAAATGTTCTACTTGGTACAGTGGTATTGCGTACAGTGACTAAAAATTTGAAGTCAGTTATTTCAGTGGAGTGCTTACGCATGTTATCATTCTAAAGCGTGTCCTTTATATCTACGTTAAGTTTACTTTATATGATATAAACGTCAACTTTCAGTGTTAGATTACACGTTCCATGCAAGAAACGTAGTACAATTTTATTCTCATTATGGGTCAACGTTAATTGTTTCTCAAATCTATAAAAGATGGAATGGGTTCTGCGAAGTCCGCAGATACACAGTACATTCAGCAACCTTGGATTTTTACATGCTGATGGATATCACGTAAACGAAAATTGCAATTCTGCCTTGGAGACTATTTTACATAATATTTTAACAGAAGATAAATATCTACGCACATTCCGGCGAAATATAAGTTTCGGTCAGAACATCAAGAAGGATTTTATACCTTTGTTGATTCATGCAAAGGAAGACAAAACAATAGAACTTTTGATAAAAATATTAGTGAATTTAACTATTCCTATTGAATGTTTACTTTGCGTCGAGGTTATTTCGAAAAATGATTTTGGTCGTCATACGATATTTGAAATAAATAATTTACTCAGCACTACCAAAGCCGCATTTACAGATCATCGAGCTACGAAAGTTGTTATAGATTTTTTAAAGAAAAACGTAAACTGCGAACAAAAATCAGAGTTATCTGCAGAACAATGTACAAATATAAGCAATAGTCTTCTACTGTTGAGGAATATTTTACATATTCCAGATGACCATCAAAACTCTAATTATAACGGTTCACCTCATGTTGTACAAAATCAAATATTATGGAATATTTTTAGTCAAAGTATTGATAAAATACTAATTAAGTTGATGACTATACCTGATGCGACAAAATGGGCTGTAACAATGGTTCAGCTCATAGCTCTTTTATATAAAGACCAACATGTGGTAACGCTTCATAAACTTCTTAACATCTGGTTGGAGGGATCTTTATCAGAGAGCTCTGAGGATAACGAGAGCAACACATCTCCTCCTGACAGAGGTAGTGATGATTCATCACCCATATTAACATCGGATCCTACCTCGGACTCGTCAGACAACGGTGGAAGTGGTAAAAGCAATGACGAACCTAATTCCGTCAATAATGGGTGGGATTCCTCTTCAATGAATAATGCTAGTGAAAATGGTAATCAAGTATTTCATACTGCCAATGAGAAAGGTGTACATTCTGTAAAAAAAAACCTTATAAAAGTTGGTACTACAGAGGAGTCCATGAAAATTGATGAAGAAAAAGTAGACTCTTGCAGGGAAGTTACGAATTCGGAATCAACGAGCCATGACCACAGTCATGGCTATACAAGCACGAAGAAACCTAATATAACTGAAAATTCTGATTGTGGTTACGGTACACAAATTGAAAATCAAGAATCAATTTCCACTTCCAGTAATGAGGACGATGTACCCACGAAAAAACCTGTACATCTTAAACCGCATAATCCTAAACAAAGAGTGAATAATAAAGCTCGTTCTGGAATAACACCTCAAGAACGTAAACGAAAAAAAATAGTCAAAAGAGGAAAGTGTAATATCATTAACGTGCAGGGGCTTACTCACAAAACACCCACTGACGATGATATATCAAACGTACTAAAAGAGTTCACTGTTGATTTCCTGCTAAAGGGTTACAACTCCTTAGTGCAAACACTGCACACTCAAATCCTCACTAATCTGCAACTTGAAATTGACACGTCTCATTTTTTCTGGCTCGTCACATATTTTCTTAAATTTGCTACACAAATCGAATTGGACTTGGAACTTGTGTGCTCCGTATTATCTTTTGACATTATTTCCTATTTAACCGCTGAAGGCGTTAATTTATGTGAACAATTTGAGCTTGCCATTAAAATCGATGGAAATGATTTGAAACCCTGTATGAGAAGACTGCATTTGGTTGTAACAGCGATACGTGAATTAATTCAAGCTATTGAAATATACAAGAAGTTTCCACACTTATGCAAAGAAGATCAGGAGACGCTTATTAAATTACAGATAAAAATATGTGAGACTGAAGATCTCCGATCGTTAATGGTTCTGCTTCTGCGTCATTACAATCCTAGATACCATAGCAAACAATACTTACAGGATCTGATTGTAACGAATCACAATTTATTGACGTTCTTAGACAAAGTTATGAAACTTCCCGACTATAGAGGCTCCACCAATATGGTTGATCATCTCAGGCAGTTTACGTCTCCAGAAATTATGTATCAGTATGGTCTATTATTGGAAAGTTTTGCTGATAACGGCGAATACGTCAACGATTGCATTTTCACCTTAATGTATCATGTTGGAGGAGAACTCGACAGTTTGATCAGCCTCTTCCAACCGAAAATATTGAAAATTTTCACGTTTATATGGAAATCGGAATTTGAAATTTGCGATGATTGGTCTGATCTTGTCGAGTACGTAATAAATACGTTCATAAAGAAGCCTCACACGTTACAAAGTGTAGTCGAGTTACAGCAAGATAATGAAAGCTTTGATGATGATAAGCTAATATTGAAACAAATTAGTGCAACGGAGCTACAATCGAACCAATTGAAAGAAAGCTGGACAGAGGACGAACTGTCATCTCTAAGTTGGAATTTTATGCAGTGTGGCACATCGTTAAACGTTATTGAAGATATAATAAAACTTTTTAAAGAAGACGACATAATTAAATCCCGGGAATGCGTTATCAAAGAACTTTTAAATCAGAATCTAATAAATAAGAATGAGTACGAAAAATTCATGAAGGGAGACAATGATGGAAACAAAGCAGTACCAATAAGTAAAGAAACGAGAGATGATGAAGTTAGAAAACTCTGTGAAGAGATCGCAAAGGACGGTAAATCGAAGTTTCTAGATTGGGTACAAAAGGCACTATTGGACACATGTTTTGCCAAAATATGCATGGACAAAAAAATCACAAGACGAGTTACCACCGACTAATGACTGTAAATTATTAGATTTTGAACTGTTCAAAAAGAAGTCTGTTGAGTTTCCACCTATGTCACCAGTGGCATATCATTCGTTGCTTCTAAACCAGTCTGTGCAATTGGTACCATGGAATTGCGATCAAGGGTCATTTTGTAAAGATTTAAGATTTTTACAACTTTTACACAAGCTCGGTTTCCACATGCCTATTGACTCTGCCAAGATATTTATTCGTATACCTAATTTCTGGACTGCAGAACATTTATATGAAATAGCAGGAAAAATTGCACCAATCGATAATTCAACTTTGAAGTTCTCCGTAAGTGATATATCAGACTATAATTGGACAAAGGCAAAAGTGCATGATCATTTTATGATACCTACTGACTTTGGTAGAGATATTGTAATGAACGATACATCTGATAACTCGTACCAAATACATAAACATTTCTCCGCGATGGTCAACTTCACACCTATGCCTGGTACTTCCTTTAATTCAGATAACAATGAAGTCAATGAACAATCGAACAGCTGGCTAGAAGTGGTGATGAAATCACAAGAATATAAAATTACTTTGAGCCGTTTAGTGGAGGATGATAACGATGATGATTTGATGAAGTTACCAGTGATAGAGAAAGATTCTATCACCGCGATGATAACCGATATGGAGCCGGTTGCGGGGCCGAGTAAAAACAGTTCCGCTCCTCTCTTGATGCCAACAGTAATGACCACTACACAGGACGTGTTACCTTTCAACAATAATCTTGCCGAATCAGAATTTTACAATAACAGTATATGCGAAACAGCATCTGTTGCATCTGACCTAACTCGAATGTATGTCTCTGATGAAGACGAAAAGCCCGACGTGATCCTGCGACCTGAGCTGGAGATCAATATGGACAAGCGATGCTCAAGTCCTTCAGAGGACACGAATGTTAAACGTTTTCGTATAGATTTTTCTCCTGATTTTTAATATTAAACGCATCTTCTACATAAGTTTAGTATTACATGTACTATGTTTAGATTATGAGTCCATTATGTAATTGAAATATATGTATCTGTTATAGTGGTGTTATGGAATGAAATGAGTTATTAATTAATTACCTTGTAGTAATGTACTTATAACAAGAAAAAAACAGACAAGTAGTTATAACTAGAAAATAAATTCTATTTAAAATTTCTACTTTGTTTAAAAAAAATCTAAAAAAAAAATATTTTTCAGAGAGTTACATTTAATTATTGTTATGATAAAAAGTGTTATTTTGTTTTCGGTTCCTCAAAGTTTTTACTAAATATCATGTGTAGTAATTAGTGCAATATGTACGTGATCTTCGTGTTTGTTTTCTGATAATGATTTATAAGTTAGAACAAAAAAGTAAACGAA |
| *Clock* | | GGGGGGTCCTGTAGTGACAAAGGTAGTACGCCGGGTACAGCGACCGTGCCGACGTACGGTGATGGTTCCAAATACTGTGCACCGATACCGTGCTGTGGTACTAGCACTGGCTCAGAGACTTTGCGGGTATCATGCGAATTGTTCCTGGCGGAGGACCTGGACTCGCCTGAGACCGAGGCCGTGTCCGAGACGGAGGGCGCGTAGGGAGGCTGCGACGCCCTGGACCAGGACACGGTTGTAGTACTAA |
| *Cycle* | | AGCGATCACCCAGGCTCCTCCGGCTATATTCTTCATAGGGCATTAATAGTTGAAGTTATCCGTTTCATACGGATGTAGCGAATGTCATGGCAGCGGCCACGGCAGACCAGAGAAGTTGACCTGCCCCCCGAGACCGGCGTCCGCCTCTAGGAGGCTCATGATGACCGCCATAGCCGCCTCACCGTTACCATCAATACCTAAAGAAGGCAACGGAGGCGAAACCGGCAGGGGGCTCCCCACTGGTAACTCGATCCCGTTCTCGCTCTGCGGGGGGGAGTCCCTAACGCCGGCGACGCTCAGCGGTACGTTGTTACGCACGTGCAAGTACTCGGGCACTTCGAGGCCCAATATGTTGTCCACGAGGGACGTCTGTTGATTGAAGACTGCGTCCTGAAGCAAATCTGGTGGCAATTCGGGTATATATTCGGTTGGAGATCGGCGCAACACCTCCTCGGCGATGGTGCTGCCGATCTGATGCGACTCGACCCGAGAGTCAATGAGACGCTGCATCTCAGTATCCGGTTTCTCTTTGTATATCTCGAAGGTTGACTGCTCAGCGCTCTGATGAATAGGCATGTGGTTGTCTGATAGTACCGTGTTATTAGCCACTAGACATTCAACATCTTTAGTCCAGGGGTTCCTGAAGGG |
| *Shaggy* | | GAGCGAGTAGAAGTAAAGTCTGAAGAAAATAAAATCTGTCGTACACTTTAGTGATGTAAATTTTGAAAATTTATCAAAATGATATTGTAAAATCCTAACAAGATAGTCTTATTTTAATTTTAAACTGGTATTATTTTAAACTTTCATAGTTATCTCGTAATCCGTCAGCGAACAGCGTAGTGAGTTTAGTGTGAGTGCAAGGGTGGTAACGAGAAATGTGAAACATCTATGAAGTTCAGGATTCTAAGGCCGTTTATTTTATATCAATAATAATGAGCGGACGCCCTAGGACCACGTCTTTCGCCGAGGGCAGCAAATCCGTTCGAAAGACATTCGATAACCAGCCCCCGAAACCACCTCTAGGAGGCGTAAAAATTAGCAGTAAGGACGGCTCAAAGGTGACGACCGTAGTAGCGACGCCGGGGCAGGGGCCCGACCGGCCCCAGGAAGTTAGCTATGCCGACATGAAACTCATCGGTAACGGCAGCTTCGGCGTCGTGTACCAAGCCAAACTGTGCGATACCGGCGAGCTCATCGCCATCAAGAAAGTGTTGCAGGATAAAAGGTTTAAGAACCGTGAACTTCAAATTATGCGACGACTTGAACATTGTAACATTGTCAAATTGAAATACTTCTTTTACTCCAGCGGAGAAAAGAAAGATGAAGTGTACCTGAATCTGGTGCTGGAGTACATACCTGAGACTGTGTATAAAGTGGCACGTCACTACTCCAAAGACGAACAGACCATACCTATTAGTTTTATAAAGCTATACATGTACCAGTTGTTCAGAAGCCTCGCGTATATCCACTCACTTGGTATATGCCACCGAGACATAAAACCGCAGAATTTACTTCTCGATCCGAAGACCGGTGTTCTAAAGCTTTGCGATTTCGGATCGGCGAAACATCTCGTATGCGGAGAGCCTAACGTTTCATACATATGCTCGCGATACTACCGCGCTCCGGAACTTATCTTTGGCGCCATTAATTATACCACGAAAATCGATGTGTGGAGCGCGGGTTGCGTCGTCGCTGAACTGCTTTTGGGTCAACCGATATTCCCCGGTGATTCCGGGGTCGACCAGTTGGTTGAAATAATCAAGGTTCTGGGCACACCGACACGTGAACAAATACGAGAAATGAATCCCAACTACACGGAGTTCAAGTTTCCACAGATCAAGAGTCATCCGTGGGCAAAGGTGTTCCGTGCTTGTACGCCGCCCGACGCGATCTCGCTCGTCTCGCGGCTGTTGGAGTACACGCCGGGCGCGCGTCTCTCGCCGCTGCAGGCGTGCGCGCACAGCTTCTTCGACGAGTTGCGCGAGCCGACGGCGCGGCTCCCGAACGGGCGGTCGCTCCCGCCCCTCTTCAACTTCACGGACTACGAGCTCAGCATCCAGCCGTCGCTGAACGAGTTCCTGAAGCAGCGCACCGCCGGCGAGGCGGCCGGCGGCTCCGCGGCGACGGCGCCCGACCACGCGCAGGAGGCGTCAGGTACGCCGCCCGCCGAGACGGTCGCGCGCTAGCGGCACTCACCCCCTCTCAGCTGTAACCGTTCCGTTGTGTGGCTGGCGGAGACGCTACACACCTTGCTAGACGCCGTCGCTCCC |
| *Double-time* | | GATAATTTGGTTTTACAAAGTTCACTGTAAAAAGGGTGGATATTTTTATTTCATATACGATTCAGTTTATAAATAAAGAATTGTTGGTGGTGAAGAAATCAATTATAAACCAAGACCATTTTCACCCAGATATCATACTTTATTTTACTGATACAGTTGTGCGTCTATCGCAATATGGCCGTCTTGTAATGATTTGGTGGTCAGTGATATTATTGTGATTTCGACTCCTAACGAACAATAAAATGGAACTACGTGTCGGTAACAAGTACCGACTGGGCCGTAAAATTGGTTCAGGATCTTTTGGGGATATTTATCTGGGAACAAATATCGTCACCAGAGAAGAGGTAGCTATCAAATTAGAATGTATTAAGACAAGGCATCCACAGTTACATATAGAAAGTAAATTTTACAAACTTATGCAAGGAAGAGTTGGTATCCCAGCTATAAAATGGTGTGGCTCAGAAGGCGATTACAATGTTATGGTAATGGAATTACTCGGTCCCTCGTTAGAAGACCTGTTCAACTTCTGTTCTCGTCGTTTCTCTCTGAAGACTGTACTACTTCTTGCAGACCAACTAATAACACGTATCGAGGACATTCACTACAGAAATTTTATCCATAGAGATATCAAACCAGATAACTTTCTAATGGGTCTAGGTAAAAAAGGAAATTTAGTGTACATAATAGATTTTGGCTTGGCAAAGAAGTACAAAGATCCACGCACAATGCAACATATCCCATATAGAGAAAACAAAAATTTGACAGGTACTGCTAGATATGCATCTATAAACACCCACTTAGGGATTGAACAGTCTCGCAGAGATGACTTGGAATCTCTGGGCTATGTGCTCATGTACTTCAATCGTGGTAGTCTACCTTGGCAAGGACTTAAGGCCGCTACTAAACGTCAAAAATATGAGAGAATATCAGAGAAAAAACTATCCACACCATTTGATGAACTTTGTAAAAACCATCCAATCGAATTTCAACTCTACCTTAAATATTGTCGTCGACTACGCTTCGAAGAGAGACCCGACTACAGTCACCTGCGTCAACTGTTTCGTACGCTTTTCCACAGGGAAGGCTTTACCTATGACTACGTTTTCGATTGGAACATGCTAAAGTTTGGAGGACAACGTGGTAACTATCAATCAGGGGCCAATGACCGTACACTCAGACGTCATGAACCAAATGCAGAACAGGAGAACACGGCGGGCGCGGCCGGACAGCCGAGTACCACGGGACCC |
| *Vrille* | | AAAGCTTCATTGATTGTAGACGTGTCCAACTGAGTGGAAGTAACACAACGCCATTGGATATTTCGTCATAACATTGTGATAGTGACAAATTTTAATTCATTATACGAACGTGAAAAGTGGGTGTGAAAGATTTTTTAAATATATTCTTCTAAGACGATTATTGTGAACTTTGACCGTACTGCGGTGGTGAATTTTTGATATATTTTTGTGGATTGTGCCATATATTACCCACTATTTGGATAATTCCACACGAGGAGTACATAAATACAGCAAATATTTTTAATCACATTGAAGATACGTGCGGACCTATTGCCGTCCGCCGAAACATGGTTGCAGAGTTTATCTTAAGTCAGCAGCAGCTGCTGAGTGCAGGTGGTGGAGCCGCACTCGGCCCTGCACCAGTTCAAACACGCGCTCCCCCACCTCCCCGCGCGCGCCTCTCCGTATCTCCTCATTTCCCAATGGACCAATCTCACCCGAACAACGGCAGTCCCGAGAACCCGGAAGACTACTCGTCTTTCGACTTCAAACGCAAAGAATTCTTTGGACAACGCAAACAGAGGGAGTTTATACCTGATAACAAGAAAGACGATGGATACTGGGACCGCAGGAGGAGAAACAATGAAGCCGCTAAGCGTTCCCGTGAAAAGAGACGCTTCAACGATATGGTTCTCGAACAACGTGTCGTTGAGTTGTCCAAAGAGAACCACGTAATGAAAGCTCAGTTAGATGCTATAAAGGAAAAATACGGGATATGTGGAGAAAATTTGATAAGCATAGACCAAGTGTTGGCTACACTACCCACTTGTGATCAGGTCCTTTGTGTAACTAAACGGAGTAAGCTATCGAGTAACGCTCTGTTCTCGCCAGCTCCACCACCGCCCCCGCCGGGCCCCCCTGCCTCGCCGCCGTCGCCTCCTCCACAACGCGCTCCGGAACCTTATCAAGAAAGACTTCCAGTCCCAGAGCCATACTATCCGCATCCCGCACACTACGAACCTCCCGGTTCAGTACTCAACCTGTCCAGAACTCGCCGCGCACCTTCGCCATATGAGCTGTCCTCTCTCTCCGGTTCAGGGGACGAAACCGCAGAACCTTACGCACCAGAGAATAACTGTCTGCCGCTCAAATTACGACATAAATCTCACCTCGGTGACAAAGATGTCGCCAGCGCTCTCCTCTCCCTGCAGCACATAAAACAGGAGCCCGGACCCAGATCGTCTCCTTCCTGGGACGGTGAGGGATCGAGCGACGAGAGAGATTCTGGTATTTCCCTGGGTGTAGAGTACAGGCCACAGCCAGAAAGGATTCCAGAGGAAGAGGACGCTCATTTAAAGGCGGAGCTCGCCCGACTCGCGACTGAGGTTGCGACGCTGAAGAACATGATGAATCAGAACAAGGCGCGCGGTCACGAGCACTGAGCGCGCCGCCATCGCGTTCGGAGGTCGCCCCGCGCGTTGCGCCTGGACCGCGCAGCCCCGCAGCCCCGCAGCAGCCCCGCCGCAGCGCAGCCGCAACGTTCGATGACGTGTCGATTCCTGTACAAACTGTCTCTACATCTCTCCTAGTAGACGAAGTTACCACGTTGTAAAGAAATGATTTTATATATTTGTATGATATTGTGCAGCTGTAGCGGCTAAGCCGTTTCGCCTAATTGTAATCCTTCCTAGTAGTTTATACGTCTGTGTGTAGCAGCAGTAGGTTACGTTGTACGGTGTGCGCTGCAGTCTGCACTGCATGTTTCCACGTCGATGCACCTGCAGCGCGTACATTGGTCACCGACGCAACCTACGTCGATGAGACTTATGTTCGGTTAACAGACTTTTCGAGCAGATAAAACCTGTCTACTCAGTGATAGAATATAAAAAAATGCATTTTTAGTGTTGAACTATATATGTATATGTAATATAAGTATAGAAGTAGATCTGTTTCATCTATTCGAAGACTCGATAGTGAATCGATGGCTAAAGACTATGATGTTCTTGTAAGTTAGTTCTAAGATAAATTAAGTTCTTAGATGGTTATTTATAAAAAAAATTGTATATTTCTCATCGGAATGCCCGTTAAGTTCCAAACCAAAAATGCGTTAGTTCTTCTGTTAGTCGATGAAATCGCATATAATTATAAGGTAATCATAAAATCAGAACGTATTGGATAATCAGGATAGGAGTACCAGTGCCTTTGACAATACATACCTACCTTAAATAGCTACCTACCTAAAGGGATATTTTTGTACACCTGTAATAAAAAGATAATTGTGTCGATTTCATTCGTGGATATTGTTAGTCGACGTTTATTATGTGTTTCATGTGGATCGAGTTTAGAGTGAGTTTAGATCGAGTTCTGGAACACATAGTAGCGTATACAAATAAAGTAAATTTATATCCAATTCGAAAAAGGCTCGTCGGCAAGTATTCCCGTTGTTTGTAAGTTCTCTAGTATTGCCCTAAGGGCATGTGATAGGACTGAATGGTGTTATAACTAAGTATTTATTTATTATTCGGACCGTGAAAGAGGATTTTACAATTTATTATCTTTGTATAAGATCACTATACGATATGAATAAATTGTGTACATTAA |
| *PAR-domain protein 1 ε* | | TGCCGTGCGCCGGTCCTCGTCAATCCTGCAGTGAGCCGCACCGGGAACGGTCGCGTCTTCTGTGTCCGGTCTACTGTTGCAACGATTGAGTATCTAAGAGTAAACGAGAGCGGCGCGCGCTCGCGGTTTTGGCCGGCGCGCCGCTTGGATAGGCATGGCTATATAGACATGGGTACCGACTCCGCTGTTCTTGAGCCTGACAGCCACATCTTAGAGGACGATCTACACGAAAACTGCTTCTTCCTGCCGCCACCACTGCCGCCGCGACCTTTGCCTGTGCCGATACCATTGCACTCGGGTCGCCACCAACACTATCGCTCGTTCTCAGGAAAGTCGGTGAACAGTAAGGACGCGTTAGAAGACAAGAAGGATGACAGTGACCTGTGGGAGGCGCAGGCCGCCTTCCTCGGTCCCAACCTCTGGGACAAAACGCTGCCCTATGACCCCGATCTCAAGTACGTGGACCTAGACGAGTTTCTGTCGGAGAATGGCATGCCGGGCGAAGGTCTGGGCGGCGCGCACCTGGGTGGCTCCGCCTTCGGCCCAGCGTTGGGCTTGCAGGCGCCTGTCACCAAGCGCGAGCGTTCCCCTTCACCCTCCGACTGCATGAGTCCGGACACCATCAACCCGCCGCTCTCGCCGGCTGATTCCACGTTTTCTATGGCGTCATCAGGACGCGACTTTGATCCTCGAACGCGCGCTTTCTCCGACGAGGAGCTGAAGCCGCAGCCAATGATCAAGAAGTCCCGGAAACAGGTAAATGAATTCGTGCCCGATGACCTCAAAGACGACAAGTACTGGGCGCGCCGGCGGAAGAACAATATGGCAGCTAAGCGTTCCCGCGATGCGCGCCGCATGAAGGAAAACCAGATCGCGCTGCGAGCTGGATACCTTGAGAAAGAGAATATGGGTCTAAGGCAAGAAGTAGAGCTGCTTAAAAAAGAAAATCACATCCTACGCGAGAAGTTGTCCAAATATGCGGATGTATAGGCGGTGCGGGCGCGGATCCGTCGCCCTTGCGTCCCGCCTTCGCCCCCGCCCCGCAGCTCAGCGCTCCCCGCCCGCACCGCCCCGCACCGCACAAACACGGCTCTCGATATGTCGCGGGCGCAGCGTCACCGGGTTCGGCCCCTTCGCCTTACTTTATTCAGCTCTACGACTTTAGTGTAAGCGTCCCAGCCCCACCCGCCCCGCTCCCCCTCCCCAGCGCCCTACCGACGTACGCTGCAACGTCTGGCAGCAGGAGCGCGTAGTCCTGCGTCCCATTAATATAGGTTTATTAATAAATTAATGTCGCAGTTACCGTAAGTTTATATTAGTTTAATAGGTGAATCTTTACCAAAGTTCCGTTCGGGCTCTAATCCATCGTCGCGTCGTATATGAATCTTTCCCTCGGACTGAATTCGACGCCGACAGGCTTAGTCTAAGACGTTCGGACCAAAATTGTATGGGATTTTATTTATGAAAGGTGTATTAGCGTCGTGCCCGGGACGTGTGCGCGGCCCGCGCCCCCCCGCTCGGCCGCTCGGGGCAGGGCGTGTGCGGCGGGGTGCGCGCGCGGTGGGGCTGCGGGTTGCGCGCACGCGTCTCCCGGTTTGCTCAAAGGGGTCGCGCAAGCGGCTGCAGTGTCTGCGTCTGTATATAGGTAGTTGTAAAAGGGCCCGGCGGGCAGTAGTCCGCCCGGCGACGCCGCTCTCCGTCGCGATCAGACGGCGGGGCTCGCCCGCGTCCCCGGCCCCCCGATCCGACCCTGCCGTTTCCGTAGCGAGCGAATACTAGTTTCGGCGTTCACTTAGTGAATAGTTTAATTAGTGTAGTGCGTAGATTCCGTAGAGTAGTAGGTATTAGTATATTTTTGTTATGGCTGTAGTTGTTAACGTTTTTGAAAGTTTATATAATTTTTTTTAGTTATAAAGAAACTCGCGTTACTGTGTCGATGTGGGTAGGACCCACTGTTAGCGTATAGCGCATAGATAGGTACCTCACTCTTAGCTCTTCCCCACCCTGCCCCCGCGCGTCCCCAGTCCGCGCTCCTTGTCCTCTGTCCTGGGCCTCGTTCGCGGTGTCGCCGCGCGCGCCCTGCTTCGGAATGAGAACGTTCCTCCGCCCTCCGAACTTGATTCGTGCTATTGTTATAACAATATTTTTGTAGGTTTTTTTGTCGTTGATATTGAGTCAATTGAATGTTTACTGAATTGAATAAAAACTAATGTTTACGAATTAAGAATAAGAAATTGTTGCATTTGAAAGTATGTATTCTATTGTTAATAAAAGAGAATAATAAAATGTTTTTTGTATAAACGTTTTACAAGTGAATTAGTACGATCACGAGTGACGGAAGACGCTACGCCGTGCTCCCTCACGCCCTTCAAGCGCCCTACGGTTTCACTTTGAAATGCATTATTTCATATCAGCAATATTCATATTTTATTGTATAAATATATTATAATTGTTATATTCCGCCGCGTGTATATATTATGCACATTGATGCATAGCTACGTATAATATAGTATTGTAAAGTATAGTATAGTATACATATATACATGGCTCGTGTACGATATTGTGACTGTTTCGCCGGATATTAATGTAACATGGGTATAATAAATGTTAAGTATTGTGTTAAAAATTGACACCGTTGTAGAATGTACATTGCACGCGGTAACGCAGTCTCCCTGTCACACCGCCTCGGCCCCGCAGAACCTTTCCCCTTCTCCCCGCCCCTCCTCCTCCTCCTCCTCCCCGCACCGCCCGGCACTAGTCCATGTGCTTCCTTATTTATTATATAAGCAGCTCCCGCGGGAATGAGGCTCCGCGCCCTCCTGGAGCGCGACACCTCAGCGTCGCAACAATGTCAAACAATAGTGATTTACATGTATGAGTAGTAATTTGTTTGAATTTAATTTATATGGTTCTATGGATTGAAAATAAACGTTAATTGTTGTA |
| *Slimb* | | GATAGCCGTATGGGATATGACCTCGACTACGGAAATAATGCTGAGACGCGTGCTCGTCGGGCACCGCGCCGCAGTCAACGTTGTGGACTTCGACGAGAAGTACATTGTCAGTGCCAGCGGGGACCGGACCATCAAGGTATGGAACACGTCGTCGTGCGAGTTCGTGCGCACGTTGAACGGGCACAAGCGCGGGATCGCGTGCCTCCAGTACCGCGACCGGCTCGTCGTGTCCGGCTCCTCCGACAACACCATCCGGCTGTGGGACATCGAGTGCGGCTCCTGCATCCGCGTGCTCGAGGGCCACGAGGAGCTCGTACGCTGCATACGGTAATATACATTTTCCCTCACTTCCCCTCATCGTCCACATATCCCCTCAGC |
| *Casein kinase II alpha* | | GGTGGGATAGGTGGGCGACGGGCGGGCGTCCATCTTGAAATTTTACTGGGTTTGAGCCGGTTTAGTTTTAGTAAAAGTGAATTTTGTAGTTTATAGTTTAATAATTTGTTAGTTTTGAAAAAGATCCATGTATGCTAAAACTGTTATATAGTTTAAAATAAGATTAAACACAGTAATGTTTTAGGTATGGATGTTGCTGAACATTTGATCATTTGACAATATCGTTACTGAAGACATTCGCGATTTCCGGAAAGACTATCGCAGATAAAACCTTAGGTTGGCAGAGACACAGTACGATGGCAGTACCTAGTAGAGCGCGGGTATATGCCGATGTCAACTCACAACGTCCGAGAGAGTACTGGGATTACGAAAGCTATGTAGTAGATTGGGGTAATCAAGAAGACTATCAATTGGTCCGCAAATTGGGCCGTGGAAAATACAGTGAAGTGTTCGAGGCAATAAATATCACAAATAATGAAAAGTGTGTAGTTAAAATATTGAAGCCCGTTAAAAAGAAGAAGATAAAAAGGGAAATAAAAATATTAGAAAACTTAAGAGGAGGCACAAATATAATAACTTTACAAGCTGTAGTAAAAGATCCTGTTTCTCGTACGCCAGCGCTCATTTTTGAACATGTGAATAATACTGATTTTAAACAACTATATCAGACTCTGTCAGATTATGATATAAGGTATTACTTATATGAACTTTTGAAGGCATTAGATTACTGCCACAGTATGGGTATTATGCATAGAGACGTGAAGCCTCATAATGTCATGATTGATCATGACCATAGAAAGTTACGTCTTATTGACTGGGGGCTAGCAGAGTTCTACCACCCAGGACAAGATTACAATGTTCGTGTCGCTTCTAGATATTTTAAGGGTCCCGAACTCTTGGTTGATTATCAGATGTACGACTATTCTTTAGATATGTGGTCATTAGGATGTATGTTGGCATCAATGATTTTCCGTAAGGAGCCTTTCTTTCATGGTCATGATAATTATGATCAGCTTGTACGTATAGCAAAAGTGCTGGGCACTGAAGAGCTATTTGAATATTTGGACAAATATCATATAGAATTGGATCCTCGATTTAATGACATTCTTGGCAGACATTCTCGTAAACGGTGGGAGAGATTTATACATTCGGAGAATCAACATTTGGTCTCTCCCGAAGCTTTGGACTTCCTTGACCGCCTTCTGCGTTATGATCACTATGAGCGCTACACTGCCCGTGAAGCAATGGAGCATCCTTACTTCTATCCAATCGTTAAGGAGCAAGGTAGAATGGCGTCGTCTAATTCTCCAACTCCAAATGCATTGCAAGGACCAGTAAATGCAGCAGAATAATCTTTAAGAGTTGAAACGCTCTTTATAATGCTATGTATTGATATAAACTTGTACAAGATTCCAGAAGAGACAATGTAAAAATAACATCCGAACAAGGCCACTGTTGGTGTAAATATACAGTGAATGAAATACTTGGTCCATTCAACCGACAATCATTGGTGTTATTCCATTGATAATATAATTTGGAAAGACAATATGATTTAGTGTTAATGATTAGTAAATCTGGTTTATTCGTTGTTTTAAAGTGTTATATCTCAGATAGGAACTGCTATACAAATCCGAGGAAGTGTTCATGTACCCGAGACGTTGAGAACCTTGTGGTGGGTGGGATTTAAATTGTCGCAAGTAAATACTGTTGAACATTGATTTTGTAACATGAAATTCGATAGATATTCATTTGAGGTAAAGATAAAATGTCAAGTTTAATTGCACTTGATCTTTTGCATTTATTAACCTATGGTATTGATATTGTAATTACTTGTATAATATGATATTGGTGACACCTGCATACCATTTGCAAATACTGGTTAGCAGCTTTCTGTTGGTACTCTACCTGTTTAAAATTTTGTATTTATGTAATAGAAGTAAAATGTAGATATGTGTGATATAAATACAAAATTTTAATATGATTAATTAATCGTTTTCTTTCATTATAAACGTACATTACAAAGGAGACCAGTACTTCTAATTATTATTAGATAATGTCGGATTTAAATATTGTTTCTATTAGTATATTTTTAATAAGTTATGTTAGCTGTCCTTATACATAATATAATTAATTAGCTAAAATCGAGTCGTATCTTCAATTTAAAAGTGAGCCTCATTGAGTATAATTTATTTTTAGTGTGATAACTCCAAGTTGTTGCATAAAGTTTTCTTAATAAACAATACTAACTTTGCCTGATGATGTTTTCGATTATTTATATTTTAAACTGCAAGAACCTATAATTTTATTTATGATATAATTTTTTTTAAACTTATGAGAATTGCAGTAAAGTAAAATTGCCCTTATAAATATTCGTAAATTGTATAAAATTATTTTATTCCATTTGACCTATTGCATGTTTTTACATTGCTACCATATTCATTTGAAAAGTAACCGTTTTGGTAGTGAAAATAGTAAAAATGACAGCCATTGCTTTTATTTACGGCAATAAAATTATGCGCATGGCTTATTTTGATATGTTTCCATAACTCTTTGTTTGACGTGCAAAAAAATAAAAACTGATTATGGTTAAAACTGCCGTATACGAAGGAACATTGAAGGTTTTTTTTTATTGGCAACACTGCTAAATTTTTTTTTTATTCGGGTTTATAATTTTTGTACAATGATGTCATTATAAAACAATAAATTTCTATGTGACTTGTATCCCCTT |
| *Casein kinase II beta* | | AAAGAATTGAAATTTCTCTCTCCCTCTTCGGAAGTAGATACCCTCCCGTCAAGCGTGGCTAATCAAGGCACGAAAAGGAAAATGAGCAGTTCGGAGGAGGTATCATGGATTTCGTGGTTCTGCGGGCTGCGCGGAAATGAATTTTTTTGCGAGGTGGACGAAGACTATATAAATGATAAATTTAACTTGACTGGTCTCAACGAGCAGGTGCCACATTACAGACAAGCTTTAGACATGATTCTCGACTTGGAGCCAGATGATGATCTCGATGACAATCCAAATCAGTCTGACCTGGTTGAGCAAGCCTCAGAAATCTTGTATGGACTCATCCATGCTCGTTACATTTTAACCAATCGAGGAATTGGGCAAATGTTAGAAAAATTTCAAGCTGGTGATTTTGGTCACTGTCCACGAGTTTACTGTGAATGTCAGCCAATGCTACCACTTGGTCTGTCCGACGTGCCTGGTGAAGCAATGGTGAAGTTGTACTGTCCGAGGTGCATGGACGTGTACACACCAAAATCGTCTCGGCACCATCACACCGATGGCGCATACTTCGGCACCGGCTTCCCTCACATGGTGTTCATGGTGCACCCAGAGTACAGACCTAAGCGTCCCGCCTCGCAATTTGTTCCTAGATTATATGGGTTCAAGATACATCCGCTCGCTTACCAGATCCAGCAGCAGGCTGCGGCCAACTTCAAAGCGCCGCTTCGGAGCCTCTCGTACAACAACGAAACGAAGACTGCAAGTAATATTCCAAAATGATTGTATAGCGGACGTGCGCGAAGTGGACACCGCAAGTGACGAACGGCGCGCGCCTCCTATATAAACATTACCCGACCACCTACAGATATATTATAATTATCATATTTATTAAATATAATACATTTAATTGCTTAATAAATGACACGGAGTTATGTTACAAATAAAAATAAAAGTTTATTTTTACGTTTTCTCTATTTATAATTTTTCTTTATTTAATTATTTATTTTTTGTTTGTTTTGTAAATATAACGATAATTATTATACACTGGTACTTAATGGAAATTAAAAACAATTATAGTAAACAAAATATATCTTATTTATTGTTAACATTTTAACATAAAGCAAATTAACAAAACACTCATAACTACACTCAGTAATAATTAATCCAAAAAACGTTAACAATAATCATTAACAAGAAAGTCTACTATTGGTTTATTTTAAAAGTTCTTTTTTATCAAATACATATATACATTCGAAGTTTTAAATCGACATAAGCACGGATGCACATTGCACATATTAAGAATACTTTTTATTGTTAATAAAAAAAAAAGTTACTAAAAATGG |
| **Chemosensory protein genes** | | |
| ApCSP1 | | TAGCCGCTGATAAATACACGGACGAGTATGCGTTTTGTTTCGACCGTGTTCGGAAGTGTCTCTGAGAAGTGCAGTGCTATTTACTTGACCTTTGCTACGAAGTTGTTTTTACACAATCACTAGAGTTGTTGACACCCACAACAAGCTATTCGTGCGAAACATGAAGTACCTTATTGTCTTGGCGTGTGTACTTGCTGGGGTGCTAGCCGCTGATAAATACACGGACAAGTACGATAACATTAATCTTAAGGAAATCTATGGCAACGAACGTTTGGTCGAGGCTTATGCCAATTGTATCCTGGGCAAGGGAAAATGTACCCCTGAAGGACAAACTTTAAAAGACAACCTCCAAGATGCCCTGGAAACCGGTTGCGCAAAGTGTACAGAGAAACAGGAGGATGGGGCTGGTCAAACAGTCGAGTTTCTAATTGAGTCCAAACCTGAAATATGGCAAGAAGTGACTAAGGCGTTCGACCCTACTGGCAAATATAGGAAGAAGTACGAAGATCGCGCAGCAGCCAAAGGCATTAAGATCCCTCCTTATTAAAATATACGTTATAAAGAATTATAAAATATAATTCTTCATAATAAAAAAACAGACACCTTCATTTATTTATATTAATATATATTCGTTTCATGTTCTAGAGCAGAAAAGAAGTAAATATCTGGAAATACGACAAATTGTGTATACTATCTTATTTATATTTTCCTATAATAAATTATACTTTTGACCTTCAA |
| ApCSP2 | | CGCGGAGCTAACAATCGCTACACCCGTGCCAAATAAATCTAAGAATGAAGGCTATATTTGTTGTCTGTTTGCTCGGCCTTGCGGGTGTTACCCTTGCAGCCCCTGACAAGTATACATCGAAATATGACACCATTGATTTGAAACAGATCCTGTCGAACAGACGACTCCTCGTTCCTTACCTTCTATGTATTCTAGATCAAGGAAAGTGTACACCTGAAGGAAAAGAACTTAGATCTCACATCCAGGAAGCTATTGAAACAGACTGCGCTAAATGTACCGAAGCGCAGCGTTCGGGCACAGATGAAGTCATCGGGCACCTTGTCGTAGAAGAGCCTGAATACTGGGGTGAATTGACAGCAAAATTCGACCCGACCGGTAGATATGTGAAGGCACACGAGGCTGAACTTACGCGTCTTAGAAAATCTGGTTAAACAATTATATTAAATTGTATAATAAAAGTAAAATATTTATAAATCAGATTATTCCGGTGCATTGATTTTTAATACAGGTTAGAAATAGGTTTATTATATCTACGCAGTTGAAACGGGTTTATTGTTAGTCTTTGAAAATATTTGTTGATACTTTGACGTCTATAAACTAAACAAAAAAGAGATACATTTTGAACGTTTTCACATTTGTAAAAAAAATATTTAGTAGGTTTAATAAAAACCAATTAATTTATCAAACAAAATTTAGATGATTTGTATACTTCCGTAATTAAATAGTTGCGTAAGGACTTGTGTTTTTAATAATTAATTGTAACAAAAAAACATAGTCTAATTATTAAATTTTTTTTAGTAAAAAAAATTTAATAATTAGA |
| ApCSP3 | | GGAGACGACTTAAAGAGATTCAACGTTTAATATCGCACTCGTGTATTAATACTCTTTGCCTCATAGCGAACGTATAAAGGTGATCGATCTGTCTATAAAATAAATTTATTTTCCAGAATTCATTATATAATCATGAGGGCGGTCATAATTTTGTCGTGTCTCCTTGCGTACAGTTTTGCCGCAGATAAATATAGCTCAAAATACGACAATTTCGATGTGGACACATTGATAGCCAATGATAGATTGTTGAAAGCATACATTAACTGTTTCCTAGACAAAGGTCGATGCACGCCTGAAGGATCGGACTTTAAAAAAACTTTACCAGAGGCAATAGAGACAACCTGTGAAAAATGCACACAAAAACAAAAGGCCAACATTAGGAAAGTCATAAGGGCAATCCAAGCCAAACATCCGAAACAATGGGACGAGCTGGTGAAGAAAAATGATCCTACGGGCAAGCATCGTGCCAATTTCGATAAATTCATTCAAGGAAGCTAAGTTGCTTACAATTTCAGTAGATCGAAATTTTGTTTAAATTTTTTTGGATTACCGTTTCAAAATGAATTTAATTTATACTTTTTGATTTCTGACATTTCAGTGTAATTTAGACCGTATTGTTTTTTTTTAATGAGACATATACCCATAACCGATTGCTCTCTGATTGTTAGCTTTTTAAAATTTAGTATTTCTATTAATATTTTTTTATGTGATTTAATTTATTACGCTATAGATTAATATATGAATGAACGAAAGGTATCAAATTTATTGTGTACAGTTGTTGAACTATCACTGATGTTGACTGAAG |
| ApCSP4 | | ATAAACAAAAGATCTGTGAGGGTTTACATTCTATTCTTTCATTGGCTGTACAACAAATAGTACCGTGGCACGGGTGCTTGGAGTAAAAGATAGATAGCCTCAAAGATGAAGTACATTTACGTCCTGTGTCTGGTTGTTCTGGCGAGCGTGTATGCAAAGGAGACATATACTACAGAGAACGATGACTTAGACATCGAGGCTCTGGTTAAGAACTTGTCGGCTCTGAGAGAGTTTGCTGGATGCTTCCTTGATACTGTTGAGTGTAACCCTGTGGCTGGAGATTTTAAAAAAGATATAGCGGAGGCCGTGGCACAATCATGCGAAAAATGCACAGATTCTCAGAAACATATTTTCAACCGATTCCTTGAAGGTCTTAAAGAAAAATTACCTACAGAATATGAAGCGTTTAAGAAAAAATACGATGCTGACGGTAAACATTTCCCTGCTCTGGAAGCTGCTGTTGCAAATTTTTAATCTTACATAATAAATGATCTCATTCATTTC |
| ApCSP5 | | GCCGGAAGGAAATGAATTCAAAAAAGCCATTCCCGACGCTCTGCAGACCAAATGCTCCAAGTGCTCGCCGAAACAACGTGAACTGATCCGTATTGTTGTGAAAGGATTCCAACAGAAGCTCCCTGAGCTATGGGAACAACTCTCCAAGAAAGAAGACCCACGCGGAGAATACAAAGACGCTTTCAATGATTTCATCAACTCCACCGATTAGATGCATGCCGATGAGATGTAAATAAGTTCACAAGGCTTAATAATAT |
| ApCSP6 | | GAGTGCAATAACGAATTTCATTCAATTCGTCGAAGATTAGTACGCCATCAAGAATGAAATTCGTTATTGCACTCGCTCTGTTCGCGGTGGTGGCTGCTCGTCCCGGGGATCAGTACGATTCGAAATTCGATGATCTCAACATAGACGAAATCATTAATAACATACATCTCCTTGAAGCTTACGTCGAATGTTTCATCGGTACTAGAAAATGCACGGCCCAAGGAAAAGAAATTAAAGAGTTAATTCCTGGAGGTATACAAACAGGTTGTGGTAAATGCACCGACAAACAAAAACCCCTTGTAGCCAAATTTATACACGCTACTCGCGAAAAATTACCTGAAAAATGGAACACCCTGAACGGACTGCACAATGGTGATGGGAAATACGACAAAACACTTGAAGATTTCTTGAATGAACACTATCATTAATTTGATAACCTATCAAAACAGATTCCTTACAATAGTCATTGTATTTTTTGTACAAATAACCCACTAGTTTTTATATTTACCATAACAAAAACCAATAGAAAAAATAACGTGGGAGATAAAATTTGTGTGTTACCTAACAAACAAATTTTATCTCCCACAAACAGGCTACCGAATCACCTATAAAGCAAGAAGTTGCTTCACACCCTACAGTGTTTTTTTTAAAGAATAAAAGTTCGTAATAA |
| ApCSP7 | | GGGGGACATTCAATTCGGTGAATCTGTACGTAATCCAAAATGAAACTCGTGGTTGCACTTGCTCTGGTCGCGATGGTGGCGGCTCGTCCCGATGACTATTCGAAATTCGAGGATATTAACGAAGACGAGATCATTGACAACGAGCGTCTCTTTCTAGCTTACCTCGACTGTTTCATCGGTATTGGAAAATGCACAGCTCAAGCAAGTGAACTTAAAGAGTTAATCCCTGAAGGCATACAAACATCTTGTGCTAAATGTACTGAAAAAGAAAAACCTAAGGTAGCCAAAGTCATATTTGTTGCTCGCGAGTCATACCCTGAAAAGTGGAATAAACTCAACGCAATACACAATGCGGATGGAAAGTACAACGAAGTGCTCGAGGAATTCCTGAAAACATACCATCACTAATTTACAAATCAAAAACATATTCAGTACAATAGTCCTTGTTTTCGTATACAAATAACCCAATATATTTTACTTTCACCCTTACACAATAATTATCAATACTGTAAAAATGTTTGTTTTTAGTAAACCACATAAATGCACATAAATAAATAAATACATGTTCATAATAAAAAAAA |
| ApCSP8 | | AGAGTTATAAGGAGAAGCTTGTATGTATTAACAACGCTCGTGTGCTTCGCTAGACCCAGAAGTGTTTAAAAGTATTGCTATAAATAGCGATAATCAAGTTATTATTCAAGTCCGTGGTTAAGTTTACGACCGGTATTAAAACAGCACAATGAAAATATCCGGTATCATTTTGACAACAGTACTGGCAGTGGTTGCTGCGGATTTTTACAGTTCACGATATGATGATTTTGACGTTGAGCCTCTTCTTGAGAATCCTAGAGTACTTCTCAGCTATACAAATTGCTTCCTCGATGAAGGACCTTGTACACCGGACGCCAAAGATTTTAAGAAGGTAATTCCAGAAGCACTGGAAACTACTTGTGGGAAGTGCACTCCTAAACAAAAAGAATTAATAAGGGCAGTGGTCAGGGCTGTGATGCAACAACACCCGGCAGCGTGGGATCAGCTCACTGACAAATACGACAGCGAGAAAAAATACAAAGAGGCATTTGATAAGTTTCTTGCAGGAAAAGACTAATTGCAACTCTTTTAACAGTGTACAGGTTTTCGCATATTAGAATTTATTTGAAAATAAAATTTAATTCCCACAGAGTATGAAATGTTTCTTTGGTATTTTTATGTTATTGCATTAACTTTATATTATTGATTATGTCTGCAGTGCCAC |
| ApCSP9 | | GCATTCTCTACTAAGACGCAGGCTTAAACGCTTCACTGGTTCGAGAAAACATAGTGAAATATCGAAATAATAACTCCAAGATCCGGGTCATTCGTTGAGGACTCACGACACAGTGCACGGAAAGTAAAAGTGTATTCGGGTCAGTCGATCACGTCAACAGACGGACACCGTTTAAAATGCAGATAATCTCCCTGCTTTTCGTATGCGCAATGATGCTGATATGCGGCGCCCAAACACAGAGGCCGCCAGTGTCGGACACAGCCCTCGAGGACGCACTAAATGACAAACGTTTCATCCAGAGGCAGCTAAAATGCGCATTGGGCGAAGCACCATGCGATCCTATCGGAAAGCGCTTGAAAACACTCGCTCCTTTGGTTCTACGTGGTGCGTGTCCTCAATGTACACCCCAAGAAACTAAACAAATACAGAGGACCCTCTCGTATGTGCAAAGAAACTATCCACAACAATGGGCAAAGATCGTCCGCCAATACGCCGGTTAAAAGGCTAAAATTTTTATATGTTGAGTTGCAGTAATTAAAAAAAATCAGAAAATAAGAATTCTAATGTGTATAGCTTTTTAAAGTGTATTTATTCTTCATTGTATCACAAAATTTTGTTATTTATTTCACAATGTGCATTTTATGGCATTACAAAAATATCATATCTAAAAAATTGAATGCGTGTAACCGTCACACGCGATGGAAGTAAAACTTCTTTGGCGTAAACATAACCAAAACATCTATTGTAATTGAATTCGTTACTTATCACATATATACATAATTAATTCC |
| ApCSP10 | | GTCGGTCCCTAATCGTCGGCCAGACGTTACCCTAATATTGAACGTGACGTATATATATAGTTTGTGCTGTAGACTTCGTTATAAGCGTTTCTAAATCGCTGGCAGAATGCAGATTGTAATTTTACTATGCGTATGTGCGGTTATATTACTGCCCTTGCCGACGTCAGCGGCGCCGCATATGTCGGATGCTCAATTGGATCAAACACTCGGTGACAAAGCTACTATACAGAGACACTTGAAGTGTGCACTCGGGGAAGGTCCATGTGATCCTGTTGGCCGAAGATTGAGGACTCTAGCACCGTTGGTGTTACGCGGCGCCTGTCCGCAATGTACGCAGTCAGAGACGAGGCAAATACGGCGCACCCTAGCGTACGTTCAGAGGAACTACCCGTGGGAATGGTCCAGAATCGTCCGTCAATACGGATGAAATGTTCAAAATACAGCAAGATTTTTTAAACGCAATTATTATTTACATATGCTTTTAAACTACAAGTTTATATAAAACAATTGCAGCACATTTCTATTATTTATTTTCGCTTACTATTTTTCATGTTATATCTACAATACTTGACC |

**Supplementary Table 5**.

| Gene | Nucleotide sequences |
| --- | --- |
| **Steroid hormone related genes** | |
| *Neverland* | SRX1142589 |
| *Spook* | TTGGCACGTATTTATTACACGCCATACTCCCCGGTGTGGTTCTGTTTACTCATAAAAATCTTATATCCAAATATAAGTATGAGTTCGTTAATCATTGTATTATTCGTTTTCGCATTGGCGGTTTATAAATTGCTTAGAAGGAAGACCGTGCGGTGGGTGAAAACGAATAAATATGGCGGAGTCGAGACGGCCATTCTAAGGACCGCACCGGGGCCCGTTTGCTGGCCCATTATCGGGAGTCTACACCTTCTGGGCGGCCACGAGTCGCCCTTCCAGGCCTTCACTGAGCTATCGAAGAAATACGGCGACATTTTCAGCGTCAAATTGGGCAGCGCCGATTGCGTGGTCGTAAACAATTTATCTCTGATCAGGGAAGTTTTAAATCAAAACGGCAATGTGGTGGCGGGACGACCGGACTTCCTGCGCTTCCATAAGCTCTTCGCCGGCGACCGGAATAACTCTCTGGCCCTGTGCGACTGGTCCAATCTTCAGCTGCGCAGGAGGAACCTGGCGAGGCGGCACTGCGGTCCCAAACAGCACACCGACTCCCACGCCAGAATCGGCACCGTCGGCACCTTTGAATCTGTCGAGCTAATTCAAACCCTCAAAGGATTGACCAGCAGATCGGACGCCAGCATCGATCTGAAGCCGATACTGATGAAGTCCGCCATGAACATGTTCTCAAACTACATGTGTAGCGTCCGTTTTGATGACGAGGACCTCGAATTCCAGAAGATTGTCGATCACTTCGACGAGATATTCTGGGAGATCAACCAGGGCTACGCTGTCGATTTTCTGCCGTGGCTGGCGCCGTTCTACAAAAAGCACATGGAAAAGCTCTCCAACTGGTCCCAGGACATCCGATCCTTCATCTTATCGAGGATCGTGGAGCAACGCGAAATAAACTTAGACACGGAAGCTCCGGAGAAGGACTTTCTGGACGGCCTCCTCAGGGTACTACACGAAGACCCGACCATGGACAGGAACACTATTATCTTTATGCTCGAAGACTTCCTGGGGGGGCATTCGTCTGTTGGTAACTTAGTCATGCTGTGTCTGACCGCAGTCGCCAGAGACCCGGAGGTCGGCAGAAAGATTCGTCAGGAAATCGACGCGGTGACCAGAGGCAAAAGACCCGTAGGTCTCACCGACCGCAGCCATTTGCCGTACACCGAGGCCACCATCTTGGAATGTCTGCGGTACGCGTCCTCGCCCATCGTGCCCCACGTGGCCACCGAGAACGCCAACATATCCGGGTACGGAATCGAAAAGGGTACTGTCGTTTTTATCAACAACTACGTTCTGAACAATTCCGAACAGTACTGGTCCGAGCCGGAGAAATTCGATCCGAGCCGTTTCCTGGAGAAGACCAGGGTCAGGACGAGACGCAACTCCCAGTGTGACTCCGGCCTGGAGTCCGACTCGGAGAGAGCGCCCGTCGGCAAGCCTGACGTCGAGAGGGAGATGCTTTCCGTGAAAAAGAACATACCCCACTTCATACCCTTCAGCATTGGTAAGAGAACGTGTATCGGTCAAACTATGGTCACGTCGATGTCTTTCACGATGTTCGCGAACATCATGCAGTCCTTTGAGGTCGGCGTCGAGAACATCAACGACCTCAAACAGAAGCCGGCGTGCGTGGCCCTACCCAAAAACACATACAAAATGCATTTGATACCTAGGAAGTAGCATTGGCAATAATGGCGTTCTTCTTAAATACTTTTAAGTGTCTTTGTTACTAGATGTTTCACAGAGCAACGTTTACAAAAAACTTAGCAATGGCAGTGTCAAAAAGACTGGCAATAGATTGGCTATCAACAAAACTTATGCTTCGAATGTTTTCAATCGATTTTTTGCATTTATTGGTGAACATAGCACACCTGGTGTTAAGTGGTAACTGGAAACCATAGAGGTTATCATTGTGGCCTTCCTGTGATTGCGTCGCCTCCTGCGAAACCTCACGACTCAAGACCGCATTCAACTGAATAATATTCTACGCGCGTTTAAGTAACGGTAAGCAGAGGCTTGACTCTGCCCCTGGCATTGCTGATGTCCATGAGCGACGGTCACCACTCACCATCAGGCGGACCGTATGCTAGTCTGCCTATACGGCAATAAAAAAGTAAGTCACAATAAAATTGTCGGCCATCTTGAATAAATTCACCAAAATAAGCTGAGTCTCTTTTTCAACACAAGAAATGAATTGAACAAACATTTCGAAGACATTCAAAGACTACATAGCTCTCTTAGCAACGCGCAGTGATGTCTATGGACTGCTGCATCGACGGTAAACGAAACTTGAATACTAATAAATTGTTATTGTTCACCTAAATTACTTCGTTTGAAAGGTTTGTTATCGTTCATAAAAACCGTGATTTAACTTTTAAAATAGGCTTTTCTAAATTTAATCTGATCATAGAAATTATATTTTATTTCACAACTTCACATGTAACTGTAATGAATTCAGAAAACTTATTTAGAAATTTGCGATTTTTTTTTAAAACTAAGAAAAAAGCGCGCGTTAATTGCAAACGCACACTAAAACAAAACATTGAATTAATAATTGTTTTACCGTGGTTGTTCTGGTCAGGAACTTGAATGAAAGCAATAAAATTATTATATCGTCATCGATCCTTGATGCGTGTGTAAGTTTTTAACTTTTTCCTGTTAATCTTGAATTCTGTGAAAATTACGATGAAACATTTCAATAAATACAAACTAAAACCATGTGACCAAATAAAAACGGTCAAACGAAAGAGACAACCTCGCTCAGAGTACTAAGTGTGAGAGGGACAGTAACATGATAAACATCTAGTAACAATGACACAGTTGATTTAATCAAGTCAAACGTTCATGTCGTCTCTTTTTTTAGCTTTTGCTAAGCCTAAAGAGTGCGAGAGAGACGGATAAACGTTAACTTTAAAATTAGTATTTGTGAAAGCAAAGAACAAAATTAATTAATCTCGTATGTATTTTTTTGTGTTCTTTTTGTTGTTTTAATAACATTTTGAGTTCAGTGATAAAAATATGAATTTGCAATTATTTGTAATAAATCTAGAATTGCTTCGAATCAATATTAAAGTTTATTTTATAGTGCATTTCGTGTATTTTTGAATATTATTACACAAAACGGACACAAGAAAACGGACAGAGATTTTGCTCAAATCTCTATTTATTTCTTTTAGAATACATACCGTATTTGTATAAAAAAAACCCAATACACACTTGTAGTGCTAATATTGCCAATATAAGCCCTATTTAGTGACATTGGTACTAAAATTTAACGCTAAACTTTAAGGGGGTTTAGTTTATTTGGGTGGCTGGAGATGTCGATGGTGACGCTTACGATCAAAACGAAAGTCTGCATGCTTACTGATTAATTGAAAAGGTCATTTTGTTTTACGTAAAACCTAATAATCGAGAGAAAAATATGACTGCACATTTTGTTCAAACATAATATGCAACAATTGCTGTTTTTGTCTATTGAAATTCTGTATAATTTATGTAACTAAATACGCACATTATAACACCGGTTCCGGAAGCTTAGGTTGGTGATTGACCAGATTTCCGCTTCCTATAAAAAAAACTATAATCATACCTTTACATTACAATCTAGTAAAATCTGAGCACCGCTAAGGTGTGAGAAGTCTCATTAGAATCTTAATTCCCAAAAATAAATAAACAACCTAGTAGGTACTAAAAAGATCAGTCTCCGCTTACGTATTTATTGGCCTATTGACTAAAGGAACGCTCCTCATAAATCCTTGATTAAAAATTATATTTTATCTGTCTCAAGTATTAGATCGCGAAATTTTTGAGCTATGCAAAAATTAGTCATTTTAAATTTGACGTTTTTTTTATTTAGTCTTCTATTAGATTACTCGGCTTGGCCTCTAAAGTTTTGAAGAATTTTTGTATTTTAGGTAAGTAATATTTTCTGGTAGGTATTATTACAAAATATTCGTAATCGGTACACGTGGGTGGCGGAAGGACGTAAAGTTTAAATCTTTTTAATTATTTTTCACTATGGCAATTGCCTTCACTCGAATCCATTGTTGGTAGTGTTGTGTTTATTAAAATATTTTAATATTAATATGACTATTTTAAATGAAAAATGAATGAATGAATGAATGAAATGAAGCGAATGAATGTAATTGATTTAATTGAAATTGTAATTCTTTAGGATGTATGTAATGAAACGAGATGAGATAGCGTCCCAGTAAAATTGTCGAAGTTCACTAAGACTTCAGTGGGACTTTTTGGAGGAACTCTAGCAGTCACGTCCGGCGACTTTTACTGGTGGTAGGATCTCTTATGAGTCCGCACGGGTAGGTACCACCACCCTGCCTATTTCTGCCGTGAAGC |
| *Phantom* | GGGGGGGGCAGTCGATAGGCGTCACTGCACGTGACTGCTGGTGCGTCCGAATCTAATAGTTGTAGTTAACGTGGTGACGCTGCGCTCACTTCATACCGATTATTGGTCTCTACTACATATTTGGCTTACTCGTTTTAACGATACCCAACTAATATCAACTGTATCTATCAAGTAATATGGACCTTTATTTTATTTGGCTGGTAACGTTCGTGGCTGGGTTTTGGATTTTCAAAAAAATAAAGGAATGGCAGAATTTGCCCCCCGGACCTTGGGGGTTACCTATCGTCGGTTATTTGCCTTTCATTGATCGCTATCATCCACATATCACCTTGACAAATTTGTCTAAAACATACGGAGCTATTTACGGTCTCAAAATGGGCAGCATATATGCTGTAGTGTTATCTGATCATAAACTTGTAAGAGATACGTTCTCAAAAGACAGTTTTTCTGGACGAGCACCTCTTTACTTAACACATGGCCTTATGAATGGAAATGGAATTATTTGTGCCGAAGGCGGTTTGTGGAGGGACCAAAGGAAATTAATAACATCGTGGTTGAAAAGTTTTGGAATGAGTAAGCACAGTGTTTCCCGAGAAAAATTGGAAAAACGAATCGCTTCAGGAGTATACGAAATTTTGGAAAATATCGAAAAAACTTCTGATGCTGCCTTGGACCTTCCTCATATGCTGACGAATTCTTTAGGAAACGTTGTCAATGAGATAATATTCGGCTTTAAGTTTCCACCTGAAAATAAAACATGGCAATGGTTTCGTCAAATACAGGAGGAAGGATGCCATGAGATGGGAGTCGCAGGTGTTGTAAACTTCTTGCCCTTTATACGCCATGTTTCGCCATCAACACGAAAAACAATTGAAGTTCTAGTCCGTGGACAGGCACAGACGCATACTTTGTACGCAAGCATGATAGATAGACGAAGAAAAATGTTGGGCTTAGAGAAGCCTAAGGGAGCCGAATATGCTCCTCACGAAAACCTTTTAAAACTATATCCAAATGGCCATATCAAATGCATAAAATACAGCAAAGTCTCTCCGAACACCGAGCATTTCTTTGATCCCAATACTCTTATTCCGACTGAAGGAGATTGCATACTGGATAATTTTCTGTTGGAGCAAAAGAAACGATTTGAGAGTGGAGACCCAACGGCACTGTATATGAGAGATGAACAGTTACATTTTCTACTAGCGGATATGTTCGGTGCCGGACTGGATACTACATCGGTGACTTTAGCTTGGTTTTTGCTATACATGGCTTTGTTTCCAGAAGAACAGGAAGAAATACGTAAAGAAATCTTATCCGTATATCCATATGACGATGATGTTGATAGTTCAAGGTTACCTCTTCTTATGGCAGCAATCTGTGAAACTCAGAGGATTCGATCGATTGTTCCAGTGGGAATACCCCATGGTTGTATAGAGGACGCTTACTTGGGTAACTACAGAATCCCAAAAAATGCCATGGTGATCCCATTGCAGTGGGCTATTCACATGGATCCTAATGTTTGGGAAGAACCAGAAAAATTCAAACCGCGTAGATTTTTGGCTCAGGATGGTAGTCTACTTAAGCCTCAAGAATTCATTCCGTTTCAAACTGGTAAGCGGATGTGTCCGGGTGACGAACTGTCCCGTATGTTGTCGTGTGGCCTCGTAAGTAGACTATTCAGAAAGCAGCGTATTCGACTCGCATCAAAAATACCGACAGCAGAAGAGATGCGTGGAACCGTCGGTGTTACGTTGGCACCTCCTCCGGTGAAATACTATTGCGAACCAATTTAATGACTAAAATTTTATTTGAATTCGCTTTTTATTAAAGTAGCGTTCAATAACATTAGTTGTAGATCTTTATGTAACTTTTGTTTCACGGAATATACCTTTTATCATTTGTCGGTTT |
| *Disembodied* | AAAGTTACGGTGCGGTCCGGAATAATCTTTTGTTCAAATACATAATTCCCAATGGACGGTGTATTTTTTGTAAGTCTATTCTTTGGCTCAGTGATTCGATGCAATCCGCACACATCGCTTAATGCTTCAGTTTAGGAAACATGTTTGTTAGATTAACAGTTAAAAATAATATTCCCTACCGGGCGAGGAAATGTGTTTACAGAAGAGCGTCTGAAAATTTTGTGGGTAGTGAACATGCCAGTAAGGTCAATGAACAGGGAGATAATCTCATGAATTTCGAAGATATACCGGGACCTCGAAGCTATCCCATTATTGGTACGCTTCATAAATACCTACCTTTAATTGGTGACTATGACGCCGAAGCTTTAGATAAGAATGCAATATTGAATTGGCGACGTTATGGTAGCTTAGTTAGAGAAAAACCGATTGTCAATTTAGTCCATGTTTATGATCCGGATGACATTGAGGCGGTGTTCCGACAAGACCACCGCTATCCAGCGAGGCGCAGTCATACCGCTATGAACTACTACAGAACCAACAAACCGAATGTGTACAACACTGGAGGACTTCTTGCGACCAACGGACCCGATTGGTGGAGACTCAGAAGCATATTTCAAAAGAATTTCACGAGTCCACAAAGTGTTAAAACTCACGTCTCTGACACCGATAATATCGCAAAAGAATTCGTTGAGTGGATCAAAAGAGACAAGGTGTCATCTAAAAATGATTTTTTGACGTTTCTAAATCGCCTTAACTTAGAAATCATAGGTGTAGTAGCTTTTAACGAACGTTTCAATAGTTTCGCTTTGAGCGAGCAAGATCCCGAGAGTCGCAGCAGCAAAACGATAGCGGCAGCATTCGGATCAAATTCTGGGGTCATGAAGCTTGATAAAGGATTTCTCTGGAAAATGTTTAGTACTCCACTATACAAGAAACTCGTGAATTCTCAAATCTATTTAGAAAAAATTTCCACAGATATATTGATTAGAAAGATTAATCTCTTCGAATCCGATGATAGTAAAAACGATAAATCTTTGTTAAAAACATTTCTTCAACAACCCCAGCTGGATCACAAGGATATCATGGGAATGATGGTTGATATTCTAATGGCTGCAATCGATACAACAGCCTACACGACAAGCTTCGTATTGTACCATATAGCCCGTAATAAGAGATGCCAGGACGAAATGTTTGAAGAGCTACATACATTATTACCAAAGAAAGATGATGAGATAACCGCTGACGTTCTTTCTAAAGCTTCATACGTGAGAAGCAGTATTAAAGAGAGTTTGAGACTTAATCCGGTATCGATTGGAATAGGTAGATGGCTGCAGAAGGATATAGTTTTAAAAGGATATTCCATTCCTAAGGGAACTGTTATAGTGACGCAGAACATGACGTCATCAAGGTTGCCGCAGTTTATACGGGACCCCTTAACCTTCAAGCCTGAACGTTGGATGCGTGGTTCACCGCAATACGAGACCATTCATCCGTTTCTGAGCTTGCCTTTTGGTCACGGGCCCCGTTCGTGTATTGCAAGAAGACTGGCCGAGCAAAATATTTGCATTATCTTAATGAGGTTAATTCGTGAGTTTGAAATCCAATGGGCCGGCGAGGAGCTTGGAGTGAAGACACTGCTTATAAATAAACCAAACAAACCTGTATCACTAAACTTTATTCCAAGAAGTAGTTAACGTTAATTATTAGGAAGTTTTATTATTTTATTACGTTCAACAATGTATAATAAATATCGAAAAGAGAAAATTGTTGTTAACATTTTTTTTATATTGTTTATTTTATTTTAAAGCACTAACTGATTGTCGATGCTTGTAAGATTGCATATTTAGTCTTAAAAGACATCGTTTCGCTGTTTTTATTGTTTTTGGCCAAAGCATCAATTACTCTGATTTGAAGTCGAAGTCACAATTTCATTGCAATAGTGTCTGTACATTAAAAATAAAACATTTTTTTGTATTTTATTATTATTTATTAAACACGTTTTGTTAAATAAAAA |
| *Shadow* | TACATTATTCACTGAATTATTTCCATTTTAAAAAGAGGTCTTCAAATGGCTTAGCAGAATAAAATAAAATCTCGTTTAGTTTTCCAGGAACCGTTGCCGCATTGTGTTCTCGGTTTATTAAAGTGGTTACTTATATCAGGATCCTCTTTAATTTAAAAATGCATCGTTTCCCGTCGATGTCTTCCATTAGATCCGCTGTTAGGAGTCGTAACAGTAATCGATGTTCAATGTCAACTAAGCCTCATAAATCTCTACGTACCATAGATGAGATGCCACATAAGAAATCATTACCAATCATAGGAACAAAATTTGACTTATTTTCTGCAGGTGGAGGAAAAAACTTACACAAATATATCGACATGCGTCACAAGCAATTAGGACCTATATTCTACGAGAGATTAACAGGAAAGACAAAACTTGTCTTTATCAGCGACCCAACTCACATGAAAAGTTTATTCTTAAATTTAGAAGGAAAATATCCGGCACATATTTTACCAGAACCGTGGGTACTATATGAAAAATTATACGGATCAAAAAGAGGACTTTTCTTTATGGACGGAGAGGATTGGTTGATTAATCGACGAATTATGAACAAACATCTACTCAGAGAAGATTCAGATGTATGGTTACGAGCTCCTATAAGAACAGCTGTGTTTCACTTTATATGTAACTGGAAACTTAGAGCTCAAAGTGGCAACTTCTCTCCTAACTTGGAATCTGAGTTTTATAGATTTTCCACTGACGTTATTTTAGCAGTTCTTCAGGGGAATAGTGCACTATTAAAGCCCACGCCAGAATACGAGATGTTATTGTTACTGTTTTCTGAGGCTGTCAAGAAAATATTCTCAACAACTACAAAATTATATGCTTTACCCGTAGAATTCTGCCAACGATGGAATTTAAAAGTGTGGAGAAATTTCAAACAATCCGTTGATGATTCCATCTCTATCGCTCAGAAAATTGTATACGAAATGTTGCACACAAAAGACGCTGGTGACGGTTTGGTAAAAAGACTAAAGGATGAAAATATGTCTGATGAATTAATAACAAGAATTGTTGCTGACTTTGTCATCGCCGCTGGAGATACGACAGCTTATACATCACTTTGGATATTATTTTTACTTTCAAATAATACTGAAATCTTAACTGAAATGAACGACAACGACCAATACGTGAAAAATGTTGTTAAAGAGGCAATGAGATTATATCCTGTAGCACCATTTCTTACAAGAATTTTACCCAAACAATGTGTTTTGGGACCTTATTTGCTCGAGGAAGGGACTCCAGTAATAGCTTCAATCTATACATCGGGTCGAGACGAACAAAATTTTAGCAAAGCAGACCAATTTTTGCCTTATCGATGGGACAGGAACGATCAACGAAAGAAGGATTTAGTAAATCATGTACCATCTGCCACACTGCCATTTGCCTTCGGCGCCAGATCATGTATCGGTAAAAAGATGGCTATGCTACAAATGACAGAATTAATCAGTCAGATTGTGAAAAATTTTGACCTGAAATCTATGAACAATTCTGATGTGGATGCTGTAACTTCACAGGTGTTAGTGCCGAACAAAGACATCAAAGTTTTAATACTACCGCGTTCAATTTCTAAGTAATATATTAGTGCGTAAAGGGTGTCATATTTTCACCTATTTTATCTATTTGATTAGAAATAATGAATCTAATAATTTTAATAAATTTGGACATGCTAGGATAATATTTAATGTTGAAATTAAATTTATATAATGAATAATTCATTGGATTTGAATACGACATAAATTTATTTCTAATTGTATTATTTTATTTTATTTTATGGACCGACATTTAGTGAAATTGTTATCAGGCAACTTTCAAATTTAAAGGTTTAGCAAATAAA |
| *Shroud* | CCTTAATTTTATATGATTAAAAGGAAAAGTTTATTGCTTAATAATTAAAACAAAAAAAAAAAACATTCTAAATAGATATCTTTTTATAATTTTTAATTTTTGTAATTTTTTTTAAGTTTTTATAATTTTTTACCTTTTTATAATTTTTAAATAAAGTTGGTTATTACCTATTATATCTATAAGTTATTCTGTTCATTGGTGATCAGGGAAGCTTAGAAACTGTTTAATAATCCATTTATGAATGAATAATGGCAAAGGCAATCTCATTAAATTATAATAAAACATATATCTCCAGGATTCCACTTTATACATTAATTTCGGAGTTTCATCCAACATAGCCTTCATAAAAGTGTTCAATATTTTCATATCATGCATTGAATCAAATTTCCCCTCGCCAGATGCCAGCTCCAAATAATTGTTTAGAGATTCAATTTTATTTCCGTAAAACTTTCGTTGTTCATCGTTTAAATGTTCAACCATTGCTTCTCCTTTTGTTTCTTGCCCGAGCAGAATGTTACTAGAGCCAACGAAGCCACCTGGTACAAAGGCAATGACAGCTAATCCGTGCTCGCTGTGCTCAAGATGTAGACATCGTGTCAACGCCAAAAGGCCAGCTTTGCTTGCACTGTATGCAGCGAAGGCGGGCAAAGGCTGCAGGCCACAATGGCTACCAACGTTGATTATACGGGGCTTAGGTTTGCTTGTAGTTTCGATTGCAGTTTTTCTGATCTCTGGTAAAAATGCGGAAATGACTCTCATCGCGCCGAGCAAATTCACATGAACCGGGCTTTCTATCATCGATGCTGTCATCCATTCGTACTTTCCTACTGTCATAACTCCAGCGTTGTTGACCACGGCATGAAATTTATATTTAGGATTATCTTTCAAGACTGTAAGCACGTACTTCCGGAATTCTTGAACGCTTTCAACTCTAGTTACGTCTAAAGGGAATGTGTGCGCACACAATTTTTCGAGGGCTTTCGCTGCTTCGGTTTCTATTCCTTTGTGCATACCAGCTATTGTGATGAAGCCTTCTCGGGCTAATCGAGCTGCTACTGCCCAACCGAGTCCACTATCACACCCTGTTACAGCTACAAGACGGCTAACGGACATCGCGCTGACTATTTAGAATATTTAGACTTGTAATGAAAAATTCTAATAAAATATTCGGCTACGACGTCGTTCCGATATGGTGTTGCTGTCTTAAGAACTGAAAAGACACTGACGTTTGTAGACAATTCCCGGCGGTCTGTTGCATATGAAACGGG |
| *Cyp6u1* | CTGGACGCCGCGCTGCGTCGGCGAAGACTCAGGTCCCCGCATCTCACAGCGCATGCTCGCCTGTATTCTGTTTACAAATTACGATATGCGGGCGCGCTGACTATTTTGCGGGGATTCAAACAATATTTTTCTTGCTTATCCCGTTATATGTGAATCAGTGACAATGTGGTTAAGTGTATTATTAGTGGTGATTTTACTGTTTTTGATTCTGTTGTTAACCGTGTTTCACTATACGACTAAAGGAAGAAAATATTGGTTGTTACGAAATGTACCTTACAGAGAACCATGTCCGTTATTTGGAAATTTTGGAGCAACGTTCACTATGAGGAGAAGCTACACAAAAATGTTGCAATTTTTTTACGATAATTATTATAATGAGAAATACGTGGGAATTTTTCAAGCACGGAGGCCGACATTAATGCTCATAGATTTAGAACTGGTGAAAAATGTATTATCTAAAGAATTTCCGAATTTCAGCGATCGCATATCTGTTACGACCGATACGCAGCGGGAACCATTGCTTCGGAATTTAGCTAATATGAGTGGAGCTGAATGGAAAGCTATGAGGCAGATTGTAACGCCAACATTTTCATCGGCGAAAATGAAAGCTATGTTTCCATTGATAGCGGAATGTGCTCAAACATTAAAAAATAGTCTACTTAAGGAGTCTCTCGCGGAAGTTAATGTGCCCGATTTTATGACACGATTTACTACAGACGTAATTGGTAGTTGTGCCTTCGGAGTCGACCCTGGTTCTCTGAAAGATCCCGAGTCTCCATTCTTAAAAATGTCGCAAAAAATGTTTAAAATTGATCGTTCTACTGTTTTAAAACGCTATTGTAGAACGTTTTTCCCAAAGCTGTTTAAATTTTTGAATCTCAGAACATACTCAAAGGATGTGGAAACGTTTTTCACAACTATTATTAAGAAAGTTCTCGATGAAAGGCGCGCCACGGGCGTTCAAAGACATGACTTCCTACAGCTTATGTTGAACGTTCAGAAAACTGAAACAAGTTTCGTTATGACCGACACATTGATAATATCGAATTCGTTCATTTTTATGCTCGCTGGACTAGAATCCTCGTCTACTACACTATCGTTCTGTTTATATGAATTGGCTAAAGATAAACACATCCAAGATGATCTCAGAACAGAGATTGTTGACTGCTTAGAAAGGTATGGTGGTATAAATTATGAAGCAGTGTGTTCAATGCGCTTGGTCAACCAAGCTGTACTTGAGACTTTACGACTTCATCCACCGACGCCGCTCACGACACGGCTTTGCACCAGTGCTTGTACTCTGAATGGCACAGATTTAAGCGTTAGAGTAAGAGATCCCGTGTTAATACCTTTGTATTGTATTCAGCGAGACGCCCAACACTTTCCTAATCCTGACAAGTTCAATCTAGAAAGGTTTAAGGAAACAAATCCTCCTGGATTTTTGGCTTTCGGCGAAGGACCGAGGAGTTGTCCAGGCGCACGATTTGCTCAATTGACAGTTGCCGCGGCTCTTGCGGCTTTGCTAAGTAGTTTTGAAATAGAACCTTGTTCCATGACAACTTCCACTATAGTCTATGATCCTAGAAGTGTAATGCTTAAGAATAAAGGCGGGATATGGCTGAAATTTGTACCTCTATAAATAAAAAATAAGTACTTAAATGTAAAGAAGTTCCAAGCATGTTAGTTTCTGAAGATGAATAAATAACGCTTTCCTTAATTAATTATAGTTTCAGTGTAGGTACTTAAATTTTTATCAATAACAATAAAACTATTTTATCACTTAAACTTGTTACTAATTTACTCATATTAATGTATCTTTGCAAAAAACATGTCGATAATTTGTTACCTTTTTAAAGGGACAGACAAACAAACATACTTCGTAATCACTGAACGCGTATAATTTTTTTCACGAAATAAGAATCCTATGTGATTCTGTCTCAGAAACAATCGGTATAAAAAGAAGGTTATTTGTTACCGCGTAAAAAAGCAACAGACCAATGAACACACTTGTGTTCATAATGATTCTATAATTATAATTCACTTCCGGTCGTTGTTATAATTTTTTGTGTCATTGTAATATAATGGTTTTACAGATGTGCGACGGTTTGTATAATGGTGTGTATGTACAAATAAATGTTTTGTTTGTTACATGCATTGATTGTATCGTTTGATGACCCCGAAAGGTATTGTTATGAAGTATTTCGTGGACGTCACTATTCGTTCATATATATATGTTACTTAATAGAATGTTACAGTATTTTAACATTTGGACCTGAATCGTAATATTTATTTTTTAAGTAGTACTATAGTAACTATTGTAGATGTATATTAAATGTTATATGAAATAAATAAATATGTAAG |
| *Torso-like* | GTTTAAGCAACAATTCTGAAGTTTAATGGAATTCTGTGAACGGTGTAGCTAGTCGGTGTGATGGTGCGGACCCTCACAGTTCTGCTGCTGGTGGTAGCTCTGTGCCGCGCTGCCACCCATGACTCAGAGCTTGGCGTCGGGAAGGCCATCAACATATTTATGAGATATGGCTACCTCAGCATATGCATGCGAGTTGTTCCCCGGAACGACACAGAGAACTGGGTTTTCCGGGAGCCGACAGTCAGCGTTTTCCGGGACGTGGAGCAATACAGCGTCGCGCCCAAACCTCGCCAGCCGAAGACACTGTTCGACGGAGACTTCCACATGGAGTTCTGCGATAACCTGAAGCAACTGCTGCAAGCGTATTTCAGGGATTTCACTTTTGAAAGGCTGGAGCGTCCATGGCGGGCGTTCACGGCTGGCTGGCCCACAGACATAATGGCCAGAAACTTGGGGATCAACTCCTCGTTCATCAATGGCGACCACTGCTACGTGCTCGTCAGAGTTTCCAGGTTCCGCGAAACGGGAAAACTAAAGGACTTGCCACAGAACATAGGAGTTGAGGATGTGGTGTTTGACGCCATCCAAGATTCAATCATTGGGGATACGGCATCCATTGCCGATTTCATACGCAAATACGGATCGCACTACATTGCATCCTATATTACTGGTAACTCTCTGTACCAGGTGTTTGTCTTCTCCCGGGGCGTGTACTCCAGGGTCAAAGACCGCGTCAAGTCTAACGGGGTCGGTGACCTATCTGTCACTGAGCTAAACAATTTCTTCTCGCCGTTCTTCGCTGAACACGTCGGAGTAGTCAAGGTCGCCAGCGGTAACAAAACAGTAGAGAGCTGGGCGACGAAACGTCTAAGGATGCATTACTATATCTTCTCATACCCGAGTCTCCTGAAACTCCACGGGGAACCGGCGTTACTACGCAACCTAGACACGTTATTAGGAAATGAAGCTCTATTGCAATTGGAATTAAAGACATTATCGCCCGCATTCAAAGACGCGAACAAAAGGAAGTGGTTCGAAGAGGTCATAGACAATTATTTAAAACTTTGGGAGAGTAATATGTGATGAATTTGTTTTAATTGATGAAATGTACATAGGAAATGTGGAACAATCATTGTGTGCCTTATCATTAAGTAATAAAATATATTATATAAGTAAGTTTTCCTAAAAAAAGATTATCTATTCCGCCATTTTGTTTTAACATCCACCATGATAGTTTTAAAGAGATAAATAACTATGGCGCGTAGATGTAAAAAGCGCCATCTTGTTTAAAAGACAAATTGAAAAAGTGGCTGGTTTTAAACAGAGTTAAGTGTGGTATTTTACGGTGTTGTTTTTTTTAATCTTTCCACTTGCT |
| **juvenile hormone related genes** | |
| *Farnesyl diphosphate phosphatase* | GGTGGGCTGTGAGCTCATCCACCGTTCTAGGGAATAAAAAAAAAAGTACCCACTCAAAAAGATTAAAATAATTGAAATATTATGAATGAACATGCGACGTCTGATTCGTAGATAAGTACACAGAATGTTTAATTTTGAGATTTAGCTTAGTTTCACAAATACATATTCTTCATAGCTAGAGGGGAAATGTATTTGTCCTACTAGTTAAATCATTTTTTCCTAATAACTTAAACAATTTACTGAATTAGAGGCACAATACTACCAAGAGACGCCGCGTAGTAATCTGGTCGTATTGTGTGTGACAACATTTCTTCTTTGGTAGTGTTCGTTAAAACTAATAAAGTGTTGAAACCAACTGCTTTCCCTAGAGATACATCTTGGGCGATCATGTCACCGATAAAAAGAACTCGACTAGGATCGGTGATGCCGGCTCGCTTCATAGCGAATTCACCGAAAGCTTTTCCTGGCTTCCCAAGCAGCACGGGCTCTCGTTTCACTTCTGCCGTAACCAGATCTGCAAAAACTCCCGTCCCTAAAGCTAAAAGACCAGGTTTCATTGGCACCGACCTATCAGTCGCTCCATTTATGAAGAGGACCTCAGGTCTTTTCAGGTAAGTTATGGCTCGGTACATTTTCGGCAAGTTTATCTTAAAGTCGCTGTCAAAAACGACGGCTCCTATCTCTTCATCGTCTTCTAAATACTGGATGTATTCGCCATAGTACTCCGGCCCTAGATCTGGACCTTCCTTGCATTTGAATCCGTGAGCTTCTAAGACTCTTTTGGTCTCAGTACAGGTTACACAGTAAACAGTTTTATTGAATGTAACTGATTTAAGGTACTCTGCCACTGCGATAGATGGAATGATCAGGCTCTCAAAGCCGTTGTCGATGCCGGCTGCTTTGAATTGAGCTTCATAATTTGCTCTGGATCGAATGCTATTATTGGAAACGAAATTCACTGTCTTCCCGCGTTTCTTCATCTGTTTAAAGAACTCTCCGACTCGCGGCAAAGAATCTTGAGTCCATATAACACCATCGCAATCGGAGAGGACATGGTCGAAGGAATCTAGAAATTTATGCAAGTCTTCAACACTTAAATCTAATAAATGTTTAGATTCAGTACCCATTATGTTTATTTAACACTTTGAGACGTTTTTTTTGTATAATATTAAAACACGTGTGCACTTAACCTTACTGTGTG |
| *Aldehyde dehydrogenase (NAD+)* | GAACATTGGAAGCGTCGCGTCGTATGTTTAAAAGATAAAAGTATTAGTCCACTCACGAACTCGGCGGTACGCAGTAAACATGGTCCCGGAAATTAAATACACCAAGTTGTTCATTGACAACGAATGGGTTGATGCGAAAGATGGCGAAACATTTGATACATTCAGTCCACACGACGGATCAGTCATCGCTAAAGTAGCCAAAGGACAAAAGGCTGATATTGACGCCGCAGTGGCAGCGGCGAAGCGTGCGTTCCAACGTAACTCAGAATGGCGGTTGGCGGATGCCTCGCAGCGCGGAAAACTACTTTACAAGTTCGCAGATCTTATTGAACGCGACTTCGAATATCTTGCGAAACTCGAGTCATATGACAATGGCATGGTTTATAAAAATTGCCTTAGAATACTTTCAGGAATTCCTGACAGTGTAAGATACCTAGGCAGTCTGGCGGACAAAATCCAAGGCGACACAATACCAACTGATGGCGATTCATTCAGTTACACATTGAAACAACCCGTTGGAGTGTGTGGTCTCATTTTACCCTGGAACGTTCCCATCCTGATGTTTCTGAACAAGGTCGTAACTGCCTTAGCAGCAGGATGTACAGTGGTAGTGAAGCCGGCAGAACAAACTCCATTAACTGCTTTGGCATTGGCCGCACTGTTGAAAGAAGCTGGCATACCTAAGGGTGTAGTCAACATCGTGCCAGGATTCGGAGAAGATGCAGGAGTGGCGCTCACACATCACCCTGATGTTGCCAAAATTTCCTTTACAGGATCTTTGGAGGTAGGCAAACTGATCCAAAAGGCAGCTGGAACAAACAATTTGAAGCGTGTCACTCTCGAACTCGGAGGCAAGAGTCCACTAATCATAATGAATGACGCCGATTTAAATTTAGCCATCCCTTATGCTGGCAGCGGTGTATTTTTCCACCAAGGACAAATTTGCGTGGCTGCTTCCCGCTTGTACGTCCAGTCGGGTATTTACGACAAATTCGTGAAGGGGGCCGTTGAATATGCAAAAGCTATCCGGGTCGGCAATCCTTTTGACGAATCAACTGTAAACGGACCACAGATTGACGAGTCTATGATGAATAAAGTTTTGGGCTTAATAGAGACCGGAAAAAAAGAAGGAGCGAACCTACTGGCAGGTGGAGAACGTATTGGTACCGCCGGATATTATATCCAGCCTACTGTGTTTGCAGATGTTAATGATGATATGACTATTGCTAAGGAAGAGATTTTCGGTCCAGTCCAGAGCATTCTAAAGTTCGATACGTTGGAAGAAGTGATCGATCGCGCCAACAATACAAATTACGGTCTTGCAGCTGGAATCTTCACTACAAACCTTAACAACGCATTGCAGTTTAGTAAGCATGTCGAGGCCGGAACAGTTTGGGTGAATAACTACCTGACAATGGCACATCAAGCACCTTTCGGAGGATTCAAGGACTCAGGAATTGGCCGTGAAAATGGTATCAAAGCAGTTGAAGCATATTTGGAAGAGAAGACTGTCATCATTTCTCTCCCTAAAAAGCTTTGAGGGTGTCACCAAAAATGTTTTAATCAATTAAATGCTCAATTCACAATAGCCAAATAATAATATTATCCTAACTTAAATGCACTGGATAATATTAAAATGACAAATAATTCATTTTTGCGTGGTTCCAATGAAGTTCCCTTTGGCGCGTGATAGTTTTTGCCTTTTAATATTACAATGTAAAAAGTACCTTATTGTTGTATGCGTATAAAAAAAAGTTTCAATTTTTAGATCATTTAAAATGTTAAAGTGTTCAAAAAATAATAAT |
| *Juvenile hormone acid methyltransferase* | GCAGGATGCAGCTGACACTTTAAATGAGTATTCGTCAAGATTAAACTGGAAAGAAACGGGAGAAAGTATATTAGACATAGGATGTGGCGATGGAAGTGTCACTGATATCCTGAGATCATACTTACCAACAAGTAGAAAGATTTTGTTGGGCTGCGATATCAACGAGAGAATGGTCAAACACGCCGAAGAGCGTTACGGAAATAAGACGACAGCAA |
| *Juvenile hormone epoxide hydrolase* | GGGCAGAAGAATACCCGCCCTCAGAGTCCGTATCACACCACAGCGGTGGGCACACGTGGCGTTTTGATAACGATTAATTATCAAAATTAAATGATAAAGCTGTCAGTGTGTACTTAATAGTGCATCAAGATGTCACGTCTTCTTTTTATCGCCTTGCCGCTGTTAGTGCTGGCGTCCATACCATTGTACCTCCTCGTTCTGAAGAGTCCACCGCCGATGCCTAAGCTCGACCTGGAGGAGTGGTGGGGACCGCCCGAACTTAAGCAGAAACAGGATACCAGCATCAAACCCTTCGAGATCACTTTCAGTGAAACGATGGTAAAAGAACTTAAAGAACGCATCAAGAAACGAAGACCTTTCGCCCCTCCATTGGAGGGTGTTGGCTTCAAATACGGGTTCAATTCGAAACAATTGGACAGTTGGCTCAAATATTGGGCAGAAGAATACCCATTCGCTGAAAGGCAGAAGTTCCTTAACCAATATCCTCATTTCAAGACTAATATCCAGGGTTTGAATATTCACTTCATGAGGATTACTCCAAAGGTCCCTAAAGACGTTGAAATAGTACCACTTCTGTTACTCCACGGATGGCCGGGCTCCGTCAGGGAGTTCTACGAAGCCATTCCTCATCTCACAGCTGTCAGCAAAGACAGGAACTTCGCTCTGGAAATTATCGCTCCAAGTTTACCTGGTTATGGCTTTTCCGACGCGGCGGTTCGTCCCGGCCTCGCTGCTGCTGAAGTTGCAGTCATTTTCAAAAACCTGATGGCCAGACTTGGTTACAAGCAGTACTACGTCCAAGGAGGTGATTGGGGAGCTCTCATCGGTAGCGCTATGGCTACTTTCTTCCCTAAGGAAATAATCGGCTTCCACTCCAACATGGCGCTTACTCTTAGTCCAGCGGCGACATTCCTTGAGTTTGTCGGTGCCTTATTCCCATCTCTGATAGTCGAACCAGAACTAGCTAACAGACTTTACCCGTTATCGGAGAAATACTCAACCCTTCTCGAGGAATTGGGCTACATGCATATTCAAGCCACGAAGCCTGATACTGTTGGAATTGGATTAACAGACTCCCCGGCCGGTCTCCTCGCCTATATCCTGGAGAAGTTTTCCACTTGGACCAACCCCGACTTCCGCAGTAAAGAAGACGGCGGTCTCTCCTACCGGTGGACAAAGGACCAGCTGATCGATAATCTCATGCTGTACTGGAGCACGAAGTCAATAGTCACGTCAATGAGACTTTATGCTGAGTCTTTCAGTTCTAGGCACTTCGATTTAAAACTAGATGAGATTCAAGTACAAGTCCCAACGTGGGTGCTCCAGGCGAAATACGAGTTAGCCTACCAGCCACCCTGCATCCTCAAACTGAAGTACCCGAAACTGGTCAATGCCAGTGTCATTGAAGACGGAGGACATTTCCTTTCGTTTGAACTACCGGAGATCTTCGCCAAGGACGTCCTGAAGGCAATCGGCGAGTTCAGAAAACTCAAGAACGTGAAGACTGAGTTGTGATTTGTTGTTTTTTAATAAATTGGTGTTTTGTTAACTTTACTTTTTAGATACTCTGTTATAACAAGCCGCGTTGTATTCATTATATACTGCTATTACCTAAGATGGCTAGACGAAGTCATGTGAATGGTTTTTTCCTGAATCATTACCAGTAACCATAATCAAATCAAATCAAATAAAAAAAAAAAATCAACATAAATGAAAGTACATACT |
| *Juvenile hormone esterase* | CGCGGACGACTTGACATACGTGCTGAAAGTGAATTCTGCGTCAGGGACTAGTTCATCACAAAAAGCAGACGATGAAATGAAATATTGGATGACGACGTTCGTCACAAACTTTATGCGATGCAGTGCTCCTATGTGCGATGAAACCACAGCGTGGCCACCAGTTACACCGCGGGAACTACAATACCAAGACATTATTACACCCAACTTATGCCACCAAACTAGTCTTACCAAAGAACAACTCGAAATGAAGAATTTCTTCGATAAGATCCATAACGGAGGTGAAAGCAGACTTAAGTAAACTCAAATTTTTAGGGTAGTATGGTGATAGATAGGTAGGTAGAATACTCAATGGTGGGCAGCGGTTTGGCTGTGACTCTGGCATTACTGACGTCCATGAGCAACAGTAACTACTCACCATCAGGTG |
| *Cytosolic juvenile hormone binding protein* | GATGTGCACTGTATAACCGCTGTATCAATAATTTTTATTGTGCCGCTGTACATTGTTGAAAATAGTTGTTTCGACTTCGTTATAAATTTATAATTATTCACCGTTTGAATAATCACACAAACTACATACACAGTTTATCATTACATAGATCACTCAATTCTGAACTAAAGTCATAAGCTCAAAGTTGTTAAATAGCAAAATGGTTAGCGGTCGTGCTCTTCATTTCGTATTTAAAGTCGCAGATAGGACTTTAACAGCAAAATTTTATAGGGAAATCCTCGGAATGAAGGTTCTCCGCCATGAAGAGTTTAGTGAAGGATGCGAGGCAGCTTGCAATGGACCTTATGCAAACCGATGGAGCAAAACCATGGTTGGATATGGCCCTGAAGATACACATTTTGTTGTTGAACTGACCTACAACTATGGTGTAACGCATTACGAACAAGGAAATGATTTCCTTGGCATCACAGTACAGTCAAGTGAGAGTCTGAAGAGGGCGCAGACTAACAACTGGCCAATTAAAGAACATAATGGTCTGAAGTATGTTGAGGCTCCTGGTGGTTACAAATTCTATATTGTAGACAAGCCCCAACCAGTTGATAAAGATCCCGTTGTCAAAGTTTCATTGGCGAGTTCAAACTTAGCAAAATCAATAGCCTATTGGAACGGTCTACTGACTTTGAAACTCTATGAAAAAACTGACAAAACTGCATTACTAGGCTATAGTGATGATCAAGCTAAATTGGAACTTGTTGATATTGGTGAACCAATAAACCGCGCAAAGGCATACGGGCGCATAGCATTCTCGTGTCCATTCGACGAGCAGCCGGTCATAGACAAGAAGATCCAAGACGCAAACGAGACTATCCTCACTCCCCTCATATCGTTGGATACGCCCGGCAAGGCTACCGTGAGGGTCATCATCTTAGCTGACCCCGACGGTCATGAGATATGCTTCGTTGACGATGAGAGCTTCAGACAACTGTCACAAGTAGACCCCTCCAGCGACGCCGAACTCGATAAGTTCATAAAATCAGACAAGTCCAGAGCATAAAATTTAATTATGCTTTAAAATATCGGGCATGGGTACTACTACTAACCTAGATATTAAGTTCGAACTCGTAATAACTCTTTGGAAATATATCCTCCGCGAGAATAGGAATGAGTTATATTAATTTTATCGAATCACTTTTTATGAATAGTATTTATTAATAAACAGTTGTCGTGTTGTCTAATTTTAACCGTCGCTTGAATAGTGAATAGCTTCAGGTCACAAATTTAATCATTACAAATTCTTAAGTAGTAAGTAATAACCGTAGTAATATTTATAGGTTTTTCCTGTGTACAAGCGGGAAAAGTTTCGGTTTTTAGAAAATATTTTGTCGATGTTATTGTAGCTTGTTTGGAAACGATAATCATCGAATAGTATCTTTTGAATTTAGCAATAAACTTATAATTTCGAATGTTTTTTTCGAAATTAACACGATCGATGTTAAATTTGGACAATCCACGATTGCCTAATATTATATTTATTACATATGTACGCATTGTGATAGTCGTTTTTTTTTTCCTTCGAAAGAAATAAATTTGTATTTTAATATTAGGTACTAAGATTCAAATACATGTTAATAAATTATTTATTCGATTTCGGCGGTAATAGACTTTCTAATTGAAAGTCAAAATAAAGTACGTACCAATTTTTAAATTAATTAAAAAAAAAAATATTTTTACTAAATCTTGCTACATTCCAAATGTTTTAAATGTTTGCTGTGCTTTAAAATATTTATGTTTGTTAAATCATTAGATTATTATAAAAAAAAGTTGCTCACGTTAATTTGATATTTATATAAATAAATTTTCTTATATCTTCAA |
| *Juvenile hormone binding protein* | TGTAAGTTTACGATTATGTTGACGTTTCTCTACTTGTTCGCTCTCGTCTTAGGGGTGAAGAGTGCCTCGGCACCCTTCATAACACCGTGTAAAGTTGGAGATTCTGCCTGTTTTGTGGCTTCTGCACAAGCGGCAGTGCCGATCGTGGCCGCCGGCATTCCCGACCTGGGCATCAAGCCCTTGGACCCGTTACACATATCCGTGATCAATGGGAACCAGGGTGGGCTCGAGTTGACCTTTAAGGACACCATCGTTCGCGGCTTAAGCGGTTGTCACGTGGAAGGTGTTAAAAACGATCCAGCCAAAAAGAAGCAAGCGGTCACCATCAAATGCTCCGTGACGCTAACTGGTGATTACAAACTTAGCGGTAAACTGTTGGTGCTTCCCATTGAAGGCGAAGGAAAATACAATATCAAAATACGAGACATCGTGATCAAGACTGCAAACGATCTGGTGACAGTGACAGGAGCTGACGGCAAGCCACACTGGCATATCGAGTCGTGGAAGCACACGTACGAAGTGAAGACTGGAGCTCATTTCCAATTTGAGAATTTGTTTAATGGAAACAAGGTGTTGGCAACTCCCGTCGAAGAATTTGTGAACTCAAACTGGAAGGACGTGATGCAGGAAGTAGCCCCGCCCATCGTCCGGTCCATCGTGTCCGAGGTGGTGGCTGCTGTAGATGCCCTGTACAAGGCTGTGCCGGCCGAAGAACTTTACTTGCAGTAAACTTATTGTAATTTTAAAAGGGAAAGCTGTATATGTGATAAATAAAATTACTTTTTTTTGGTTACACTTTTTTGCTACCTGTTCTTTATTCTG |
| *Juvenile hormone esterase binding protein* | GAAATTACTACTACTTGAAAACAAAACATTCCAACCTATGAAATAATAATAATAAAAGTAATTTTTATTCTTCGAAAACAAATATTTATTTACATGTATCTAACGTAAATATAGGATCATCTGAACAAATCGTATGTGCTTGAAATTAGTTAAACTCTAAATATGTCATTACTTCAAAAGCTAGCGTATAACGACTATAAAAAGGCAAAAGATTTTACAGTACATCAATGTGAAACAATCGCTAACTTAGCGAAACTGAACACAACGTTTACAAATGTAGAGTGCCCAGGAGAGCATTTACTGAGAAAATTAGATCTAAGTGGCTGTTCTGTGTCACAATACAAAGTATTGCTCAGTGCCTTGAGGAAATATCGACCGCATTCGAAGATAGCTGTTTTAATTGCAAATAACTTATATACACATCTATCAAAACTCGCCACTCCCTCTGTAGACTGTGACTCTTTATCCCTGCATTTGAAAAGTTTGGGATTTATTGTTGTGACCGTAAAGAATACACACGCAAGCCATCTAAAGGATATTGTTGGAAGAATATCTCATGTCATACCGCCTGATTCGTATTGTTTTATATTTTATGCTGGTCATGGCTGCCAAATAGGTAACACAAAGTGCATGCTAGGCATTGATTGTCCCACAGAAAATATACAGAATGAACATTGTATGACCGAAAATAACATGCTGAAACAATTTGAAAGATGTAATTTAGATTTATGTGTATTTATTTTGGACATGTGTAGAATCAATCTTGACAGAGAAACAAATCCTGATATATCAAATTCAATTGTTTTTACTGAAGATTACATTGTCCACAAAAATCTACTGATAAGTTATTCAACGCAATCATCGCAATCAGCTTATGAAGTGCTCGAAATTGAGTGCTCAACTACTATTAATTTTGATGAAACGTACGAGCTAAAGACTGGTGACACAGACCGTATTGTGCCTGGAGCCAGTCAGTATGTGAAAGCTTTGTGCAATAGGATTGGAGAGGATTGTGATGTCAGTAGTCTTCTAGATAGAGTGCATGGTGATGTCGAAAATTCACTTCGGAAACAAAAACCAATAAAAGTACAATGCGGTGTTGAAAAACGCCAACTGAACGACGTTCCGAATTGTGAACCGATTACATTAATCAACAAATTGAAGGAGTCGATAAAAGATTATACAAACTACTGTGATGTTTTCTGAATATTCAAATAAAGAACAGATTATTGTGACAAGAATATAATTCTTATTTAAATACAACTTTTTTTATAACAACAATTATTTTTGTATTTATTTTTAAAAATCCAGTTATCAGTCTATAATACAATTTTTACATTTTCTTAATAAGCATATGAGGCTGAAAATGATTAACAATGTTAACCCACCAACTGTCTTCAACTCCTTTAGTGAACTCTCTGATAAATCTGTAGTTCAAAATGAATATGTTATCCTGGTACTGGGGTTGAGCTCCTAAAGTTATTGGAGGCATGTCACCGCTTTCCTTCATATTTTTCAGCCCTCTTAACACATGATAACACATTGTGATTTCAACCCAACGCTTATCCGAATCATCAAAAATTACTCCAACTTGATAAGGAAATGCATAAAATATATCTTCTTTTTCTATGGCTAACAATTGCCGCTGGGACACAGAAAGTTGAGACACAGCAGTCTTCAGTGCATTAATAGCATCTTTATCCACCAAACCCTCTAGAGTTTTGAAATCACTGTTTTGTAGTGCTCCTGAAACAATCTGAACTGCATGTTTGGAAGCTTCAATGAACTCGTTCAGACTAAATTCTTGATCAAAATACGGTCTTATAATAAAATTTGAAAACATCCAATTCTTTACGTATTTTATGAATGAAGGCCACATTATCGGCGGGAATTCCATCAATTGAGGCAACTTCCGTGTTTCCGTTCCTTGTTCGGAATATTTTCGATATTGTATAATAGGACATTTTGAGGTTATTGCTATTTGTTTTTGTACATTATTATAAGCGATTTTATCGCATAGCCGTAACGATTGCCTCGTTAAAACATGCCTTAATACTAAATTCATATTTCGCAAATAATTATCAAAATATTATTCAATCTAAATAAAAAAGGTATTCTTTAAACCAAAACAATCTCACAACATTCGAATTAGGG |
| *Hexamerin* | CGGCCATTTTCTTGATATGTAACCAGTTTCAGCAGACAAAATTTTGTTCAGCGGTATACGATAGTATTCAAAAAAAGTTTCCATTTTAGTAAAATTGGCACCAGTAATATTAAAATCTTCTGTTGTATACTCTTCCATTAAGAACGGATCCATGAAATGTCGAAAATACTTCATTACGTTCAAATAATACTCTATCATCTTCCAAAAGGTAGGGTCTCTTAGTGATGTTTCAGGG |
| *Broad* | CTGGAGTAACTCAACAGAAAGAAAACTTTACATCACCTAATACATCAAACAAACTATACACTCAGTTAACAATTTCATCAAGACCACACCACAATGCCTCCCACAAGGATGCAAAGTTACCAGGACTAAAGAAACGCAGAAGCAGCACTTCAGACAAGCCAAATGATGTGTCAGTCGGTGACAGTTACAAAAAAACCAAGGTCCTGGATACATGTGATATTCCTAGATTAAACAATTTTTCACAATCTTCCAAAATTGACAATGTGCTTCCTGTTGAAAACAATGTTGACAAGAAATGTGATGGAAAAAATGACTCTCCACTAATAACTGCCAATGGGAAAAGTACATCATCTCAGGTGCATGATTGTTCAATACCAATGAAAACCGAAGTGCTTGATGATAATTCAACTGAATTATCAACTGCCATTCTATCAGAACAAGATGACAGCCTTCCAGATTATGAAAACAGCATGCTTGCAAGGTCCTTGTTATCAGGAATTAATCCTTCAAAAAGTGACAGTGCCAGTAATTTATCAGGAAAAAAACCAGTTAATATTGGAGAAATAGGTCCCAGTAGATCAAAAGATTCAAACACTGAAACTGTTTCACACAATAATTCATCTAGGAGTCCAATAAAAAACACATCTGATGAAGTACAAGTAAAACCAGAAAGACAGTCACCAAAATCTGATGTGGAGTATGAACCAGAAGTACTTCTCTCTGAACATCAAGATGGTGATACAGAGAACACATATAATCAAGAACAATCGCAGGCTTTATTACTACTTGCTGGCATGTCCACTGTACCAGGTTTGGGAGGTGGAGCTTCAACATCGCAAATAATGTCCCACCAACAATCTAACCATGCTGCTATTTGTGGGGATTGCCCGCATTGTGGAATGAAATATTCTAATCAGTCAGCACTCAAATATCATGTAAGGTTAATGCATTCAGATTTAACGAATAGATTGTGTTGCTATTTGTGTCCAAGATCGTTCACAATGCGTGAAACTTTTAAGGAGCACATGTGGACAAGTCATGGTCAAAGAAACTAATTATTTAAATTATTATGGTCTTATATTACGTGCCTTAGCCTCAGTCCCTTGTCAATTAGGACAAGCCACTATATAAGTAAAATATTTTTAAAATGTGCCTTGTTAATAAAGTATTGGCAATGAAATCATAGTGATTGTTGTCAATTTGTGTCAATCATCAAGTGTTTTCGGCCATCTAATGTTAACTAGAAAGAAAACACCTTTAAAATGAGGAACGATATTTATAGGTAAATTGCTGAGTACAGGAATGTTCGAGTATTTTTATATACCACAATGGACCCTTAGGAATTAAAATTATTTATAACATATACTTTTCAGTGATTCTGAAAGACAATTTATTTGACATGAAAATGTGTATGAGTGAGTTAATTTACTTATGTTATATATGCTGGAGATGGTTCCTTACGAGTATGGTTTTAATTAAAAAAATAAGCAAGAGGAAATTTGTTGGGTCTCTAGTATGTCCGTGTCGAAATTCAAACATAGTTCTTATAAAGATAAAATTGATCCGTCTAATACAATTTTGTACTAAAGTGAATTAAATATTTTAATTGAATTGATGTAAGTAGAGACTGACGTTAAACTCTGCAGTGTTTTAATGTAAGAAAAGATCAGTTGCTTTTTTTCTATTTGATATAAAATTATTTTTACACTGTAAGTAAATTTGTTTTTGAGATTTTGAGAAAAAAATATTTGGTGCAAAACTAATGTGATTGGTGCTAATATATTTGGTGTTCTAAAATTTCACAGTTAATTTTATAGATTATTAGAATCCTTTTAGTTATAATTATGAGCAAGCTCATATTTTATAGTTTTTAAATGGTTATTTTTTTCTCAATAATGTACGTGTAATGAATAGTTATGTAGTGAGTCGTCTATTATCAAAGGT |
| *Allatostatin receptor* | TTCTTCAGCCGTCTGCAACAATCTTTCATCAGCATCCAGTCATCTTGAACATTTTACTATTTCGTTTATGTGGAAAATGCCTTCAATTAACTCCGAAATTTCAAAACGTTTTCTTATCTCGACCGCCATTTTTGTGGTTGAAGCGTTGTAGTAGTTTCGTACTTAATTTCATGAAATGAAATTGAAAAGCATAATTTGAAGAATTCATTTCAGTCGTGCTTGCTGAAGCTCAAATACATTAAGTGTTTAATCTATGCTGAAGCTGTTCCTAAAAATGTAG |
| **circadian clock related genes** | |
| *Cryptochrome 1* | GTCCGCATTCGTCTTGATTGCCGGTAACTTACTATGATTTTGTAATTTTCATTCTGAAACAATTACAGTATACTGAATTTTCTTTACGCCAGTGCATCGGAGCAGCGTTCTTGCCTGATTTCTAAACGAATAATTGTAAGGGGATACTAAAGATGCTTGGCGGTAGCGTTCTGTGGTTCCGTCACGGGCTGCGTCTCCACGACAATCCGTCTCTTCATTCGGCGTTGGAAGAAACGAGCGGCCCTTTCTTTCCCATTTTCATTTTTGACGGGGAAACTGCTGGAACGAAAGTGGTCGGGTACAACCGAATGCGGTACTTGCTAGAAGCGTTAGATGATTTGGACAAGCAATTCAAGAAGTACGGCGGAAGATTACTCCTTGTTAAAGGGAAACCCAGTGCTGTGTTCCGCAGACTCTGGGAAGAATTTGGTATTCGGAAGTTGTGCTTCGAACAGGACTGCGAGCCGGTGTGGCGTCCCAGGGACGAGAGCGTGAAGACGGCGTGCCGCGAGATCGGGGTCACGTGTCGGGAGCACGTGTCGCACACCCTGTGGGAGCCCGACACCGTCATCAAAGCGAACGGGGGAATACCGCCGCTCACGTACCAAATGTTTCTGCATACTGTGGCGACCATCGGTGACCCGCCGCGGCCTGTCGACAACGCCAAACTCCGCGGTATTAAGTTCGGTACATTGCCGCTGTGTTTCTATGAAGAATTCACTGTTTACGACAAGGTGCCGAATCCCGAAGACCTCGGAGTGTTTCTCGAAAACGAAGACATCCGGATGATACGTTGGGTCGGTGGCGAGACGGCCGCCTTGAAGCAGATGCAACATCGACTCGCCGTCGAATACGAGACGTTCTGCAGAGGTTCCTATCTACCGACGCACGGCAGCCCCGACCTCCTCGGGCCTCCGATATCGCTGAGTCCCGCTCTACGGTTCGGATGCCTCTCCGTTAGGAAATTTTACTGGTCGCTCCAGGATTTGTTCCAGCAAGTGCACCAGGGAAGCTTGTGTTCTACTCAATACATTACCGGTCAGTTGATATGGCGCGAGTACTTCTACACGATGAGTGTGAACAATCCTCATTACGGACAGATGACTGACAACCCTATTTGCTTGGACATCCCCTGGAAAAGCCCTGAGGGAGATGAACTGGAGAGATGGGCGTCGGGGCGCACGGGGTTCCCGTTCGTGGACGCGGCCATGCGGCAGCTCCGGCTGGAGGGCTGGCTGCACCACGCCGTGCGGAACACCGTCGCCTCCTTCCTCACGCGCGGCACTCTGTGGCTCTCCTGGGAGCACGGGCTCGCGCACTTCCTCAAGTACCTGCTCGACGCCGACTGGTCCGTGTGCGCCGGCAACTGGATGTGGGTGTCCAGTAGCGCCTTCGAAGCGTTGCTGGACTCGGGCGAGTGCGCGTGTCCAGTGCGACTGGGCCAGCGGCTCGACCCCAGCGGCGAGTACGTGCGGCGCTACGTGCCCGAGCTGGCGCGGGTGCCGACCGAATACATCTACGAGCCCTGGAAAGCGCCTCTGGACGTGCAAGAACGTGCCAACTGTATAATAGGCAAGGACTATCCCGCGCCTGTGGTCAATCATATCGTAGCTGCGCAGCGCAACAGAAACGCCATGGAGGAATTACGCATGCTTCTGGAAAAAGCGCCACCGCATTGCTGTCCGTCGTCAGAGGATGAAATCAGGCAATTCATGTGGCTTAACGAATAGAAGAAAAAACGACTCGCTACAGTATATTTTTATTTGTCTAATGTACCGTATGACATTCTTTTTCGCAGCTTGGCTTTTGCGTAAACCGGTTTAAGCGAATCCGGATAAATCCGCTGGTTCCGTAGATCATGCGAAAAGCGGGCCTTGCATGCGAGCTTTGGTAGAGTCTACCCGTGATCATGGCGTTTGCGATAGCGCCGAAGTATCGGGGACTCCTTAGTTAAAAATCCCGGTCGGAACATGCTATATGATAGTGCTTATCATTATATAATATGTGAAATGTTCATTTTCCGGCTCAGTGAGTTCGGTGTAGTGGGTTAATTTGTTAGAACATACATTGAGCCTGGATACACCTGATCCCCAGTCGGGGACAGTTAGGGAACTTGCCCAGCAAGATATCCTAGGCCCCTGATATGCGTCTAGAATTTAAGAAATTAGCATATGGACGCACCTGGAACTGCCACGCGGAGTTCCAAGACGCGTGCACTGTTTTCTGGAACCACTTGGATTCGATTGGTTTCCGTTTTTTGACATTTGTATAACTTGTCACCCGTGACAAGCCAGTTTGGTTACGTTCGAAAAAGGGGTACTTAGTTGGTTGCGTGTTGTTCGACTAACGTTTGTAATCGATGTATGTATTTATATTCATGCGATTCTGGTTCTCTTTGTCATTCCATGAATCCAAATGTGGAGACCGATTGGCAGGTCTAATGTCAAGTTTAAAATTTATCGCGAAGATAGTACAGTAATAGTATTAACGTACTTAAATAATTGCATGTCAGCGCTATGAGTATAGCTATTAGGTGTGGGACTTTTTTATTTGCTATTTTTTGGGTCTTAAGGTGGCCATGGCTTTATGATCTCTCCATTTTGTGTTAAGTTTTGTATAATAAATATCGTACCAATATTAACTAACTATTGTTGTATTTAAATATTTAGGAATTACATCGTTGCTATCAAAGTAACTTTCACTTACTTGTTGTCAGGTTGATGTTTTCGTACGATACAAAAAATTCTAATTAAAATTTACTATTAACAAGTTCACGGCTATATATTGCCTCAGTCGGACGCTTGAGCTATTACAACGATAAGCATTTTGGCTTAAGTTTGCAAAAAACATGTTAACGGTTTGCAATTTGTTTATAATATTTGAGTCGACTATTATCA |
| *Cryptochrome 2* | CAAATACACAGTACCGCAGTCTTGTTCGTGCTTAACTAGAGACTGTGTCGTCGTAATTTATTCTAAATGTTCTAGAAGGTGCAGTTTTCACATTATGCTCAATTACTAAATTTTTTAAACTTAATTGAAATTATTTAATATTTAATTTTCAATCAAGATTCGGATGTGTACGTGCATAGAACACAACTTTATTTACATAAACAAAAGTGTATTGCTAATATTATATTTTTAAGCTTTTACTGGCTATGAACTTAGATCTTCAGTGTTATTCGAGTTAGGATGTAAATAGGAGCTTATAGAAACAAAAATGTCAGCAGCCCCAGAAACACTGCCACCACCAAGTGCTCAAGCTCACACGCCGGCCAGGCCTACTCATATGTCCGCACCTCGACGTACGCCGGGTAAACACACCGTGCATTGGTTCCGGAAAGGATTGCGAATCCACGATAACCCCGCCCTTCGAGAGGGCATTATAGACGCTGTCACATTCCGATGCGTCTTTATAATAGATCCATGGTTCGCCAGCTCTTCAAACGTTGGCATAAACAAATGGAGATTCCTATTGCAATGTTTAGAGGATTTAGATAAAAGTTTAAAGAAGCTTAATTCGAGGCTATTCGTTGTTCGCGGTCAGCCCGCGGATGCGTTGCCAAAATTGTTTCGAGAATGGGGTACAACAGCATTAACCTTTGAAGAGGACCCTGAGCCTTACGGCCGCGTACGCGATCACAATATTATATCTAAATGTCGTGAAGTTGGCATAACAGTGACCTCTCGCGTCTCACACACTTTATACAAATTGGACAAAATAATAGAACGCAATGGTGGTAAAGCTCCGTTAACATATCATCAGTTCCAAGCGTTAATCGCCAGTATGCCGCCCCCTCCACCGGCCGAAGTTACAATAACTCCGCAAATGCTCAATGGAGCCACTACACCGATTACTGATAATCACGATGATCGTTTTGGAGTCCCCACATTAGAAGAGCTTGGATTCGAAACCGAGGGACTAAAGCCACCAATATGGATAGGCGGAGAAAGTGAAGCTTTAGCCAGGTTAGAGAGGCATTTAGAGAGAAAAGCCTGGGTGGCTTCATTTGGAAGACCCAAAATGACACCGCAATCATTGCTGGCGAGCCAAACGGGACTATCACCATACTTAAGATTCGGATGTCTGTCGACCCGATTGTTTTACTATCAATTGACGGAATTGTATAAAAGAGTGAAACGGGTGCGGCCTCCGCTCTCTCTTCACGGACAAATATTATGGAGGGAGTTCTTTTACTGTGCTGCGACAAGAAACCCAAATTTCGATCGCATGGAAGGAAATCCTATATGCGTTCAAATACCTTGGGAGAAGAATCAGGACGCTCTAGCGAAATGGGCAAATGGGCAAACCGGATACCCTTGGATTGATGCCATAATGATTCAATTACGAGAAGAGGGCTGGATTCACCATTTATCGCGACACGCTGTAGCCTGCTTTTTGACAAGAGGAGATCTTTGGATTTCATGGGAAGAAGGAATGAAGGTGTTCGATGAGTTACTCCTCGATGCAGACTGGTCGGTGAACGCAGGCATGTGGATGTGGTTCTCCTGCTCGTCGTTTTTCCAACAATTTTTCCACTGTTACTGCCCTGTGCGTTTTGGAAGGAAAACCGACCCTAATGGTGATTTCATAAGGAAATACATACCGGCTCTGAAGAACATGCCAACGCGCTACATACACGAGCCGTGGATGGCCCCGGAGTCTGTGCAGGCGGCCGCCCAGTGCAGCATCGGACGTGACTACCCCATGCCGATGGTCGACCACACCAAGGCCTCGCAGATAAATATCGAACGGATCAAACAGGTCTACGCACAACTCGCCCGATATAAGCCTCAAGCTACGCTAAATGCTAATGCAGTCCAGCGACCCAACGTGTTGCAGTCGTCGCCAAGTCCGACATCAATAATCGCCAGCATCAACCAGTCCAACTTCTTGTGCAGCCAAACTCCGGATCCACAAAACGCTACATCGCATTCGTTCAAAGAGCCGTCAGACACATTCCCAATAAACAAAAAAAACAAAGATACCGAATCGAACCAAACTCGCTACAAACGAGTAATTATCGTGCAAAAAACACGCAATTCTAATGTGGAAATTAATGTATCCGCTCCACAAGATCGGAGAGCTTCAGATAATTATATCACGATTAATGAGGGAGATCCTGCACACAGTACAGACGCAAAAAAAACAAGAAAATAATTACGATTTCAAAAATTTGGCTATCGACAATCATATCCGAGGGTTTAATAACATTCAAAATATAAATTCTCAATATTATTTAACTAATTATTCTACTAAGAAGAAAGAAACTGAAATAAAAAATGAAACAGACGAAGGTCCATTTGTGCGCCCGGAAAGTTATGGCACAGACAGGGCTTACACAATCAACCGTTGCCCTTCCATGTCAAATGAAAAAGAATTTGAAACGCCCAAAGAAAATCTAAAACAATAAAACTTGGAAAAAAAAAATCGTTTTGGAATAAATAAAAAATATTATACAAAATAAATAAAGATATACACAAAACAAACAATACATAAAGTTTTTTTTTAAATTAAAAACGTTCACTGCGACTAGGTGAGATACGTGAAACATTTATTACATACATTTTTACATTTCAAATACCTGGCATCTCAAAGAGTAGAATCCTTAAAAGCATTTTTGATAAAATTCTTTTACCACCTGTGTTTTTTATTCAGATGCTTCTCGTAGGGGTATAGAGCCTTCAAAAGTTTATCAGTTATAGGGCCGATAATCGCCTAAATAAATGAATTCATGACATTCGGAAAAACACACAGGAAAAGTTATACCTTTGGGTTTTTGTTATTAGATCACGGAACCCAGAAAAGCTCTGAAATCTGTATCTATCACTACAACTTTGAATATTTTAGGTTGATTTTTTTGGTGTGATTTATTTAAAATATTTTGATAGTTTTTTTTTAATGATTTCGTTCTGAACATACTTAGTGATAGGAAACGTAAATTAGATGTTTTTAAAATGTACATTTAAATATTATGTAAGAACTAGTAGTACTATGAACTAGTTTATGTATACAAGTTTATATTTCAATTTGTAGTGAATAACACAAAATTATAATAATATTGTTAAATTTCTGGACCGGCAATAGCAACATAGAGCAGAGCGTCCGAAGATGTTGCATCATCACCAATATTTAACTGCTTTAAGAACAGGCTCGAACGATTCTCCAAATGTCTGTCCGCCTTATTAAGCTTATTAAGCCACAAATAATCATCTCTATGTTCGAGAAAGAGCAAGCAAACCATTAAACAAATTGGTGTTTTTTAAGCATGCGGTAGGCAGCGGCTTGGGTCTGCCACTGACATTGCTGAAGTCCATGGGC |
| *Period* | TTACAGTCAATCAAGAAGAAGTCACAGCTCAAAATCTACTCATTCCGGGAGTTATTCTAGCGGCAGTAGCGGATACGGAGGTCGACATGTTACTGGCACTAACAGTAATAAGTTGGCTCAGTCACCAAATAAAGATAAAGAGTTAAAGAAAAAGAAACAATCACAAACAGAGCTAACGACTACCAAAGAACAAAATGAAGGAGAAAGCGGACCTCAAGAATCTGTGAACCTGGAAACTCTAGAGGAGGAGAAGCATGATGTTACGAGCGCACCCTCTCCAGCGCATGTACAGGTCGAGAAGGGACCTGAGAATATGGATATAGCTTCAACAGACACTGAAGGCGACAAGGTAGAGGTTTTATCGTGTGACCTGCAACCGCAGAACTCTACTGTCGATGTAGTTACGAGTCGTTCGAATCTAATCTGTTACACTGATGGATTTTCATGCGTTATATCGATGCACGATGGAATCGTAATGTACACGACATCATCGCTGACGGCTACACTCGGTTTCCCGAAGGACATGTGGATCGGGAGGTCATTTATCGATTTCGTTCATCCAAGGGACCGTAGCACGTTCGCGTCACAGATAACCAGTGGTTTGGCCGTCCCGAAAACTGCAAACGCTCAATCGCCGGGTAATTCTGGATCGACTATGGTGTGTCGGATAAGACGTTACAGAGGTCTGAGCACGGGGTTCGGAGTTAAGGAAAGAGTGGTCACGTTCATGCCGTTCCTTTTGAAATTCACGTTCAAGAACATAAGCGATGAAGAGGGAAATGTCATCTACCTCGTCATACAGGCCACACCATTTTTTTCAGCGTTCAAAACGTCGTTTGAAATATTACCGAAAGTTAATCCTTTCGTGATGCGTCACTCCGCGAATGGTAATCTCGAGTACTTAGACCCAGAATCTGTACCTTATCTCGGATATTTACCTCAAGATGTCCAAGACAAGGACGCATTGCAACTCTATCATCCGGAAGATTTGGAATACCTCCAGCAAGTATACGAGGTAATCGTTAAGGATGGTGGCATGCCGCGTTCCAAAACTTACAGAATGATGACACAAAACGGTGATTACATAAAAATCGAAACCGAATGGTCTTCGTTTATTAATCCTTGGTCGAAGAAGCTGGAATTCGTCATCGGGAAACATTACATCATTGAAGGCCCCGAGAATCCAGATGTATTCCAGTCCCAAGATCCGGAGAAACATACAAAATTATGCGACGAACAAATAAAAAAGTCCATGGTATTTCGCGAGAATATCGTTAAACTTATGAATGAGGCTCTAACGAAACCAGCGGAAGTTGCCAAACAGCAAATGAGCAAGCGTTGCCAGGACTTAGCGTCGTTCATGGAGAGCCTCATGGAGGAACCACCGAAAAATGACGAGGAATTACGCCTAGAAATACAGGATCCGGACCACAGTTATTATGAGCGTGATTCGGTAATGCTCGGCGGGATATCTCCCCATCACGACTACAATGACAGCAAATCAAGCACAGAAACACCGCTGAGTTATAATCAACTAAACTATAACGAGACATTACAAAGATATTTCGACAGTCATGAACCCTTTTCGAGCGAGAACTTCAATCCCTTGATTGTGGCAAAGGAGCCAAATAATTTAAAATCGATACTAAGTACTTGTTTATCACCAATGGCTCAGAACTCTGGCGATTCGACCGA |
| *Timeless* | ACTCGCTCTATGCCGCGGAGTGCGGAGCCACGCCGCTCGAACAAGGTTATCTCTCTCGGATTACTTCGTAGTTCAGATTCAGTCGTCGTCATTCGTTCGATTGCCGCGCGATACGTAGCACGTGCCCGTCACAACGTGATAAACGCGATAAACTAACGCACTATTTACGAAGAAACACTCGGTTGGTGAATTAGAAATTCAAAAAAAATAAAAACAAAAAAAAGCGTTCTGTGATTTCAGTGTACTATTTTTATTGCGCTGTGCTATGTGAATTATGACAAGTGTTTTGAAAAAGCCAAGAGGCACAAAAGGCAAATCGGAATCCATGCAAGAAGACTTACCCACAAGATGGGGGTCGATCTCCCGTGAGGAAAAATACGAAGACAAGGAGAAGTTGATCAGAGCCATCGACGACAATGTTATTGGCAAAGGCAATTCGTTTTGTGGGCCCTACGGAAGGAGGAAAGTCGTGTACTGCGACTTCAGCGGGTCTGGACGCGGATTACAGTTCATCGAGGATTACATCGTTAGAGACGTTCTGCCGACACATGCTACTAGGTGTACAGCGCTCTCGTACACTTCCGGCCAAAGCGACATTTACAGATCTGAATCGAAGAGTATAATACGCAGCGCCGTAGGAGCTTCTCCTGAAGATGTTTTGGTCCTCGGCGCCTCTTTACCCATGTTCCTACGAGCCTTGCGGCCGCAGAAGGTGGCTTTGTTCGTGTCTTCACGGGAAACGGAGACCCAACTGGCGCCATGGAGGGAATTCAATTCAGAGATAATTAAGATAGCAGAAACAAAAGAAGGTTTTATCGATCTCAACAATTTGGAACAAAAACTGCAAGAGAACTCTGGCACGGGAAAACGAATGATTGGATTCTTTCCTGCCGTATCCAAATTGACCGGAGTCCTCGCTGATGATGTTGCCACTACCTTGCTGCTCCATCAATACGGAGCCTGGTCTTTCTGGGACTACACATTGGTTGCTCCCTCCTCGACCGTGGAGATGAACCCGGTAATTCCGGGAGTGGAGGATGAGATGGTCAAAAAGGATGCGGTGTTCTTTAACTGCGAGAAATTCGTTGGGGGCGTTCAGGGACCCCATGTCGTCATCTTCAAGAAGGAAGTGTTTAAGAACAGTCTTGTTTATTCTGACGACGTTGAGGTATTGAGCGAGAGAATAGCAGAGATAAGATCGACAGACGCAGTGCGGGCTGTCATTGTTATGCAGTTGAGAGACTCGGTAGGACTGCAGAACATAGTGCAGCGACAGGATTCCATAACCAGACAAGTCCTGTCACACATAAAGAACATCCCAGAGCTAATTCTTCTAGGAAACGTCTCACCAACTGTCAGAAGGCTTCCGATCTTTTCTATCATGGTGAAGCATCCCCGGGGGACTTTCTTGCACCACAACTTTGTTTGCGCGGTTCTGAACGACGTTTTCGGCATCCAGGCCAGAGGGGGCCTCAATAGTAACTTGAATTACGGCTCCGATGTCCTGGGTATAGATGACCAATTGCTCAAGGAGTATGAGAAGCTGCTTGATGTCGAAGCACAAAAGGAGATTGCTCGTGTCCGAAAACTCAGTAATGTGACGTCTCCAGAGGTTCCTATACACAACGAGCACTTGAGGCCTGGATTCTGCAGGGTTTCGCTACCGTACTTCATGTCGGAAACCGAATTGGCATTTGTTCTTGAAGCACTGAAGATGGTGGCCACTGAAGGTTGGAAGATTCTGCCGCAATACGTGGTGAACCCACAGACCGGACAATGGAAGCACCACACGTGTTCGGTTCTTAGAGACAAGAAATCTTTGTATTCAATGAACTTCAACACCGGTAAAATTTCGGTCAACGAAAGACGGATATCAGGTCCTGGAATATTCCCGCAAACGTATACCGAGTGCTTACAAACTGCTCGGAATTTGTTCAACAAAGCTAGAAAACTGGCAATGAAATTTTCGTCAGCCGATCCGGAGATGACATTCAATCCTCGCATAGATTACCTACGTTGGTTTATGTTGCCCAAAGAGGCTCACGATCTGCTACTCGGGCGCTCGTCGAATGTAAAACACATAGTGCCATTTGATCCTTCCGGGTACACGGGAGCAAGAAAAAGCCTTCACGTTTCGAGATCTTCAATAACTTCCTCACCGATATTAGGATCGTCGGCAAGACATTTCAGTTTGTCTGCCATTGATGACTGCCATGTATTTAGACTGAAACAGAAGACCAAGTTTTATTCAAGAGAAACCAGCCTCCGGGAGGCACAGAAAGAGGAGGACACAGTTTCACGGCCCGTGCAGTTCACAGTTGGTGAATCCGTTTCACCGTTGCGCTTAGGACCTTCAAGGCAGCCTCTGCTGGTCCGATCCCGATGCTACAGCTTGGGCTCTGATAGCCCTCCGGTTCCGTTAAGTGCACAAACAAAAATGAATTTAGGATTGAAAGAAGCGAGTCCTAATAACGTTATTGAAAAGACTAGTAACTTCTGTAACTGCGGCAGTCAAACGGACCTATTTTCGTTGGACGATCCGCTGATCTCACCCGGAAAGTCATATCCGTACAGCACGCAAAGTAGCACGTCCATATCGGACTGTAGTCAAGTCGGTCGCAGCTCACCCACCACTTCTCTGACGTCACACACGTCGGAGGATCTCCAAGCGTATGTCAAAGAGATGACCCGAGAAATCGCAACGGAAATCAAGTCGGAAATACGTGAAGTAATCTCAAAGGTTGACGATATTCTCGAAAACTCCGATGCAGTAGAACAGTCTAATGCCAGCATAACTTCAATATCTAGTCAGAACGAAAAGGACTCTGTATCAGTCATAGATGTAACTGAGTATTTAATGGAGATGTCAAAGGAAATGGCTTCAGAAGTCAAACACGAGATAAGAGAGATGGTCAATCAAGTCGACGGAATGATATCCCCTGATTATTCAGGTAACTGTGCAAGAAGAGGCTCTCCACCTCAGACTGGTCGTAGAAGGGTAGGCTCAGGTCCAGAATTAGGTCTCGATTTAAAGAAATCTACTCAGTTCCTAAGGAAATGTCCCACCAGTCCAGTTCTGCCGTCGCAGTACGACGAGGAAGACAGGTTCTGCAAGCGATCACCTCAGTCGCCTAAATCGGCTCAGTGTACTCCTTCGCACGAGGCTTCAGGACCGAATAGCATTTCGTTTAATTCCAGCGAAACCTCTACTCCCGACACCATAATCCAAGTGGTCACGACGCAAAACTCGCCAAATCTCCCAAAATCATTAAGCTCGAATAAGTTGTCTGATGATGAGGTTTGCAACGACGCGACCTGCCGTCACTGCTGTATCAAAAAAACTTGGTGCCAAAACCCGTCGGTCAGTTCCCAAGATAGCGGCATCAACATGACCTTCACGGACACCGATACTTATTTAGACTTTGATCCGGCCACGAAACAGGCGAGGAAGATCCAGGGAAGACTTCGCATGTGCCCGAAGTATGAGAAGAGTGAAGTGCCGGATATAATTGAGGGAGTTCCGGTGTGTCCAGGGGAACACTCAAGACAGACTGCCAAATATGAGACCACTGCTGACGGCAAAATCGTCTTTCAAATACCTGATGACGCGAATAGGAAGAATAGGTTGTCTACCATTTGTTTTCGCCGCTCCCGCTTGCCGCGCCTCACGACGCACCGCAATTCGCACCGCTCTCGGCTGTGGCCAAGGGGAACAAAATGGAATGGGTTTTAAGAAGTCCACAAATCCACAGCAGTTTCGGGAATCTTGGTTTCCTACATGCTGATGGATATCACGTAAACGAGAATTGCAACAATGTCCTGGAATCTATTTTACACAATATTTTAAGTGAAGACGAGTATTTGCGAACGTATCGTAGAAGCATCAGTTTTGGGCAGAATGTCAAGAAGGACCTTATACCACTTTTAATCCATACTAAAGATGATAAAACTATTGAATTATTAATAAGGATACTAGTTAATTTGACTATTCCTATCGAATGTTTGCTTTCTGTTGATGAAATCTCCAAAAATGATTTCGGACGTCATACTATATTCGAAATAAACAATTTACTTGCAACAACGAAAATGGCATTTACAGATCAACGAGCAACTAAAGTTGTAATAGATTTTTTAAAAAAGAATTTTGATCTTGAACAAAAGACAATGTTAACCATTGAGCAATGTACAAATATAAGTAACAGCCTACTTTTGCTGAGAAATATATTACACATACCAGAGAAAACTCCCACTCGGAGTCCAACTTTCAACGGGCCTGTTCACTCGATCCAAAACCAAATCATATGGAATATCTTTAGCCAAAGTATCGACAAAGTCCTCATTAAGTTGATGATAATTCCTGACGCGGCAAAATGGGCGGTTACAATGATTCAATTCATAGCCTTGCTTTATAAAAATCAACATGCTGTGACATTACACAAACTCCTCAATTTATGGCTAGAGTCATCTTTGTCAGAAAGCTCTGAGGATAACGAGAGCAATACCTCTCCGCCGGATCGTGGAAGTGATGACTCGTCACCGATATTAACGTCAGATCCCACGTCCGATTCGTCGGACACAGGGGGCAGCGGTAAGAGCAACGACGAACCAAATTCAGTGAATAACGGATGGGATGCTTCTTCTGTTAATAACACCAGTGAGAACGGAAGCCAGACCTCTCAGACGCACAATGATTCTGAGTCACCAAGGAAAAATGCGGAAAATATTGCAGAGGGCTCCATGAAAGATGAAAATGTAAATGAAGCGAAATCGGTAACGTTTTCAGAGAGTAACACGGAAGCGACTAAAGATGTGCCAGCAGGTGAAGACAAAATGAAAGCGGTCATATCGGAAAATTCCGATTGCGGTTACGGTACTCAAATTGAAAACCAGGAGTCCGTTTCAACGTCCAGTAACGATGACGAACTACCCTCTGAGAAGCCGGTTCACCAGAAACCGCATAATCCAAAACAACGGGTCAACACCAAATCTCGTAGCGGGGTAACGGTGCAAGAACGGAAACGTAAAAAAATTGTTAAAAGAGGAAAATCTAACATAATCAACGTACAAGGGCTGTCTCATAAAACTCCAACTGACGACGACATATCTAACATCTTGAAAGAGTTTACTGTCGATTTCTTATTGAAAGGCTACAACGTCCTTGTTCAAACACTTCACAGTCAGATACTCACGAATTTACAACTGGAAATCGATACGTCGCATTTCTTTTGGTTGGTCACGTACTTCTTGAAGTTCGCTACGCAGATAGAGCTGGATCTAGAACATCTCTGCTCGGTCTTGTCATTCGACATCGTCTCATACTTAACCGCCGAGGGCGTCAACTTGTGCGAACAATTCGAGCTGGCCGTCAAGTTAGACGGTAATGGTCTCAAGCCGTGCATGAGGAGGCTCCATTTGGTCGTAACAGCAATAAGAGAATTGGTTCAAGCAATCGAAGTGTACAAAAAGATATCGTTCATACGCAAAGACGACAGGGAGGCGCTCATAAAATTACAAATCAAAATGTGCGAAACCGATGAGCTGAGATCTCTGATGGTGTTGTTGCTCCGCACTTACAATCCTAAGTATCACACCAAACAATATTTACAGGATATAATAGTAACGAATCATATTTTACTATTGTTCTTAGACAATGTGATGAAGATGACCGAATACAATGGCACGACTAATATGGTGGACCATATAAAACAATTCGCTACGCCCGAAATAATGTACCAATACGGGTTGCTGCTAGAGGATTACGCCAGTAACGGAGAGTTTGTGAACGACTGTATCTTCACGGTAATGCACCACGCGGGAGGCGAACTAGAAAGCCTGATAACGTTGTTCCAACCTAAGATACTTAAGACGTTCACTTCGATTTGGAAATCGGAATTCGAAATTTGCGATGATTGGTCCGATCTAATCGAATACGTGATCAATACGTTTATAAAGAAGCCGCACACGTTACAAACCATGGCTGACATTAGGCTTGACACGGAGAACTTTGACGACGAAATAGTTTCGAAGCCTGTCACGACGCCGGAAGATGTCGCTGGTCCGCGCACTGACGATATAGCCAAGAAGAATAACATAATTAGTAACGCCAGTAACGCATCTGTGGAAACGTGGACAGAAGACGAACTAAGCTCATTGAATTGGAACTACATGCAATGTAATTCTCTATCCGATGTTATCGGCGAGATTGTGAAGTTGTTCAAAGATTACGGAACTTTTAAATCTAGACAATCTATTATCACACAGCTGTATAAACAGAACATTATTGATAAAGATAAATTTGAGACGTTTTTGAACGCCGAAAAACAGTGGAACACCAGAACTGTAGAGCTAAACAAGGAGACGAGAGACGACGAGATCGGCAAACTATGCGAGCAGCTGGCGAACGACGGGAAGTCTAACTTCCTAGATTGGGTACAGAAAGTACTTTTGGATACCTGCTGCGCTAAAATATATATCCAGAAGAAATCGGCCACTAAATTCCTGGCCGACCAAGCTGACTGCAAGCTACTCAATTTTGAATTGTTCAAGAAGAAATCGGACTTTCCGTCTTTGTCGCCAGTGTCCTGCCATTCATTGCTGTTCAACCAATCTGTTCCACTGGTGCCGTGGAACTGCAGACAAGCCAGTATCTGCAAAGACTTGAAGTTTCTGCAGCTGTTACACAAGCTCGGCTTCCACATGCCCGTCGATTCCGGGAAAGTGTTTATAAGAATTCCTCATTTTTGGACAGCGGATTTTCTTTTCGAGGTCGCCGGAAAGATAGCGCCCATCGATAAAAAAACGCTGAAATTCAATATAGACGACCTACAGAATAGTATGACGCACGAGAAATATCGAAATCTTATAGTTTTAACTACGAATTATACTAAAGATATTACTATGGTGGAAACCTCTGATAATATTTATCAAGTGCACAAACAAAAGCATCTCGCCACAATGGTCAACTTTACGCCTATGCCTGGCACTTCATTCGGTACGCAAAGAGATGACGAAAGACGCAACTGGTTGGAGGTGGTTAAAAAGTCGCAAGAATATAAGATTACATTGAATTTAGGAAGGTTAGTGGGGGAGGAAGGCGACATACAGCCTGATGTAGTGATGAAGTTGCCGTCGAGCGAGCCGTCGGAGTCGCTCCCCGCGCCGCCCATCAGTGGGAACATCACCGACACAGAGTACTACAACAACAGCGTATGCGAAACAGCTTCGGTGGCGTCCGATCTCACTCGAATGTACGTTTCGGATGAAGACGAGAAACCGGAAGTGGTACTACGGCCTGTCCATGAGGTCAACAACTTGGCTGAATCCAGTGACTCCGGCAACTTAAAACGTCCGCGTGTACAATTTGCGCCCAATTTCTGATTCCATACGTAGTGGATAAGTAGCTGTCGAAACGATATTGTTTAATTTTACCTCAATAAATCTTAATAAAATATACCTAATATTAATTTTATCGTAATTAAAATAACTATCTATTTTGAAAAAAAAAAAATATCTGTGTGAAATATATATTCCAAATGTGTTGTAGTTAGTGCAACTACAACGTGTTTATTACATTATTATTAAAACAGATTAGCTTTTGTGTTTAAACCAATGCATTTTGATAGTACGTAGTAACAATTTTATATAATAATATAATAATATGTATTCTATTAAAAAAAAAAAATTATGTATATTCATTGTTGTTGTACACTTTTTCACCTACCATTTACTCTTCATTTTGGTTTACTTGGAGCTCACCTCATGTACGAATTAAACTCACCTCGTGTACCTATATAACACACAGTTATTTTCTGATGTTTTTTCTGTGCACTCTAAAGTATAATTAAATAAGAATTTCTATACGTTGTAACTCGTGACTATGCGAACACACAGCTATAAGGGAAGTTTCAATAGATTTAATAATAGTTTATTTTTTTATAATTTGTTGTGTTGTGTTAGACCTTTAAACGTAGTGTAAACAAATAGTTTTGATAGAATTGCATTAATCGAAATAAAAATATACCTCTGAA |
| *Clock* | TGAAAGACTAAAATTGACGAAATCGCTTTTGGTGACTTGAACGGTTCTGTCTAAAAACGATTTTGTTGGGCTTAGTGGTGACTATTACATGTTTTTGTTTGTCGTGCAAGTTTTTAAGTAATTTACAAGTATAAATATTTTTAAACAAACTTTTTTCGTTGACCTAAATGCATGACAATGTATAATTTGGTTTCAATTGTGTAAAACTTCGACGCAGTGCACAGTTCGTTATTAACGATTTCAGTACTATCAACAAATATACCAGAAAGACTGTGCTGAATAAAAAAATACAGTGGAGATGCATGCATAATTATTGTCTAATAAAGGCGAATGAGACTTGGTTTTAAAACGAGCCCAAGGAATCGTATTGGATCGCAGCCTGTAATGGCTGGATAAGCAGTAATTTTCAAACTGAGAGAACCAAGTCCAAACAGTATGGAAGACGACGGAGACGACAAAGATGATACTAAGAGGAGGACTCGTAACTTGAGTGAAAAAAAGCGACGGGATCAGTTTAATATGCTGGTCAATGAGCTTAGTTCAATGGTCTCAACGAACAACAGGAAGATGGATAAATCTACAGTACTCAAGTCTACAATATCCTTCCTGAAAAACCATAATGAAATAACAGTAAGATCTCGAGCACATGACATACAAGAAGATTGGAAACCAACTTTCCTATCAAATGAAGAGTTTACATACTTGGTTCTTGAAGCGTTAGAAGGATTTGTCATGGTTTTTTCTGCGTCAGGTCGAATCTACTACGTCTCCGAAAGCATTAAATCTCTTCTTGGTTACAATCCAACTGAAATTCTCAATAAGAGCATATTTGATTTGACGTTTGAAGAGGATCGCCCTAATTTATATAACATTCTACAGAACACAAATACCCCGAATCCCAAAGAAATCGAGATCCGGTTCCAGTGTCACGTGCGTCGAGGTTCCCTAGACTTCAGAGAAGAGACGACATACGAGCTGATACAATTCAACGGACATTTCAGGGCTAATATGGATCATAATGATAATGATGATTCGTCCGGATATCAACAAGATCCTGATTCAAGGTTGCTGTTCGTTTGCACCGGTCGTCTGTTCACACCTCATTTGATACGCGACGTAACTTTGGTCGACTCGAACCGCAGCGAGTTCACTTCCCGGCACAGCCTCGAATGGAAGTTCCTGTTTCTGGATCACCGCGCTCCTCCGATCATCGGTTATCTGCCGTTCGAAGTGCTCGGAACATCTGGATACGACTACTATCATTTCGACGATCTGGAAAAGGTTGTGACCTGCCACGAGGCATTGATGCAGAAAGGTGAACTCACATCCTGCTACTATCGATTCCTGACGAAAGGTCAACAGTGGATTTGGCTTCAGACTCGTTTCTACATCACTTATCATCAATGGAACTCGAAACCGGAGTTTGTCGTATGCACTCATCGAGTTATTAGCAATGCTGACATCGCCAAAAACGCCAAGCAAGAAAGCGCCGAACCTGACAACGTGAGTGAGGCGGACGCCAGCCACGGGACTATAAAAGATGCCCCTTCGGAAGAGAATAATCTGATGGTCATGTCGCCATCGTACATGTCCGACGCTAGTGACGCCTTCACAAACTCCTATCACAGGACGTCTCAGCCACCGTCCGTGAAGTCCGCGTCGGCTTCGTGCGCCTCCGGTGGTGCTAATAGTGGGACCGCCCCGCCCTCTACCACAGCGTGGCAGCATTCGTCGAACACTCGCTTCGCCCGTTCCGACACCACATCGGCGTCCGGGGAGTCTAGGTCGTCTCAGAGGAACAACTCGCACGAGCTGGCGCACGGTGGGGTCGCGAACGTTAACGAACATCCGTGTTACGGTAGCATGATGTTCCCGCAGTCGTACAAAGCGGGTTCGGAACCGGCTCTGGTACCGCAACACGGCATCGGGGCTCAGTATCTAGAGCCGGACCCCTACGTAAGTGCGGTCAGTCTGCCCGGCGTTCTGCCTTTACCTTTGCCGCCTCTACCGATCATCGTGTCCCCCGATCAGGCTCAGATACAGTTGCAGAGAAAACACGAGGAACTTCAGCAGATGATCTTGAGACAACAAGAGGAGTTGCGACAGGTGAAAGAACAATTACTACTGGCCAGGCTTGGTATACTGCAACCCCTTATAAATGTCCCGAATCCGTACGCGAATACAGAAGAGACACAGCAAATGCAACGTTTGCCGACTCAAGTAAGTTACGACAGGCCAGCTCAGCGCTCGATAAGCGGATACTCTCAGACACAGCCGCCCCCACATCTGTCAGCCCTCCAGCCACACTCGCAACTTCAACACCAACACCAACACCAACATCCAAATCAACACCAACACCCGCGCATGCTGTAAACGACACCGACCAACTCGTTGGCGATATTGGATCAAATAGTTGGTGCTACGAAACACCGGCGTTCTATTGCAAACTATTGCAATTCAATTTGCGGAACATAATCGACGACATATTTTATGTGATTATGAGTACCATGATTCTTACCTTGTCTAATGCCATATAAGTACATATACTTGAATCTACAACCTGATGTTGTGAAATCATGTTTCATACCTTATGTCTATTCATAAGGTCGTGCAAATGGCCCGTTGCGGAACTAGTGATGTAAGATTTTGTTATATTATAATTTGTTACGTTAATTTTGTTTTTGAAGTGTCGCGGACATTTTTTGTCAAATGCCAATCGGAAAAAGAAAAGAAAAAGAAAATATAATGGTTTTTCTTTTTTTTTCGTCCGTTATGGTTGAATATTTGTTTACAAGAAAAGGCTGTGCGTTTCCATTACTATTATTATGATTAGTTAGAAAATACTATAAATGTTTTTTTCTATTAAGTTATTATATTACGTTTCGTATGTTGTTTATGAATACACGATAAAGTTTAGAACACATTATAACTTTATATCTTATTGTCGTCTGCCAGTGACGTATCATGCGGCGTTTCACAGTGAAACTATGTTTTCTATTATTATTATTAATAATAAAACACGTTTAAGTCTATACTTATGTACATAAAATTGGAATTGATCTCGATGTAAATTTGTACCCAAATAAATTGATGG |
| *Cycle* | TGACAGACACGAAAGTAACATCGGATAATTTGGAAATTTATTTTATTTTAATTAATATTTAAAATTCCGTAACATTCAACCGGTTCGTCGGTATTTGTATCTGTAAATCAAACGGATATTGTGCTTAGATTATCAAAAAACATAACTCGAGTGCTAGTGTTAAAGAGTATTGACTGTTGCGGAACAAGGGCAAATTCGTGAAGTTTCCATGGTCTTATACGAGGAATGGCCGATTTTATCGACGAGGCTAGTACATCACAACGTGGACATGCGAACCCGAGTGCGATACAGGCGTATGAGATGACACCGGAAGGGGGCGTGGGGTTGGGCGGCGCGTGCGCCGACTCGGCCGGCGCCCTCATCACCCCGCACCCTCCCCTTCATCATCCAGTGCCGCAGACCTCACAGCAACTCCATCATGATCCGCGGAAAACAAAACCCAATCACTACGTTCCTGAAAACTATGAGATATCGCCGTGCGACTCGCAGCGCCAGTCTCCACACGGACACACGCCGCGGACTCGGACCAACAGTACTCGTAAACGGAAACCATCGTCCTATGGAACTGGAAGCGCTTATGATGACGACGAAGAAGATTCAAGATCCACAACAACTACAACCACGGCGACCACGCGCGGCACTCCCGACAAAAAACAAAATCATAGCGAGATCGAAAAACGGCGACGTGATAAAATGAACACTTTTATATCGGAACTGAGCGCCATGATACCGATGTGCGGAGCCATGGCTCGGAAGCTGGACAAGCTGACAGTCCTCCGCATGGCCGTTCAACACTTGAGGACGGTGCGCGGCGCGCTCTCGGCCTGCCCCTTGACGGCCCGGCCCTGTCCCACTTACCTCACTGAGCGAGAACTAAACGCACTGATACTCCAGGCGGCTCACGACTGCTTTTTGCTCGTCGTGGGATGCGATCGCGGCAGGTTACTTTACGTCTCGGCTTCGGTCAAAAATATACTTCATTACGATCAATCGGAACTATTAGGCCAGAGCCTGTTCGACATTTTACATCCAAAGGACGTCGCTAAAGTAAAGGAACAACTATCATCGTCAGACCTTAGTCCACGAGAAAGACTCATCGACGCAAAAACGATGTTGCCGTTGAAAGCGGATGTAGTAGCCGGCGCGTCCAGGTTCGGTCCAGGGGCACGTCGGTCGTTCTTCTGTCGGATCAAGTGCAAATTAGACACAGAAGAAGTCGAAACTCCCCCGCAGCCAGTCAAGGAGGAGGTGGAGCCGGTGGCCAAGATGCGGAAGAAACACTCTCACGAGAAAAAATACTGCGTCGTGCAATGCACAGGTTATTTAAAATCGTGGGCGCCAACGAAGATGTGTGACGGTGCTAGTGCGGAAGGGGGAGAGGAGAGTGAAGCGTGCAACATGTCGTGTCTAGTGGCAGTGGGCCGAACCTTGGGTGGTTTAGCTCCCACCACGAACTCTCCGACATCCATGCCTCAAACCAGACACCTGCAATACGTCTCACGACACACTACCGACGGGAAATTCTTGTTTGTTGATCAGCGGGTGACCCTCGCGCTAGGATTCTTGCCCCAGGAGTTGCTGGGCACTAGTCTGTACGAGTACGTGCACGGGCCGGAGCTGGGCGCCGTGGCCCGCACGCACAAGGCCGCGCTGCTGCAGCGCGACGCGCTCCACACCCCGCCCTACTGCTTCCGCAGGAAGAACGGCTCGATGGCCAGAATACAAACCCACTTCAAACCGTTCAAAAATCCGTGGACGAAAGACGTCGAGTGCTTAGTGGCCAACAACACCGTCGTGTCGGAGTCCCAGGTGTCGCTGCAGCAAGACACGACGCAAGCAGCGTTTGATATTTACAAGCAAAAATCGGACGTCGAGATGCAGCGCCTCATAGACTCGCAAGTCGAGTCCCATAAGATCGGCAGCGCCATCGCCGAGGAGGCTCTGCGGCGGTCGTCGACTGATTACTCGCCCGACCTGCCGACCGAGTTGCTGCAAGACGCCGTCTTCAGTC |
| *Shaggy* | GGGGGCCGAATCTAGCCGAATGAACGAGCGAGTACAAGTCAAGCCCGATGAAACTAAAATCTGTAGTACACTTTCAATGTAAATTTTGAAAAATTATCAAAATGATACTGTAATTCTGTAGTGGAACTATTTTATTTTGATTTTTAATCTCGTAGTAGTGGTATTGTTTCAAATATTTCTTCATTGAATTCGTATTTGACAACGTAGTGAGTTCAGTGCGAGTGCAAGGGTGGTAACGAGAAATGTGAAATATCTATGAAGTTCGGATTTTTAGGCGGTTTATTCTATTTCAATAATGAGCGGACGCCCTAGGACCACGTCATTCGCCGAGGGCAGCAAATCCGTTCGAAAGACTTTCGACAATCAACCTCCAAAACCACCTCTCGGAGGCGTAAAAATTAGCAGCAAGGACGGCTCAAAGGTGACGACTGTGGTAGCGACGCCGGGGCAGGGCCCTGACCGGCCTCAGGAAGTGAGCTATGCTGACATGAAATTGATCGGCAACGGCAGCTTCGGCGTCGTCTATCAAGCCAAACTATGTGACACCGGCGAGCTCATTGCCATCAAGAAGGTGCTCCAGGACAAGAGGTTTAAGAACCGAGAACTTCAAATCATGCGACGGCTGGAGCATTGCAACATTGTGAAACTGAAGTATTTCTTTTATTCCAGTGGCGAAAAGAAAGACGAAGTGTACCTGAATTTGGTGCTGGAGTACATACCCGAGACCGTGTACAAAGTGGCGCGTCATTACTCCAAAGACGAACAAACCATACCCATTAGTTTTATAAAGCTGTACATGTACCAGCTGTTCAGAAGTCTGGCGTACATTCATTCTTTGGGCATCTGTCACCGAGATATCAAGCCGCAGAACCTATTGCTTGACCCCAAGACTGGAGTGTTGAAGCTGTGCGACTTTGGCTCGGCGAAGCACCTCGTTCGCGGAGAACCAAACGTTTCCTACATATGTTCGCGTTATTACCGCGCTCCGGAGCTCATCTTTGGCGCCACCGACTACACCACGAAAATTGACGTGTGGAGCGCGGGATGCGTTGTCGCTGAGCTGTTGTTGGGTCAACCGATATTCCCAGGAGATTCCGGGGTTGACCAATTGGTTGAAATTATCAAGGTTTTAGGGACACCGACAAGAGAACAAATCCGAGAGATGAATCCAAATTATACAGAATTTAAGTTTCCGCAAATCAAGAGTCACCCTTGGGCGAAGGTGTTCCGCGCGTGCACGCCCCCGGACGCCATCTCGCTGGTGTCGCGGCTGCTGGAGTACACGCCGGGCGCGCGCCTGTCGCCGCTGCAGGCCTGCGTGCACAGCTTCTTCGACGAGCTGCGCGAGCCCACCGCCCGCCTGCCCAACGGCCGCCCGCTGCCGCCGCTCTTCAACTTCACCGAGTACGAGCTCGGCATCCAGCCCTCGCTCAACGAGTTCCTGAAGCCGCGCAGCGCCGCGCAGTCCGAGCCGGCCGCCGCCTCCTCTGCGCCCGACCACGACGCGCCAGGCGAGGCGGCATCGGGCGGCGCGACGGCCGACGCTTCCTAGACCCGCGCTCCCCGAGACGACCTAGCGTACTTGATAGAGCTTGAGCCGCCCCGGTCGGCGGGAAACGAAAATCCGCCGTAGATTCTGACCCGCGATGTGATCACTTTGTACTTTTGTAAGCTCCGATCGTCTCCTCGTGTACATACCTCGAACGCCGAGTCTGAGGCCGTCCGTCCGACGATGGACGGATTGCTCGATTCCGTTTTTATTAGTGACGTCGTCCGTGTTGTCCGTTCGCCCTATGAGATAATTTCGCTTTTATCCGTAGCGGTCGCAGTTAGCGACCACGCTTGGGCCGTGTTGTTGTAGGGAAGACATTATTCAGTAGTGCATTAGTGTATTTATTGTTGGGAGTCGTATCGCTAACTCGACATATACATAGAGCTGAATTCGACTAAGCAAATTAAATGTTTTGTTGCAAACATAGAAAGCATACTTTGTAAATTTTTATAGCTAGCATAAACTGATCTCTCAATATAATTTTATTGATTTCTCAGAATATAATGTATTTGTGTAAAATATTTCAAGGAATATAAATTTTAGATGTATTTCACAAATAATAGATGTTTCATTTCCCGGCAGTTTCTGTGTTAGATATGTTTTGCTGTGTATTCGTTTATCCAGAGGATAGTTGTAATGTACCTACATAATGTGCCGTGTAACCTAATGTCACGACATAGTTTTTCAATAATATTGTATTCTATATTCAATAAATGATATTTCTATAATATATAATAAATTTGTCATTGCGAACGCTATTGTTATTAGTAGTCATTTATTAAGAGGACGGTTATAGTAATGCTAAATTTCATTGTCATAGTGTTTAAGTATTTGAATAATTTCGGTTTGCACTTCATTTTTGCTTACTATTACATTTTAGGTCCTGGTAAAAATTTCTTAGCGTAGACTAACAAAAACTTACTCACCCTGTAGAAGTAGCCTATTCGTGAAATGTCGATTGCTTGTTGCACATTTTGTGGAACCAAAGTAGTTGAAATATTTACACATCTCAGAGCTTTAAACATAAAAAAAAACAACAATCAGCAGCTCGCATAATTTAACAATAAACGTTTGAAGTTATAAAAGTTTAATACTATGTGACGTCATCAACTAACGAAGTAGTGTGGGCAAAAAAACAATTGTCTTGTGTAATGAAAATTGTACATTACATTCGCGATATGTATGAGCGATCAATTGACAAATTTTATTTTTGGTATCTGACGGAAATCGCCAGTGTCCGACCGTAGTGCGACATACACATGCGTGTATTCATGCATACTATGAATGTACCTTCGTACTGCTATTTATAAATTATTGTATGTTAGGAGAGCGCTCGGCGCATCGATTTCAAATCACACATAGTGAAGCTTAGTTTCTCATCGTTTAGGCTTATCGGTTTTCAGAAACACGTTTACGATTTGTGTAAATATAATATTATATTATAATTATGTTAACGGAGCACCATCGGAGTGACGCGCCGCGCCACGGCGACGTCTCGGCGCCACTGAGGCTCGGCGCGTCACCGGTGCCCCGCAGCGGACTCAGTAAGAGACACGGTAGTTTAGATATGCATATTTTATTAAGTCTTATCTTGTATGAATTTTGTAATCGACGTTGCTTCTAAAGATCATTTTGTGTAAAAAAAATTATAAAACATACTAGTGACCTGAATGTTCGAAGCTATATTTGAACGTCTTTTATCCCACTGAATTCTCACTTTCACGATTTGCGTGCACATTTAATTACATGAGAATTTCGTATATACATAGTTGGAAAATGGAGTTATGATGACTTATAATAAATTATTTAAAGTAAAGTTTTCTTATTACTATTACTGTAATATTAAATTTAATAGTGAGTGAAATTTGTATTTTGTTTTAATATTTGTTTTACTGAATTTGAATACGTCGTAACACCCATATCGTTGTTAGTTATGAATGAAAATAACATAATTATAAAGTAAATATTAA |
| *Double-time* | TCCCATTCCAGCAGCAGCTCTATGTTCTATCTAAGTTTTGTTCAATTTACAAAAATTTGGTTCCTCGTCCATGTGTTTCTGCCTGTAGATAAGTCCAATTTGCAACTTATTTTAGTTAAAAATATAGATTTTTTTACAGAAAATTTACTAAGATAATCTCTACAACAGCTCAGATACAAACAAAATTACATCAGAATTCATGTTCAATTTAATTTTACTAGTTAACTTGTGCGTCTATCGCAATATGGCCGTCCTGTAATGAATTGGTGGTCAGTAATATTATTTGTGGTTCTATCTCTCTGCTAATATTAAAATGGAGCTTCGTGTCGGTAACAAATACCGACTTGGGCGAAAAATTGGCTCCGGATCTTTTGGAGATATATATTTAGGAACAAACATTGTCACAAGGGAAGAAGTAGCCATTAAGTTAGAATGTATCAAAACGAGACATCCACAGTTACACATAGAAAGCAAATTTTACAAACTAATGCAAGGAAGAGTTGGAATTCCTGCAATTAAATGGTGTGGGTCAGAGGGTGACTACAATGTAATGGTAATGGAACTGCTAGGTCCGTCATTGGAAGATCTCTTCAATTTTTGCTCACGTCGTTTTTCTCTCAAGACAGTACTCCTTCTAGCAGACCAACTAATTTCCCGCATTGAGGACATACACTACAGAAATTTTATCCATAGAGACATCAAACCTGACAATTTTCTAATGGGCCTAGGTAAAAAAGGAAATTTAGTGTATATAATAGATTTTGGCTTAGCTAAGAAGTACAAAGATCCTCGGACTAATCAACATATACCTTACAGAGAAAACAAAAATTTGACAGGAACAGCAAGATATGCTTCAATAAATACACATTTAGGAATTGAGCAGTCACGTCGAGATGATCTAGAGTCACTGGGCTATGTGCTCATGTACTTTAACCGTGGTAGTTTACCATGGCAGGGGCTAAAAGCAGCAACAAAACGTCAAAAATATGAAAGAATATCTGAAAAGAAACTGTCCACTCCTTTTGATGAGCTTTGTAAGAACCATCCAATTGAGTTTCAACTGTATTTGAAGTACTGCCGGCGACTTCGTTTCGAGGAGAGACCCGACTACGCCCATCTGCGTCAGTTGTTTCGCACCCTCTTCCACCGGCAAGGATTTACATATGACTACGTGTTTGATTGGAACATGTTAAAGTTTGGAGGACTGCGTGATAACCATCAAACAAGTGGTATTGATCGCGCTCTTAGACGTCTTGAACAAAATCAGGAACAAGAAAATACCGCTGCGACAGCCGGCCAGCCCAGTAATGCAGTGGCAGCAATTTCGGAATGGCGGTGAGGAGAGATACCGCTGACTTCACGCTGATAACATCTCTCTAAAAGCACGTTGGTATTTGTGACCAGTATGTTCTGTGTCGCAGAATGTACCTTACACAGACATAATTGTGACAATGAAAGTATAAGGACGCCTCTGCAGATGATCAGTACGGCAGGACTTGAGGCGTGAGCGTGAACTTCGACTACACATTCGCGATTTTGAAAGCGAATCTTTATATTAATTAATAAATAAATAAAAAGATAAAATGATTTTGTAAGCATACAGGTTAACATTCATATTAATGTAAATTATTTAAATTTTAGGCTAAGCAGGTTGATAAGTGGGCCGATGAACTCGTAACTTTTCTCGCAATTTTTAAATATGTAACGTTAAGTTATGTAATAAATAACGTTTAAGGCCTACCCATACATCCATTGTACTTTGTGCTCAGTTATATTTAGATATAAGAGAATTCAATAGTAACATTTGAGGCGTTCGTTCAGAAAAAGAACTAACTGCCCGGTGTCAAATTTTGATGTTCAGTTTAAATAGAATAATCTATAAATATTCTTTACAAAAGGAATTATTTGTCTTATATCTAAATACAACACTCGTATATATTTGGTAAAACTGACTGATTGAAAACAATTACTAAAAACGCAGGATGCAAACTGTAGAAACGAGTTTATCAGACTTTTCGATTCCTACTGCGTTAATTTTGCAACTTTAAGTAAGTTACTACAAATTGAAAGTAGGAACGGTAGAAAAATAATTGCTCTAAACCAAATTTGTCTGCACATTGTGTAAAATCATTGCTCGAAAAGGTATAAAACTGTAAATAAACAAGTACATTCTCTGAGTGCAATATAGTGATGTTTTCTTTAACTATTCAACATGTAGTTTCATTTGATTTTGTCTTCCAATTATAAATATTATTTACAACTTACAATAACTCAAATTAGTAACGTAAATTTGTGATAAATTTTATTTCCCTTGAATCATGTAGTCACTGCCGTACACAAAAAAATCGTAACATGAGCAAAAATAAGTAATGATAGCTAGAACGTACCGTACCGAGTGTACAGTGTTCTACTAGTTCAAAGTGAGTTTTATTTGGAGATTAGTAACGTATGCAGTTTAGATTTTAAACCATAATTTCTGTTATGGTTATTTTTTTATAAACTTTGCGAAATCTATGATCTCACGCAGATTACCAAATTTCATAAATCATAATTTAAAAAAAAATCGGTTACCATTTCAGTATCTGGACACCGAACAATAAAAAAAATTACAATCTTTGTAATTGTCGTAAATTGTAAATATTATGTAGTTATATATTTTAGAGCATTTGTGTAATGTAATCTAGCGTCAAATCTACCGCGCTGTTAATACTAAAACTGTGTTGCTATGTTATACCTTACCTTATTTAACCAAGGTAATTTCTATATCCAAAGTACATCATATACGAATATTTCTCGTTCAGTTTTTTATTGTCCTTAGAATTACTTTATTTTATTATAGTCGTTTTGTAATAACCATTGTTTTAACATAAATACTTTCAATTTGCCATTGTAAGCATCAGGCATTGTCAATACGTTAATCGTCATCTCATTACTGAGACATTTTTGTTTGATTTATGTTATTGAGCTGGAGAAAGTTTTGTAAAGTGGAACTTGTAAATATCATATTATCGATTTGACATAAGTGTTTGATTTTAAGGCACACGACATTCAAGATCCCTCTTATTAAGGACAATTCACTGCGTAATTTAATAATTAACAATTGGCCCACGAGAGCGTTTATATTTCACATATTTACATTACTAAAAATGTATTTAACAGACATAACTCATCCACACTAATCTCAAAAAACGAGTTCTGGATATAACAAAATTGTAAAGTTCAAAAACGCATTAAAAGTTTTAAATAGTGAACCTTTTCATAATTAATGTAACACATGAATGATTTCAATAGATTCGAACTTCATATAATAGAGGATCATTCAACAAAGTTCGACAAAGTTCACGTTGGGATTTCATTTTTAAAAGTTAGTTAAAATGTGTAGACTGTTATGTCTACGCCTTGTTTAAGCAGACTTCATGAATATTGTAATATCTTAATTTGTAGTTGTAAGTTTTGGCAAATGAAACCAGCCAATGTCCGATGCAGACTAACTTCAAACAAAATGACAAGTTAATTGCAGACAAAGGAGTAATGTCATCAGCGGTGACGATCATCCGCACCTGGACAAGAACAGTGTTTGTGAATGTGTAGAGTGCTACAAAATGTGTATTAAATGCTTGCCGCGTGTGACGGTCGTGCTTTTCTTTTTAACGATAAAGTATATTCGGAATTACAAAAGACTATTGACTTAAATTTTGTATATAATACATTTTTAAGGTAAGCTACTTGGATAAACCGAATACTGTTTTTTTACAATATCTTAGGTAATGAAATAAATAATAACTGTTGTAGTGTTAAAA |
| *Vrille* | GCGCAAAGTGTAGACGTGTCCAAGCAATTGGAACAACGCAACGCCATTCGCATTGGATATTTCGTCATAACAACATTGTGACAGTGAATTTACATTTATATTTTTTCTGGACGTGAAAAGTGGGAGTGAAATTTGACATTTTTAAATTTTGAAACTGTGATCTTGACCGTTGAGCGGTTGTGATTTTTGTTTTTTTTTTTGTGGATTTTTGACAATTCGACCGCCATTTGGATAATTCCAGCAAAGGAGTTCTCAAACACCGCAAATATTTTTAACCACAGTGAGGATTCGTGCGGACCAGTCACAGTCCGCCGAAACATGGTTGCCGAGTTCATCCTGAGCCAGCAACAGCTGCTGAGCGCAGGGGCCGGTGCAGCCCTAGGGCCCGCCCCAGTGCAGACCCGCGCTCCTCCACCACCGCGCGCTCGTCTCTCCGTCTCGCCGCATTTCCCGATGGACCAAGCCCCGCCTTCCAACGGCAGCAGCGGCGACAGCCACGACTACGGAGCCTTCGACTTCAAACGCAAAGACTTCTTCGGGCAACGCAAACAACGCGAGTTCATACCGGACAGCAAAAAGGACGACGGCTACTGGGACCGCAGGAGGCGCAACAACGAGGCAGCCAAGCGGTCTCGCGAAAAACGCCGCTTCAACGACATGGTCCTGGAACAGCGCGTCGTCGAACTCTCGAAGGAAAACCACGTCCTCAAAGCACAATTAGACGCCATAAAAGAAAAATACGGAATATGTGGAGAAAGCTTGATAAGCATAGACCAAGTCTTAGCCACCTTGCCAACGTGTGATCAAGTTCTATGTGTGACAAAGAGGAGTAAACTTTCAGGCACAGCCTTGTTTCCGGCCGCTCCTCCTCCGCCCCCGCCGGCTCCACCAGCTTCTCCCCCGTCTCCCCCACCACAACGCGCCCCAGAGCCATACCAAGAAAGGATTCCCGTACCCGAAGCATACTACCCCCACCCTGCCCACTACGAACCACCCGGTTCAGTTCTCAACTTGTCCAGATCTCGACGTGCCCCGTCTCCTTATGAGCTATCTTCCTTATCTGGATCCGGAGACGAGACCGCCGAGCAGTACGCCCCAGAAAACAACTGCCTTCCTTTGAAACTGCGCCACAAATCTCACCTCGGCGACAAGGACGTCGCTAGCGCTCTGCTATCCCTCCAGCACATAAAGCAGGAGCCGGGGCCCAGGTCGTCCCCCTCCTGGGACGGCGAGGGTTCTAGCGACGAGCGCGACTCCGGGATCTCACTAGGAGCAGAATACAGACCGCAACCGGAAAGGCATCCGGAGGAGGAGGACGCGCATTTGAAGGCTGAGCTGGCACGGCTGGCCACAGAGGTGGCGACGCTCAAGAACATGATGCACCAGAACAAAACCCGCGCGCACGAGCACTGAGCGCGCCGGCCGCCGCCCTCACCCGCCTCGCCGCCCTCGCCGCTCTTCGGCTGACCAAGTTACCACGTTGTAAAGAGATGTTATATTGTATGATATCGTGCAGCTGTAATCGGCTAAGCCGTCTCGCCTATCGTAACCCTTCCTAGTAGTTGTATGTCATACTCGTAGAATAAGGACGCGCGTACCTCCAATGACGCCCGATCCTCACACTACACACTTTTCGAGCAGATAAAACGTATCTACTCAGTGATAGAATTAAAAATGCATTTTTTAGTGATGAGATATGTATATGTAATATAAGTTATAGAAGTAGATCTGTTTCATCTATTCGAAGATTCGATAGTGAAAAACGATGGCTAAAGACTATTTCTCTTCTAAGTTATAGCTTGTAAGATCTAATTAAGTCCCACTTAGTTTGTTATTTATGGATATAAATATGTATATATCTCGAACCGAGAAATCCGTTAAGTTCCAAACCAAAAAAAAAAGATCCTCTGAGTCGATGGAATCGCGATGTATTTAAGATGTAAACCCATTATCACGTCAACAGATTGTATTTAATATTCAGTATAAGTATACCAGTGCCTTTGTCAATACATACATACCTTTATACTAATAGCTACCTAAAGGAAATATTTTTGTACATTTATAATAAGAAAGATGCGATTTCATTCATTATTTATCATTGTCAAGTCAATCCTTAACACGTGTCGCCGAATCAGACGAACTCGATACGAAAAATGGTTTTAGATTTTGTAAATAGTTAAACGTGTGTTGGCTATCGAGTTAGGATGTGACTGCGAAACATGATAGGGTATATACAAAAACATTAAATGTCGAATCCTAAGAAGGCTAGTTGAGTATTTCAGTATATGTTCCGTAGTATTGCCCTATAGGGCATGTGATAGGACTGAAGGGAAGTATGTAACTAAGTATTTAATTTATTGTTCGGACCGTGAAGGAGGATTTTACAATTTATTATCTTTGTATAAGAAAAACTACGATATGAATA |
| *PAR-domain protein 1 ε* | CGGGACGACACGTGCATCGGAGGAGCGCCGCGCCGCCAGGGTGCTGGTGCAGGCGAGAGTGATCGCATCGCGGCTATTATGTCACCGTAATTAAAAAGTGTGCCGTGCGTGGTCGGAAGCGTCGAGTGGTGGGTGGTGGGGAGCGCGGGAGGGGTGCGGCCCAGTGCGCGATCACCAGTCGCGTGCAGCTCGTCGCCGCGCACGCCTGTCGCGTGCCCTCTCGCGCCGCCGCGATGAGTGATCACTGAGAGTGCGCCTCGTCGAGCATGGAGTACCAGCACCAACCCATCCAGCCGCTCGGGCCGCCGGCGCCTCCGCCGCAGCTGCTGCCGCCCGCGCCGCCGCCGCCCCTGCCCACGCTGCCCCAGCTCCAACACGGCCACGTCCAGGACGACGACCGCTGGAGCCAGTATCACATCTGGAGACAACACGTCTTCGTCAACGGCAAGTCCGTCAACACTAAGGAGACGCTCGAAGACAAGAAGGATGACAACGACCTCTGGGAGGCGCAGGCCGCCTTCTTGGGTCCCAACCTGTGGGACAAGACCCTGCCCTACGACCCCGATCTCAAGTACGTGGACCTAGATGAGTTCCTGTCGGAGAACGGCATGCCGGGCGAGGGCCTGGGCAGCACGCACCTGGGCGGCTCCGCGTTCGGGCCGGCGCTGGGCCTGCAGACTCCCATCACCAAGCGGGAGCGCTCTCCCTCGCCCTCCGACTGCATGAGCCCGGACACCATCAACCCGCCGCTTTCACCAGCCGATTCCACGTTCTCGATGGCATCGTCGGGCCGTGACTTCGATCCTCGGACGCGCGCCTTCTCCGACGAAGAGCTGAAGCCGCAGCCGATGATCAAGAAGTCCCGGAAACAGTTCGTGCCCGACGACCTGAAAGACGACAAGTACTGGGCGCGCCGACGGAAGAACAACATGGCGGCCAAGAGGTCCCGCGACGCACGCCGCATGAAGGAAAACCAGATCGCACTGCGGGCTGGATACCTCGAGAAGGAGAATATGGGCTTACGGCAAGAAGTGGAGCTGCTAAAGAAGGAAAACCACATCCTGCGTGAGAAGCTGTCCAAGTACGCGGACGTATAGGCCGCCCGCCCCACCCGCATAGCACAAACACGCCACGCGACATGTGTTGGGCGCGCGTCCGGTCGGCGAGCGCGAGTGCAGCGGCGACACCCCACCCCGCCCCCGCTCACCACACGACTTGCCGAGACAGCCCCCTGCGGCCCTGCACCGAAGCGACCCGTCACCCCGAGCTGCCTCTGCAAATCGTCCGTCGTTCTTTAAATAGGATTAATGCATATAAAACAGTTACCGTTAAGTTTAGTAGAGTAGGTAAAGGTTAACCAAAGTTCCGTTGAACTCTGACGGCTCACCCGACACGCTCGTTCGCTGTACTTTGTAATTCATGTTGTAACGTTTTATTAAGTATTTCAAAATTAAATATTGAAGATGTTAAACGAAATTGCGAGTTACAATATTTAGAGTAGGGTGTAAGAGGAGAGAAGTACTAGGTAGTAGAGCGGACGTTGCTCAAGTCGTACCGCGCCGCCTGTATATACGAGTAGTAATAGTTGTAAAGGGATCGCTTCCTCCCGCTCCGTGCATGAGATTACCGCGCGCGCCTCTCCGTGCGGGCGCCGCGGCCGGGGCGCGGGGGCCGGGGGGCGGGGTCACGCCGCCCGCGCCCCCCCGCGCTCCAGCCGCGCCCCGCCCGCGCCCCGGCCGCGCGCCTTCTCTCGTTGTTTCAAACATAGTGCTTAGTTTAAGTAGTGCGTAGATTCCGTAGGGTAGTCGTTATTAGAATACTTTTGTTATGGCTGTAGTTGTTAACGTTTTGAAAGTTTATATATAATTTGGTTTAGTTGTAATGTGACTCGCGACAGGCGTCGCCCGACGCGACTCCCGCACTCTGTTTCGACTCGTGCTAATCTTATAATATATTTTTGTAGGTTTTTGTCGTTGATAAATGAGTCAATTGAATGTTTATTGAATTGATGAATAAAAATTATAAATGTTTATAAGTAAGAATAAGAAGTTGTTGCATTTGAAAGTATTTTATGAATACAAAACGAGAACAATAAAATGTTTTTCTATGAATGTATCGAGGTGTGGGGTCCGCCCGGAACTCCGCCGCTTCACCTCGCACACTCTTTTATATTCCATTTTGTTTGTTAAAAATATACAAGGAATGCATAACGCCTGCAAGATGTTATAATCATATAAATATGTTTGCTAAATATATGTATGTTCTAAACGTATAATATGTACGAGTAGTGAGAGCACTGGGCGCAGTGCGCAGTGCGCAGTACGTCTGCCGGGGGGTGAGGGCGGGTGCGCGGCGGCGGGGGCGCCCCTCCCACTGCGGCGCTCCGTGACACGCAGCAACATTAAGCCATCTTCGGTTTACATACTATCTCTAGAATTTTTGTTTCACTTAATTTATATGGTTCTATGGATTGAAAATAAACGTTAATTGTTGTATGGACGCGTAGTTTTGTTGAAGCGCACGAGGCACGCGAGGCCCGGCCCGCGCCCTCTGCTCGCTAACGACCAACTCGTCCTGGATCGGTGTTTTATTTAATAAATCGTCGTAAATTACCCAGCG |
| *Slimb* | CGTCATTCGAACGATGTGTGTTTAGCGATTGTGGTTTTGTATATATTAGAGGTGTTTTAGTGGACAATTATTAATGTTAAATAATAAATAGTGAAAATGGAGACTGAAAAGATGATAGAGGATACAATACCACCTCAGGTGACGTCACTGACAAGTTTGGACAGCGGCGTCAGACCGGTGCCCAGCAGCCCTGCGTACGCGGCGGAAAGGGACGCATGCCTCAACTACTTCACAAGATGGAACGAGACAGATCAGGTGGAATTCGTGGAGCAACTCCTGGCTAGGATGTGCCACTACCAGCACGGTCACATCAATGCCTACCTCAAACCGATGCTACAAAGGGATTTCATCAGTATGCTGCCCAAAAAAGGCCTGGACCACGTCGCAGAGAACATACTGTCGTACCTGGACGCGAGCTCTCTATGTGCGGCCGAGTTGGTTTGCCGCGAGTGGTTGAGGGTGATATCGGAAGGGATGCTGTGGAAGAAGCTCATCGAGAGGAAGGTCCGTACGGATTCGCTGTGGCGCGGCCTCGCCGAGCGGAGGGGATGGATACAGTACCTGTTCAAACCGAAGCCTGGCTGCCAACATCCGTCACACTCATTCTACAGACAACTATATCCCAAGATAATAAAGGACATACAATCTATAGAGGACAATTGGAGAATGGGAAAACATAATTTACAGAGGATAAACTGCAGATCGGAGAATTCCAAAGGCGTATATTGCTTACAGTACGACGACAATAAGATAGTATCGGGCCTTAGAGATAACACAATAAAAATATGGGACAGAAAGACACTGCAGTGCGTCAGAGAGCTGCAAGGTCACACGGGGTCGGTTCTGTGCCTGCAGTACGACGAGCGCGCCATCATATCCGGCTCGTCGGACTCCACCGTGCGCGTGTGGGACGTGTCCACGGGCGCCATGCTCAACACGCTCATCCACCACTGCGAGGCGGTGCTGCACCTGCGCTTCTGCAACGGGATGATGGTCACTTGCAGCAAGGATCGTTCGATAGCCGTGTGGGATATGACGTCGACGACGGAGATAATGCTGAGGCGAGTGCTGGTGGGACACAGGGCCGCTGTCAATGTCGTTGACTTTGACGAGAAATATATTGTCAGTGCGTCCGGTGATAGAACTATAAAAGTGTGGAACACGTCGTCGTGCGAGTTCGTGCGCACGCTGAACGGGCACAAGCGCGGGATCGCGTGCCTGCAGTACCGGGACCGGCTCGTCGTGTCGGGGTCCTCGGACAACACCATCCGGCTGTGGGACATCGAGTGCGGCTCGTGCATCCGGGTGCTCGAGGGGCACGAGGAGCTCGTGCGCTGCATACGGTTTGACAATAAGCGAATCGTGAGCGGCGCTTACGACGGGAAGATCAAGGTGTGGGACCTGCCGGCCGCGCTGGACGTGCGGACGCCGCACCAGGACCTCTGTCTGCGGACCTTAGTCGAGCACACGGGACGCGTGTTCCGTCTTCAGTTCGACGAGTTCCAGATCGTGTCCTCGTCGCACGACGACACCATCCTCGTGTGGGACTTCCTCAACTACAGCGGCGCCTCGCCCCCGCCGCGCGCGCGCTCGCCCGCAGATAGGGACCTGTTCGACTAGCGCCGCCCCCCGGTAACATTCTAAGACCACAATGAGACTACGTTTTTTTTTTAAAAAGTTTAAACGTTAAAGAGAATTAATTATTAAATTAATGAGGGATGTAAGTAATTAAATAGATTAAAAAGAGAGAGAAAGAAAAAATAATTAAAAAAAGAAAACAAGAAAGAAAAAAAAACTAACTAAAATATGGGACAAAACATGAAATAATTGTTGTAAAGTTGGTTCGTTTTGTTTTTTTTTTAGCGAAATGAAAGTTTATAAATAGTGTCGACGCTTAGTAGTTACTAAGGTATATTTACAACGTAAATGAATAAATGTAAGATGAGAGAGAAAGAGAGAGAGAGAGAAAAAAAAGATGCAAAACGGAATCTCCTGAGATTTTTTGTTTTTTTGACCTTTGACGAAAGAGAAATATTAAAATAAAAAAAGCTCGACTATAAACTCTTCATCTGTGATGGATATTAAGATTTATACGCGCGGTGAAGAATTATGTATTTGTTAAACAGAAAAAAAAAATTTTTTTAGCGCGGGAAAACTCGGCACGCCATGTTTGATTCGTGACGTCACTATGTCAATGACATTAGAAACAAAGTTTGTGGTAGGAACCCGTGGAAGACATTCGACAGAAGTGAACCAAGAAAAAAAAAATTTGATACTAAATTGATTACTAAATTGAAAGACTGAGTGAGAAATATTTTAAAGAGCTAGTTAAATTATATTAAATTAGCGGACCCGACAGAAGTTGTCCTGCGAAAATTCGTTCAAGTCGATCCATCCGTTTGGTTGTAGTATAGATAAATAACCTAGATACCGACT |
| *Casein kinase II alpha* | TGGCAGCACTGTCGCGTTCGTTTGTAGAGTAGAGTAGGCAGATGGATGGATAGGGATAGGTGGGCGTCCATCTTGAAGTTTTGCTGGGTTTGAGCCGGTTTCGTTGAAAAAAAAGTGAATTATTTAATTTAGTTTTAACTTTAATTATTAGTATCAAAAAAGTTCTATGGATTCTGAAACTGTTATATAGTGTAGAGTAGGAATAATCACAGTAATGTTTTAGTAACGCTACTCAAGACGTACGCGATTTCCGGAAAGACTATCGCAGATAAAACCTTTGGTTTACAAAGGCACAGTACGATGGCAGTACCTAGTAGAGCGAGGGTCTACGCTGATGTGAACTCACAACGTCCAAGGGAATATTGGGATTACGAAAGTTATGTAGTTGACTGGGGCAACCAAGAAGACTATCAGTTGGTTCGCAAGCTGGGTCGCGGGAAATACAGTGAAGTATTTGAAGCAATAAATATTACGAATAATGAAAAATGTGTAGTTAAAATATTAAAGCCTGTAAAAAAGAAGAAAATTAAAAGAGAAATAAAAATTTTAGAAAACTTAAGAGGAGGCACTAATATAATATCTTTACAAGCTGTAGTCAAAGACCCTGTCTCGCGTACACCTGCACTTATATTTGAACATGTGAACAATACTGACTTTAAACAGCTATATTCGACATTGTCAGATTATGATATAAGATACTACTTATATGAGCTTTTAAAGGCATTAGATTATTGCCATAGTATGGGGATAATGCATAGGGATGTGAAACCTCATAATGTGATGATTGACCATGAACATAGAATGTTACGCCTTATTGACTGGGGGTTAGCTGAGTTCTACCATCCGGGGCAAGATTATAATGTTCGTGTTGCTTCTAGATACTTTAAGGGCCCTGAACTTTTGGTAGATTATCAAATGTATGATTATTCATTGGATATGTGGTCACTAGGATGTATGTTGGCATCCATGATTTTCCGTAAAGAGCCATTCTTTCATGGCCATGATAATTATGACCAGCTTGTACGTATTGCGAAAGTACTGGGTACAGAAGAATTGTTTGAGTACTTGGATAAATATCATATAGAACTGGATCCTCGGTTTAATGACATACTTGGCAGACACTCACGTAAGAGATGGGAGCGATTTATACATTCAGAAAATCAACATCTTGTATCACCAGAGGCACTGGACTTTCTTGACCGTTTACTGCGTTATGATCATTATGAACGCTACACTGCTCGTGAAGCTATGGACCATCCATATTTTTATCCAATCGTAAAGGAGCAGGGTCGAATGGCAACTTCCAACTCACCTACTCCTAATGCACTGCAAGGACCAGTAAATTCTGCAGAGTAATCACAAGAGTCATTAACACTCTTATTAATACTATGTATTTATATATACTTGTACAAGATTCCAGAAGAGACATTGTAAAAAAAACATCAACAAGAGACCGCTGCTTTTGTAAATACACAGAGAAAAAGAGAGATATGCCTACTCTGTACAACTGACTATACAGATTGATGTGATTCCAATTAAATAAGTTTTGGAAAGACAATATAACTTAGTGTTTAAATAATTAGTAAAAACTGGTTTATTTGTTGTTAAAGTGTTTTATCTCTCCTATAAACTGCTGCTGAAACAGTTCTGTGGAAGTGTTCATGTATTTGAGTTGTCGAGTATCTTGTGGTGGGTGGGATTCAATATGTCCAAATTAAAAAACACGATTTTTTGTACGTGAAATTCGATATTAATTTTATTGAAACAAAATAATATAAATATTAAGAACATCGAGTTTCATTGCACTTGATCTTTTGCATTTATTAACCTATGGTATTTGAAAAATGTAATTACTTGTATAATATACTACTGAGAGCATTACATATGATTTGAAATGCTACTTTTAGCAGTTTTTTCGTTGGTACTTGTTTAAAATTTTGTATTTATGTAATAGTAGTAAAAATGTAGATATGTGTGATATAAATACAAAATTTTAATATTAACTCAATAGTTTTTCTTTTTATATTTTTTTTTATAAAAAGAGACCAGTACTACCTACATTTGACAGTGTCAGAATATTGTTTCTATTAGTATATTTTAATAAGTTAATTTAGCATCCTTATACATAATTATTATACATAGCTGAAATCAAGTCGTACCTTCGAGCGAGAAAGAGTGAGCCTCATTGAGTATAATTTATTTTATTGTGATAACTGCAAGTTGTTGCATAAAATTTTCTTAAATAAACAATACTAACTTTGCCTGTAAATGTTTCGATTATTTGTGTTTGAAGAACAAAAAATAAACCTATACTTTTTGTCTCATATTTTTTGGTAAGATAGTGATTGAACTGGATAACAAACATTTTCATACCACCAAGAACCTACTTCTAG |
| *Casein kinase II beta* | CGTGGCTAATCAAGGCACGAAAAGGTGAGGTTTGTCGTCATTTTATCTCAATATTCCATGATTACTGCGAAAAAAAACACATTAAATCTTTGCCAAATTCGTTAATTTACTGTGAAATTGTGAAAAACAGCTTTGAAGACATAAAATAACAGAAAAATGAGCAGCTCCGAGGAGGTATCCTGGATCGCTTGGTTTTGCACGCTTCGAGGCAATGAGTTTTTCTGTGAGGTTGACGAAGACTACATAAATGATAAATTTAATCTAACCGGCTTAAATGAGCAAGTTCCACATTATAGAGAGGCGCTGGACATGATTCTGGATCTGGAGCCAGATGATGACATTGAAGACAATCCAAATGAATCTGATCTGGTGGAACAAGCATCGGAGATACTATATGGATTGATACATGCCCGGTACATATTGACCAATAGGGGCATTAGTCAAATGTTGGATAAGTTCCAGTCTGGGGATTTTGGATATTGCCCACGTGTATACTGTGAATGCCAACCTATGCTGCCAATTGGTTTGTCGGACGTCCCCGCCGAAGCGATGGTGAAGCTGTACTGCCCGCGCTGCATGGACGTGTACACGCCCAAATCCTCGCGCCACCACCACACCGACGGCGCTTACTTCGGCACCGGCTTCCCGCACATGGTGTTCATGGTGCACCCCGATAAACGACCAAAACGACCTGCCTCTCAATTCGTACCAAGACTATACGGGTTCAAGATTCACCCGCTCGCCTACCAAATCCAGCAGGCGGCTGCGAACTCCAAGCCGCCGCAGCGCAGCCTGTCGTACAACAACGGTAAACGTTAGGCCTCAGCCGCGCCGACTCAACACACATCGCGGAACATTTTGGATCCGATCGTAAATGTTGCAGTCGGAATCACGTACATTTAATTGTCCTTAGGAATAGGAACTATAATGTTAAGATATTGATAAGAATTTCTAAAAATATACGAGCATGGGTAACGCAACGTTATATAAAATGGGATTAATTTTTTCAATAAAATTTATTGTTATTTAACTTTACTTCATAAATATGTATTAATAACATGAAGTTGAATAGAGATGTTTCTAGTAATTTGTGTTAGTTTCAATCATATTCGAGATTATGTGATTAGACACGTTGCTATAACTAAAGAAACAATAGAAACAAATATTACTGCACAATAAAAATATATATCTATCTTACCTTTTATAAATAAGGCTTTGTTAATTTCAGTTTGGCCAATAATTCGAATCTTTATTTCGGTCCAAAATGTTCCGCCTACGATATATTACGATAAACGCCAATATGAATAATAACCCGAAAGCCTGCATAATCAAAGGATTACATTAGTCTTCGAATAAAAAAGAACGTTTGCAGCCTAGTTGGAGAATCATAACATAATAACTAAAAGCTATTTTGATTTAACGCATAACCATAATTCATGAACTTTTAAACCGAATTCACCGCGCCCAAACTATATTTTAGTTCTGACGACAAATTGATACTAGGCGGCACGTTTCTGCACACCGATAGCCACCACATATGGCTTTTTAGTTTTGTACTAGAATCCGCAATCTGCCGCAAACCTCACAGACCGTTCATTTACGTCAGACACTAGGATTTTAGACTAGATCTAGACATGTTTTTCGTACATGTACGCTGGTGGTAGCCAAAAGGGCTATTCCAGCTATGCGTGAACGGGGATCTAGACACTAGAATAAAATGTTAAATTAAGAATAAATCTTTACCTACAAAGTTAATTTAGGAGGGTAGCTTCCTTGAATAAAAATCAAAAAATGTCAAGCCAACACGGCTGATTTAAAATAATAATATTCTTTAAAAACAAAAACAAGTTTTTTTAACTTCACAAACTTAGATATTGCTCCTACAAAACAAATGTTATTGCATTATTAGTTAATACATTAAAACATATTTTTTTTTATTTATTCCACATGGCAAGTAATTATTGGAGAGTTGTTATTCCACTCTATAACAACGTCAATAACAAGAGTGGCACAGGCTGTTTCTGTATTCACTAATTATATATAAATCAGTTCTTATTGCTTAGTAGTAGTTTGTTTAAAACCATTTGTTTTAAACACAAATTGTCCTGATAGTAAACGCTTGATGTGTTATATTTTCAAATAATCTTGTATCTCAAATCATTACAGGATAGTTCATTGTGTAAAAATATGAGCCTAGATTTGGTATAATTCAAGTAGCCTTTTGTACTCAACATGTAGAATATATTTTGACTCATTGCACACTGTCTCATCTTTTTTTATACTTTTGGGTAAATTTTATAATTTTTAGTTTTATTTTTTATAACAGGATTAGTAGGATGAATAAAGTAGGTAAGTAGGTACATATTGGAGCAGTCCATATTATCCACGGGGGGGTTTTATGCATTATTTTGCCCTTGTATTGGTTCCAATTATCAATAAATATTAAGATTATTATTGGCATTTTTCTTGCTTCCCCTTGCATGTCAACCATCCTTCTGCTATTCATAGATAAAAGCACTTACCTTGGTAACTTGTATAGATTAAACAAAATAAAGCTATATGTTTAAATGTGCTGATTTTTTTTTAAAAGGTTCCAAATTCGAAATTGAGCAAAACATATTTACATTGATGTTTTAAATAAATGAAACTAGC |
| **Chemosensory protein genes** | |
| BmCSP1 | CTGAATCTGAGTCAAGTGTCTGGGTGACATCGCTTTTGTGTTGATTAAAACCATTGCAGACATGAAGTGTCTAACAATTGCTGCACTTTTATTCGTGGCGGGCTTATCGATTGCCGAGAAGTACACTGACAAGTATGACAACATAGATGTTGATGAAATTCTTGAGAACCGAAAGCTACTGGTTCCTTACATAAAATGCGTATTGGACGAAGGCCGCTGCACTCCCGATGGGAAGGAACTTAAAGCTCATATCAAAGACGGAATGCAGACAGCGTGTGCGAAGTGTACAGATAAACAGAAGGTGTCAGCCAGAAAGATCGTTAAACACATTAAGCAGCACGAAGCTGATTACTGGGAGCAGATGAAGGCCAAATATGATCCCAAAGATGAATTCAAAGAAATCTACGAGGGATTCCTCGCTGGTCAAAACTAAACTTGTTATAAATGCTTGCGGTGGTACAACAGAATAATTGTTTTTTTTTTGTAAGAAATCATAAATGGCTTTTGGTCTGTAAATTCGAACCATATTTAATTTAATTGTTGTTGATTTTCCCGTTTGTTTCTTTAAGTAAACG |
| BmCPS3 | CAGTTAGCTCCTGTACAACGGTATTATCTTACACACCATCAATATGAATTCCCTCATCGCATTCTGCCTGTTTGCCGTTTTGGCCGTGGCTCTCGCCAGACCTGACGACAAGTACACCGATCGCTACGACAACGTTAATTTAGATGAGGTCCTCAGCAATTCTCGACTTCTTCAGCCTTACATCAAATGTATCCTCGACAAGGACAGGTGTGCCCCTGACGCTAAGGAGTTGAAGGAACACATCAGGGAGGCACTCGAGACCGAATGCGCGAAATGTACCGAAGCCCAGAAGAAGGGTACTCGACGTGTTATCGGCCATCTAATTAATAACGAAAGCAAATCCTGGAATGAGCTGACCGCTAAATACGACCCCGAAAATAAATTTACAGCCAAATACGAAAAGGAGCTTCGTGAAATCAAAGCTTAAGACACAAATGCTAAACTAAGCTATGAAATGTGCTAAACAATGAAATGTGATATTTTTCGAATATTAATAATGAATAAATAATTAATACAAA |
| BmCSP4 | CTGCAGACCACTGGAATCACTTACGTGCTGAGCAGGATCATCAACCATGAAGGTCCTTATAGTTTTGAGCTGTGTACTCGTGGCCGTACTTGCTGATGATAAGTACACTGACAAATATGACAAAATTAACTTGCAAGAAATTTTGGAGAACAAGAGGCTCCTGGAGTCGTATATGGATTGCGTGCTCGGCAAGGGCAAGTGTACTCCCGAAGGGAAGGAACTGAAAGATCATCTTCAAGAAGCTCTAGAGACTGGTTGCGAGAAATGCACTGAAGCCCAAGAAAAGGGCGCTGAAACTAGTATTGATTATTTAATCAAGAATGAACTGGAAATTTGGAAGGAGCTGACAGCCCATTTCGACCCCGACGGCAAATGGAGGAAGAAGTACGAAGACCGCGCTAAGGCCAAGGGCATCGTCATCCCTGAATAAAACTTCAATAAAGTTGAACACTAACTAGAATTTTAATTCAACAACGGAATCCTAAATTTTTTATTGTTATGAAATCTGATTTTGTACTTTTCGTTTTTTAAATAAATAAATTTCTTAATGTAAA |
| BmCSP5 | AAACTTTATTGTTTTTGTATCACTAAGGTGGTGATTGAGCTATCGTACACTACTACTCTTGTGATACAAAAACAATAAAGTTTCTCAGAATGAAGACCGTTATTGTTTGCCTGCTTGCCTTGACGGCTGTGGCCCTCGCTCGCCCTGAACAGTACACAGACAAATACGACACTGTCGATCTGGACCAGCTCATATCTAACCGTCGGCTCCTCATTCCCTACGTACATTGCATCCTCGAAAAGGGCCAATGTACCGCAGAAGGCAAGGAACTTAAATCTCACATCAAGGAAGCTCTCGAGACCAACTGTGCTAAATGCACCAAAGCGCAAAAGGGTGGAACCGAAAAGATGATTGGTCACCTCATCAACCACGAAGCCGAGTTCTGGGAGGAGCTGAAGGCCAAATACGACCCCACCAACGAGTTCACAAAGAAATATGAAACTGAACTCAAACGCGTTACAGCTTAAATTCCTTTGAAAAAAACTAACAGCTCAATATCATGTAATAAGATATATGTATTTAGTTGATTTAATTTTCAATTCGACACACATTTTTTTTTCAAAGTGCCTACGAAAATATGTAATTGGTATTATAAATATTTTGCAAAAGGCTTGTGATGTTAGCTGTTATTATAAGTTTAAGATT |
| BmCSP7 | TGCTTAATGTGAGATTGCGTCGTGACTTTGATTGTTATTTCATTTCCATTCTGGTTTAAGAGCTCGGCGTGTGCAGAGTATAGAATAATCTTCCGAATCAATTGGAGACTATAATTCAAGATGAAGGGTTTCTACGTGCTGTGTTTTGCACTGTTCGCTGCTGTCTACTGTAAAGAGACATACAGCTCGGAAAATGACGATCTGGATATTGAAGCTTTGGTGGGAAATATTGATTCTCTGAAGGCCTTTATTGGATGCTTCTTAGAAACATCCCCTTGCGACGCTGTTTCCGGGGATTTCAAAAAGGACATTCCTGAAGCTGTGGCAGAAGCATGTGGCAAATGTACTCCAGCCCAGAAACATCTATTCAAACGTTTCCTTGAAGTCGTCAAGGACAAGCTACCTCAAGAATACGAAGCCTTCAAAACTAAATACGATCCCCAAGGAAAGCATTTCGATGCTCTGTTATCCGCCGTCGCTAATTCTTAAGGAAATCAATTTTTAATCTTCCCCGCTGTTTTGAATTTGCTCGCCGATATGAATAATTTGTACCTTAATTAAATTTGTTTCTGTGCTTATTGTCGTTTTTATTTTGGTAATCAAAATACTACATA |
| BmCSP8 | AACATGAAGACGATCTTGATTTTATGCGCGTTAGTATCGGTCGTTGTTTGCCGACCGGAAGAATACTACAGCAGTCAATATGATAATTTTGACGTCGAGCAGCTGGTGGGCAACTTGCGCCTCTTGAAGAACTATGCCAAGTGCTTCCTCGACCAAGGCCCCTGTACCGCCGAAGGAACAGAATTTAAGAAAAGAATCCCCGAGGCGCTTAGAACTAAATGCGCCAAATGCAACCCGAAACAGCGTCATCTGATCCGTACAGTGGTAAAGGCTTTCCAGACTAAACTGCCTGACCTGTGGGAAGAGCTTGCCATAAAGGAGGACCCCAAGGGCCAATACAAACATGAATTTACTGCTTTCATCAACGCCATGGACTAAAGAGGAATGCCTACACGAAACTAAATAACAAAGCTCGCTATGAAATATTGCCATGATACTTTGAATGGGTTCTTTGAAGCAAACGTGCGCTAAAATGTATTGTTGCAACATCACATTAGTATATTAAAGGATATAGAGTTAAGGATGCACATATATGTTAATTTATTGAACAAAATAAAAC |
| BmCSP9 | GTGAAGCTACGCTAAAAGAAATGAAGTTCGTCCTCGCTCTCATCGCTCTGGCTGTAGTGGTGGCCGCCCGGCCAAATGACGATCTATTCTATGACAAGAAATACGACAACTTCAACGTTGACGAGATTATTGACAACCCAAGACTGCTTAAAGCGTACACATTCTGTTTTAATGACAAGGGAAAGTGCACAGCTGAAGGAAACGACTTCAAGAAATGGATCCCAGAAAGCTTGCAGACATCGTGCGGGAAGTGCTCTGAAAAGCAGAAGTATCTAGTGGCCAAGTTCGTCCACGCCATCAAGGATAAGATGCCTGACGAATTCGATATCCTGCGCAAACTGCACGATCCCAAAGGGGAATACACCGAGAACCTTGATAAATTCCTCGAAACTTACGGACATTAAATTATGATTTAACTTATTCGCTTCATGATAATGTTACCTGTGACTTCATAAATAAATGTCATTACAGT |
| BmCSP10 | GGATAAATAATTAACAACCTACAATGAAAATACTGATCATAGTAGTGATGGCATGTGTGGCGGTCACTTGGGCGCGTCCGGAATCGACCTATACTGATAAATGGGATAACATAAACGTCGATGAGATCCTCGAGTCCAACCGACTCCTCAAAGGTTACGTCGACTGTCTCCTAGGCAAAGGTCGTTGCACTCCAGACGGTAAAGCCTTAAAGGAAACTCTTCCTGATGCTCTTGAACATGAGTGTGTTAAGTGTACTGGGAAACAGAAGTCGGGAGCTGACAAAGTCATTAGACATTTGGTAAATAAACGGCCGGACTTATGGAAGGAGTTGGCTGTTAAATATGATCCCGACAACATCTACCAAGCGAGATACAAAGACAAAATCGATGCCGTCAAAGGCTCCGCTTAGAACGAAGCTTAGATGCACATTTTGGCACGTGGATCGAATTAAAACTGCAGCTTTAATGACGTAGTATTAGTAAAAAAAGCTTTAATTGGATTTTCTTTAATAAAACATATTTCAAC |
| BmCSP11 | GCGTTTTATGGGGATATAATAGAATTTCATTCCACCTATATTTTTGGTCCTATATTGTATTGTCAGTAAAAATGAAACTCACTAGTTTTCTTCTAGTTGGAATGGCTATGGTGTCAGCAGAGTTTTATAGTTCCCGCTATGACGATTTTGATGTCAAGCCATTAGTGGAAAATGATCGAATTTTACAAAGCTACACTAATTGCTTTCTTGACAAAGGACCGTGTACCCCAGATGCCAAGGAATTTAAAAAAGTGATACCCGAAGCTCTGGAAACAACTTGCGGGAAATGCTCGCCAAAACAAAAGCAGTTGATTAAGACGGTGATTAAAGCTGTTATCGAACGCCATCCCGAAGCGTGGGAGGAGCTTGTTAACAAATATGACAAGGACAGAAAATTTAGACCATCTTTCGATAAATTCATTAACGAAGACGACTAACTTTAGCGGCTTGGCTCTGCCCCTGGC |
| BmCSP15 | GGTGAACACATTACGATCATGATCGAGAACTTTTATTCAAAGTGTACGATTAGTAAATCTGTGTTATTCTTATGTTTAATATTTTTACCGTATGCCCTGAATCAGAAATATTACGATTCGCGGTACGATTATTATGATATCGATCATTTGGTACAGAACCCGAGGCTATTGAAAAAATATTTGGATTGTTTTTTGGGTAAAGGACCTTGTACGCCTATAGGAAGACTGTTTAAACAAGTCATGCCTGAAGTGATTACTACAGCCTGCGCCAAATGTACTCCGACGCAAAAGCGATTCGCCAGGAAGACGTTTAATGCTTTTAGAAGA |
| BmCSP16 | ATTCTCCAACCAGACTCGGTTAACGCTTCACCGATCACTAGAATCGTTTGTGTAATATATTTGTAATCATAGTTCTCGAGATCCGGGTCACTCGCTCCCGATCAGTAAAAGACACGACCCGTGCAAGAAAGTAAAAGTGATCGTTTCCACGTTCGTCACGATGAAGCTGAGTTATTTAGGACTGCTCGTGCTGGTGGTTGTCTGTGCGGCGCAACAGAATCGGCCCCAGGTCACAGACACAGCTTTGGATGAAGCACTCAATGACAAGCGATTCATACAGAGACAGCTCAAGTGTGCACTCGGCGAGGCACCCTGCGACCCTATCGGAAAACGTTTGAAAACTCTGGCACCGCTCGTTTTGCGCGGGGCTTGTCCTCAATGCTCACCACAAGAAACGAAACAGATTCAAAAGACACTTTCGTACGTGCAGAGAAACTTCCCGCAGCATTGGGCTAAGCTTGTCCGCCAATATGCCGGCTAAAATACTTTCGGATTAAATGTAAAATTTTAGTGTAAAAATGGTGCTAGGTTGTTAATTAAAATTCAATTATGCTATACCTACTAATCGAGTGGGCCAACGCGTGTTGTAATGAAATATTTTATGGATCGTACATATGCCACCTAAGTTTGTGAACGTACTTATAAAGTGATTTAAGTTCGGTACTAATTTAAAAGTATTAGTTTTTTTATTCGTTAGTATCGAAATCAAATGATTTCAAGGATAAAAATATCACAACGGTTGCACTAATGTGTGTTTCGTCATCTTAAAGAAACCTACACTGGTGTGAGAAGAATTTGGAGTTCATTTGGTCTCGAAAAATAATTATATAATTGAAAATATTTTATACAAATGTTAATTATTAATTTTTTTATTTAATTACTTATTTTGTTTGCCTACATCAGATGCTTTTAAAAATTTAAACCAAAATTTAGTGAGCTAAAACTTAAGATTTAATTATTTAAAGTAATG |
| **BmCSP17** | TGAACTCGAGTCGCTACGAGACTGCCGTTCAGGCGTAACCCGAATTCAACCTGTTGGAAATATTTTTAATCCCATAATAACTCGATTGTAGATGGTTCTAGCATCGATTACGGACAGCACGAACGAGATATCATTTTGAAATGAAGAATATAGTTTTCATTGGTGTATGTACGTGTCTCGTTTTAACTGTGTTGGCGGCACCGCAGATGTCGGATGCACAATTAGAAAAAACCCTTGCTGATAAAGGCACAATGCAAAGACATCTCAGATGTGCACTTGGTGAAGGTCCCTGCGATATGGTGGGGAGAAGATTGAGAACGTTGGCGCCGTTTGTGTTGAGAGGCGCATGCCCTCAATGTTCCGTGCAGGAGTCGCGGCACATTCGCCGCACACTAGCATACATACAACGTAACTACCCATGGGAATGGGCGAGAATCGTACGACAATACGGCTGAAGATTTAATAAAATTTTAAATTGACGTTCGAATTCATTTATTCAAATTCGATTTACCGCGCTTTTTATTATTCGCGCCTTTTTTCATTTTATGTCTAGTATAATATACTTACATACATACTTTTATTAACTTTCAATAAATAAGGACGATAGTAGGTAAATGAACAAAAATAAATTCAATATGACCCGTATAAATGAGGTTGCCTATTCGTTAATAGCGTCACAATCTATTGTTTGTAACAAGCAAGAAACTATTGAATTAATAGCCAATTTTTCCATAGCTAGTTAATTAATGTTAATCAATAAATAATTAATGAAGTTTCACTTGTGCATAACAGCGAGTCAAGGTGTCATTATTTCAAAACTATAATTAGGTTATAATTCGTTCAAAATATTTTTTCTTATAAACGGTATATATACCTTATGTGTATGAGTGACAGTAATAAATATTAAATGATAAA |
